# Can resistance training alone or resistance training combined with aerobic training improve arterial stiffness, endothelial function, and other vascular function indicators in adults with hypertension or overweight/obesity-related vascular risk? A systematic review and meta-analysis of randomized controlled trials

**DOI:** 10.3389/fcvm.2026.1835366

**Published:** 2026-06-24

**Authors:** Yanhao Wang, Zhengnian Lin, Ji Zhu, Wei Gao, Zhiheng Li, Xiaoyan Li, Ming Li

**Affiliations:** School of Physical Education and Sports Science, Fujian Normal University, Fuzhou, Fujian, China

**Keywords:** adults, aerobic training, hypertension, meta-analysis, obese, overweight, resistance training, systematic review

## Abstract

**Objective:**

This systematic review and meta-analysis synthesized evidence from randomized controlled trials to examine the overall effects of resistance training alone or in combination with aerobic training on arterial stiffness, endothelial function, and other vascular function indices in adults with hypertension or overweight/obesity-related vascular risk.

**Methods:**

Following PRISMA guidelines, PubMed, Embase, Web of Science, the Cochrane Library, Ovid, CNKI, Wanfang Data, VIP, and CBM were searched from inception to January 2026 for randomized controlled trials examining resistance training alone or combined with aerobic training on vascular function in adults with hypertension or overweight/obesity-related vascular risk. Random-effects meta-analyses were performed using *Hedge's g* with 95% confidence intervals (CIs), and heterogeneity was assessed using the I^2^ statistic. Prespecified subgroup analyses and meta-regression were conducted as exploratory analyses to identify possible sources of heterogeneity, rather than to establish definitive moderator effects or optimal exercise-prescription thresholds.

**Results:**

Twenty randomized controlled trials were included. Resistance training-based interventions performed alone or combined with aerobic training significantly reduced arterial stiffness (*g* = −0.18, *95% CI* −0.33 to −0.04; *p* = 0.01) and improved FMD (*g* = 0.70, *95% CI* 0.41–0.99; *p* < 0.0001) in adults with hypertension or overweight/obesity-related vascular risk. However, the FMD finding should be interpreted cautiously because potential publication bias or small-study effects were detected and the evidence base was limited. Exploratory subgroup patterns were not supported by significant linear associations in meta-regression. No significant effect was observed for wave reflection indices, and evidence for peripheral haemodynamic outcomes remained insufficient.

**Conclusions:**

Resistance training-based interventions may improve selected vascular function outcomes in adults with hypertension or overweight/obesity-related vascular risk, particularly FMD. However, the FMD finding should be interpreted cautiously because of potential publication bias or small-study effects and the limited evidence base. Evidence for arterial stiffness was less robust after sensitivity analyses, no consistent benefit was observed for wave reflection indices, and evidence for peripheral haemodynamics remained insufficient. Current evidence does not support the superiority of resistance training-based interventions performed alone over combined aerobic–resistance training, and exploratory subgroup findings should not be used to infer optimal training prescriptions.

**Systematic Review Registration:**

https://www.crd.york.ac.uk/PROSPERO/view/CRD420261335684

## 1 Introduction

Hypertension is one of the most prevalent and important modifiable cardiovascular risk factors worldwide and a major driver of coronary heart disease, stroke, heart failure, chronic kidney disease, and premature mortality (1). The 2024 ESC Hypertension Guidelines further emphasize that hypertension is not merely an elevation in blood pressure, but a clinical syndrome involving multiple organs, including the heart, brain, kidneys, and vasculature, and is accompanied by an increased overall cardiovascular risk. Accordingly, risk stratification and management should extend beyond conventional blood pressure readings to incorporate hypertension-mediated target organ damage and overall vascular risk (2, 3). In the present study, we specifically focused on adults with hypertension or overweight/obesity-related vascular risk, a clinically high-risk subgroup characterized by elevated blood pressure, metabolic disturbances, and an increased likelihood of vascular dysfunction.

In adults with hypertension or overweight/obesity-related vascular risk, excess body weight is not merely a coexisting characteristic, but a key pathological driver of disease onset, maintenance, and progression (4). Global BMI levels and the burden of overweight/obesity continue to rise, and excessive adiposity is estimated to account for approximately 65%–75% of the risk of primary hypertension. In particular, visceral fat accumulation may promote both blood pressure elevation and vascular injury through multiple interrelated mechanisms, including renal compression, increased tubular sodium reabsorption, activation of the sympathetic nervous system, overactivation of the renin–angiotensin–aldosterone system (RAAS), insulin resistance, chronic low-grade inflammation, and oxidative stress (5–7). In other words, obesity is not simply a coexisting condition in hypertension, but a fundamental pathological substrate underpinning its development, persistence, and progression to cardiovascular complications (8). Importantly, the combined burden of obesity and hypertension extends beyond the conventional haemodynamic load, and is more profoundly reflected in persistent structural and functional vascular abnormalities. Increasing evidence suggests that hypertension may be understood as a disorder accompanied by systemic vascular injury, in which endothelial dysfunction, increased arterial stiffness, and chronic vascular inflammation are closely intertwined and jointly contribute to its development and progression (9, 10). Endothelial dysfunction is generally regarded as an early event in vascular injury and is characterized by reduced nitric oxide bioavailability, impaired vasodilatory responsiveness, and heightened inflammatory and prothrombotic activity (11). Increased large-artery stiffness further exacerbates pulsatile load, promotes the premature return of reflected waves, and impairs microvascular perfusion, thereby contributing to left ventricular hypertrophy, cerebral small vessel disease, and renal injury (12). In individuals with obesity-related hypertension, this process is often further amplified by adipose tissue endocrine dysregulation, oxidative stress, and metabolic disturbances (13).

On the basis of these pathophysiological mechanisms, arterial stiffness and endothelial function may be regarded as the two most central outcome domains in studies of vascular function in adults with hypertension or overweight/obesity-related vascular risk (12). Carotid–femoral pulse wave velocity (cfPWV) is widely regarded as the gold-standard measure of large-artery stiffness and has been consistently used in hypertension research across Europe and North America to characterize vascular ageing and cardiovascular risk (14). More recent evidence further indicates that elevated cfPWV is independently associated with an increased risk of future cardiovascular events, underscoring its value not only as a mechanistic marker, but also as a clinically relevant prognostic indicator (15, 16). Similarly, flow-mediated dilation (FMD), a well-established non-invasive measure of endothelium-dependent vasodilatory function, has also demonstrated predictive value for cardiovascular events (17). Because arterial stiffness and endothelial function reflect, respectively, the mechanical properties of large arteries and endothelium-dependent vascular responsiveness, they are more capable than conventional blood pressure indices of directly capturing whether exercise interventions act on hypertension-related vascular injury itself. They can therefore be considered primary outcomes for determining whether an intervention confers vascular benefit (18, 19). At the same time, wave reflection indices such as the augmentation index (AIx), together with haemodynamic parameters including peripheral blood flow, peripheral vascular resistance, and systemic vascular resistance, may provide a more nuanced characterization of vascular load, arterial wave transmission, and peripheral resistance status (20, 21). These measures are therefore better regarded as complementary functional markers that supplement the core vascular outcomes.

In the management of hypertension, lifestyle modification remains a cornerstone of treatment (22), and regular exercise is among the most evidence-based and practical core strategies (23). The 2024 ESC Hypertension Guidelines emphasize that exercise training should be incorporated into comprehensive hypertension management, while the 2023 American Heart Association scientific statement on resistance training further highlights that resistance training not only enhances or preserves muscle mass and strength, but also improves blood pressure, insulin sensitivity, lipid profiles, and certain non-traditional cardiovascular risk factors, with an overall favorable profile of safety and feasibility in adults both with and without cardiovascular disease (3, 24). Contrary to the traditional view that hypertension management relies primarily on aerobic exercise, the role of resistance training in individuals with hypertension and elevated cardiovascular risk has been increasingly reappraised in recent years (25, 26). Particularly in adults with hypertension or overweight/obesity-related vascular risk, the potential of resistance training to preserve muscle mass, enhance functional capacity, optimize body composition, and improve insulin sensitivity may confer additional clinical relevance beyond aerobic training alone (27, 28). More importantly, resistance training alone and resistance training combined with aerobic training should not be regarded as homogeneous interventions. These approaches may differ in the composition of the training stimulus, exposure to haemodynamic shear stress, pathways of body composition improvement, and regulation of peripheral resistance, and therefore may not exert identical effects on arterial stiffness and endothelial function (29, 30). From a physiological perspective, both resistance training alone and combined resistance plus aerobic training warrant separate evaluation because they may influence vascular function through partially overlapping, but not entirely identical, mechanisms (24). On the one hand, resistance training may indirectly attenuate overweight/obesity-related vascular injury through improvements in body composition and metabolic health, including preservation or increases in lean mass, reductions in fat mass, improved insulin sensitivity, and potential attenuation of chronic low-grade inflammation (30–33). On the other hand, the periodic increases in blood flow and shear stress induced by exercise may upregulate endothelial nitric oxide synthase activity, enhance nitric oxide bioavailability, and improve vasodilatory responsiveness (34–36). In parallel, training-induced modulation of sympathetic nervous system activity, reductions in peripheral resistance, and improvements in arterial compliance may contribute to lowering arterial stiffness and wave reflection burden (37). Different resistance training modalities should not be regarded as physiologically equivalent, because they differ in contraction pattern, recruited muscle mass, haemodynamic stimulus, and metabolic demand (38–40). Dynamic resistance training produces rhythmic contraction–relaxation cycles, with intermittent vascular compression and reperfusion that generate pulsatile shear-stress exposure and load-dependent pressor responses (34, 38). Isometric resistance training involves sustained static contractions and may therefore induce more localized vascular compression and reactive hyperaemia (39). Circuit-based resistance training, because of its short rest intervals and repeated multi-exercise format, may impose greater whole-body metabolic demand and more frequent fluctuations in heart rate, blood flow, and peripheral vascular tone (38). Resistance training combined with aerobic training may provide a more sustained shear-stress stimulus through the aerobic component and may additionally influence body composition, insulin sensitivity, and peripheral resistance regulation (34, 40). These modality-specific differences may partly explain why arterial stiffness, endothelial function, wave reflection, and peripheral haemodynamic outcomes do not necessarily respond uniformly across resistance training-based interventions (30, 40). Accordingly, their effects on arterial stiffness, endothelial function, and other vascular function indices may not be uniform (41). Because different resistance training regimens may induce vascular adaptations through distinct mechanistic pathways, individual randomized controlled trials often provide limited and context-specific evidence. Therefore, a systematic synthesis is needed to evaluate the overall direction and magnitude of the available evidence on resistance training alone and resistance training combined with aerobic training, while recognizing that the current evidence base may not be sufficient to draw firm conclusions regarding detailed prescription differences.

However, despite the growing evidence on exercise training and vascular health, the specific vascular effects of resistance training-based interventions in adults with hypertension or overweight/obesity-related vascular risk remain insufficiently clarified (42). Previous reviews have often examined broad adult or mixed clinical populations and have commonly pooled aerobic training, high-intensity interval training, resistance training, combined training, and other multicomponent exercise programmes within the same synthesis, making it difficult to isolate the effects of resistance training alone or resistance training combined with aerobic training (29, 40). Moreover, most previous syntheses have focused mainly on blood pressure, body composition, or metabolic outcomes, whereas fewer have systematically integrated vascular function outcomes across arterial stiffness, endothelial function, wave reflection indices, and peripheral hemodynamics (28, 31, 43). Given the limited number of eligible trials and the heterogeneity of intervention protocols and outcome measures, the present review was intended to provide a focused synthesis of the overall evidence and to identify preliminary patterns for future research, rather than to define an optimal resistance-training prescription.

Against this background, the primary aim of the present systematic review and meta-analysis was to synthesize randomized controlled trial evidence on the overall effects of resistance training alone or resistance training combined with aerobic training on arterial stiffness, endothelial function, wave reflection indices, and peripheral haemodynamic outcomes in adults with hypertension or overweight/obesity-related vascular risk (24, 41, 44). A secondary and exploratory aim was to examine whether the observed effects appeared to differ according to broad participant characteristics and intervention-related features, where sufficient data were available (24, 37, 44). Given the limited number of included trials and the heterogeneity of training protocols and outcome measures, these exploratory analyses were intended to generate hypotheses and identify possible sources of heterogeneity rather than to establish definitive resistance-training prescriptions. This work may therefore provide a more cautious and focused synthesis of the current evidence to inform future research and clinical exercise-prescription development in this high-risk population (3, 24).

## 2 Methods

This systematic review and meta-analysis was conducted in strict accordance with the Preferred Reporting Items for Systematic Reviews and Meta-Analyses (PRISMA) guidelines (45), and was prospectively registered in the PROSPERO database (registration No. CRD420261335684).

### 2.1 Information sources

We systematically searched PubMed, Embase, Web of Science, the Cochrane Library, Ovid, China National Knowledge Infrastructure (CNKI), Wanfang Data, VIP, and the Chinese Biomedical Literature Database (CBM) from database inception to January 2026, without restrictions on publication year, document type, or language. To maximize retrieval comprehensiveness, supplementary snowballing procedures were performed in addition to the electronic database search, including backward screening of the reference lists of all included studies, forward screening of articles citing the included studies, and expansion of potentially eligible records using the “Similar articles” function in MEDLINE and the “Find similar” function in Embase. All searches were completed in January 2026.

### 2.2 Literature search strategy

The search strategy was informed by previous systematic reviews on related topics, and the search terms included: (“resistance training”[MeSH Terms] OR (“resistance”[All Fields] AND “training”[All Fields]) OR “resistance training”[All Fields]) AND ((“blood vessels”[MeSH Terms] OR (“blood”[All Fields] AND “vessels”[All Fields]) OR “blood vessels”[All Fields] OR “vascular”[All Fields] OR “neovascularization, pathologic”[MeSH Terms] OR (“neovascularization”[All Fields] AND “pathologic”[All Fields]) OR “pathologic neovascularization”[All Fields] OR “vascularisation”[All Fields] OR “vascularization”[All Fields] OR “vascularisations”[All Fields] OR “vascularise”[All Fields] OR “vascularised”[All Fields] OR “vascularities”[All Fields] OR “vascularitis”[All Fields] OR “vascularity”[All Fields] OR “vascularizations”[All Fields] OR “vascularize”[All Fields] OR “vascularized”[All Fields] OR “vascularizes”[All Fields] OR “vascularizing”[All Fields] OR “vasculars”[All Fields]) AND (“functional”[All Fields] OR “functional s”[All Fields] OR “functionalities”[All Fields] OR “functionality”[All Fields] OR “functionalization”[All Fields] OR “functionalizations”[All Fields] OR “functionalize”[All Fields] OR “functionalized”[All Fields] OR “functionalizes”[All Fields] OR “functionalizing”[All Fields] OR “functionally”[All Fields] OR “functionals”[All Fields] OR “functioned”[All Fields] OR “functioning”[All Fields] OR “functionings”[All Fields] OR “functions”[All Fields] OR “physiology”[MeSH Subheading] OR “physiology”[All Fields] OR “function”[All Fields] OR “physiology”[MeSH Terms])).

### 2.3 Selection process

All records identified through the database searches were first imported into EndNote 21, and duplicates were removed by one reviewer (Y.-H.W.). Thereafter, two reviewers (Z.-N.L. and W.G.) independently screened titles and abstracts and assessed full-text articles for eligibility according to the predefined inclusion and exclusion criteria, with reasons for full-text exclusion documented. Any disagreements arising during the selection process were resolved through discussion and, when necessary, adjudicated by a third reviewer (Z.-H.L.). In addition to the database search, relevant systematic reviews were examined, the reference lists of included studies were manually screened, and potentially eligible studies that might otherwise have been missed were further identified on the basis of the research team's domain expertise. The study selection process is presented in a PRISMA flow diagram.

### 2.4 Eligibility criteria and exclusion criteria

Eligible studies were predefined according to the PICOS framework. Studies were included if they were randomized controlled trials involving adults (≥18 years) with hypertension and/or elevated vascular risk related to overweight/obesity. This broader operational population definition included participants with diagnosed hypertension, elevated blood pressure, prehypertension, antihypertensive medication use, overweight/obesity, or overweight/obesity-related cardiometabolic or vascular risk, as defined in the original trials. The study-specific basis for population eligibility is presented in Supplementary Table S1. Interventions were required to include resistance training, delivered either alone or in combination with aerobic training, with no restrictions placed on training modality. Eligible comparators were non-exercise or usual care controls that did not receive structured exercise intervention. Studies were included if they reported at least one vascular function outcome, including indices of arterial stiffness (e.g., pulse wave velocity, carotid–femoral pulse wave velocity, and the β-stiffness index), endothelial function (e.g., flow-mediated dilation), wave reflection (e.g., augmentation index), or peripheral hemodynamics (e.g., blood flow and systemic vascular resistance).

Studies were excluded if they met any of the following criteria: (1) non-randomized controlled trials; (2) participants were not adults with hypertension, elevated blood pressure, prehypertension, antihypertensive medication use, overweight/obesity, or overweight/obesity-related vascular risk; (3) the intervention did not include resistance training, or involved a multicomponent programme in which the independent effect of resistance training could not be reasonably isolated; (4) no eligible non-exercise or usual care control group was included; or (5) no relevant vascular function outcomes were reported, or insufficient data were provided to estimate effect sizes. For multiple publications arising from the same trial, only the dataset with the most complete information was retained.

### 2.5 Data extraction and transformation

Data extraction was performed independently by two reviewers (Y.-H.W. and Z.-N.L.) using a standardized, pilot-tested data extraction form developed in Microsoft Excel. For each included study, the following information was systematically extracted and cross-checked: (1) basic study characteristics, including first author, year of publication, country/region, journal, and study design; (2) participant characteristics, including sample size, mean age, and sex distribution; (3) intervention characteristics, including intervention modality (resistance training alone or resistance training combined with aerobic training), intervention duration, weekly training frequency, and total number of training sessions; (4) outcome-related information, including vascular function outcomes (e.g., arterial stiffness, endothelial function, and other vascular function indices), measurement/assessment methods, statistical parameters used for effect size calculation, and principal findings; and (5) where a single article reported multiple independent studies or multiple comparison arms (e.g., different training regimens or intervention groups), data were extracted separately for each study or comparison arm to preserve the independence of effect size estimation.

When original studies contained missing data, incomplete reporting, or unclear descriptions, we first contacted the corresponding authors to obtain additional information. If the required data remained unavailable, standard statistical conversion methods were applied, where methodologically appropriate, to transform the reported statistics into a form suitable for pooling. For example, when studies reported 95% confidence intervals or standard errors, these were converted into standard deviations. Baseline and post-intervention values for each vascular function outcome were also extracted, and the pre–post change score (mean difference, MD) and its standard deviation were calculated. In accordance with the recommendations of the Cochrane Handbook, when the correlation coefficient (r) required for calculating pre–post change scores was not reported, we attempted to obtain this information by contacting the corresponding authors in order to improve the accuracy of data handling.

### 2.6 Data extraction

Where studies reported confidence intervals (*CI*), these were converted to standard deviations (*SD*) (46):

$$SD=\sqrt{N}\frac{CI_{\mathrm{high}}-CI_{\mathrm{low}}}{2t}$$

Where studies reported standard errors (SE), these were converted to *SD* (46):

$$SD=\sqrt{N}\;\times SE$$

Baseline and final time-point values for vascular function outcomes were extracted from each study, and the mean pre–post change (mean difference, *MD*) was calculated using the following formula (46):

$$MD_{\mathrm{diff}}=M_{\mathrm{post}}-M_{\mathrm{pre}}$$

The *SD* of the pre–post change scores was calculated using the following formula (46):

$$SD_{\mathrm{diff}}=\sqrt{SD_{\mathrm{pre}}^{2}+SD_{\mathrm{post}}^{2}-2r\;\times SD_{\mathrm{pre}}\;\times SD_{\mathrm{post}}}$$

In accordance with the recommendations of the Cochrane Handbook, we contacted the corresponding authors of the included trials to obtain the correlation coefficients (r values) required for pre–post data analyses.

### 2.7 Risk of bias and quality of methods assessment

Risk of bias was assessed independently by two reviewers (the first and third authors), with disagreements resolved through discussion and, where necessary, adjudication by an additional reviewer. The Cochrane risk-of-bias tool was used to systematically evaluate the included studies across seven domains: random sequence generation, allocation concealment, blinding of participants and personnel, blinding of outcome assessors, completeness of outcome data, selective outcome reporting, and other potential sources of bias. Given the inherently recognizable nature of exercise training interventions, blinding of participants and intervention personnel is often difficult to implement and was therefore not used as a predefined criterion for study inclusion or exclusion. By contrast, blinding of outcome assessors was judged primarily according to whether vascular function outcomes were measured using objective and standardized assessment methods. Each domain was rated as having a “low risk,” “unclear risk,” or “high risk” of bias, and an overall risk-of-bias judgement (low, moderate, or high) was then assigned for each study accordingly. The risk-of-bias summary and risk-of-bias graph were generated using Review Manager (RevMan) version 5.4.1.

### 2.8 Statistical analysis

All statistical analyses and data visualisation were performed in R (version 4.5.0), primarily using the meta, metafor, and metameta packages. Effect sizes were pooled using a random-effects model and synthesized with the inverse-variance DerSimonian–Laird method (47). Between-study heterogeneity variance (*τ*^2^) and its confidence interval were estimated using the Jackson method. Based on the data reported in the included studies, mean differences (*MDs*) or standardized mean differences (*SMDs*), together with their 95% confidence intervals, were extracted or converted as appropriate. Given the variation in measurement units across vascular function outcomes, and in accordance with relevant methodological recommendations (48), *SMD* was adopted as the primary effect measure in the present study. Because most included studies had relatively small sample sizes (*n* < 50), *Hedge's g* was used to correct for small-sample bias and is hereafter uniformly denoted as g. Effect sizes were interpreted according to conventional thresholds: 0–0.2, trivial; 0.2–0.5, small; 0.5–0.8, moderate; and >0.8, large (49). In addition, statistical power analyses were conducted to evaluate the robustness of the overall and subgroup effects and to reduce the risk of type II error arising from insufficient statistical power (50).

Between-study heterogeneity was comprehensively assessed using the Cochrane *Q* test, *I*^2^ statistic, *τ*^2^, and *τ*, in accordance with current methodological recommendations (48). The degree of heterogeneity was classified primarily on the basis of *I*^2^ values, with 0%–25% indicating low heterogeneity, 25%–75% indicating moderate heterogeneity, and >75% indicating high heterogeneity (51). Prediction intervals were also calculated to characterize the likely distribution of the true effect across different study settings and to assess the external applicability of the pooled estimates.

Subgroup analyses and meta-regression were conducted in accordance with the prespecified registration protocol, with emphasis on two broad categories of potential sources of heterogeneity: participant characteristics and intervention-related features. Prespecified subgroup analyses were used to examine categorical variables, whereas continuous variables were evaluated using mixed-effects meta-regression models (52). The moderators evaluated in subgroup analyses included age category, sex composition, intervention modality, training intensity, repetitions, number of sets, weekly training frequency, session duration, and intervention duration, where sufficient data were available. Subgroup analyses and meta-regression addressed related but distinct exploratory questions. Subgroup analyses compared pooled effects across predefined categorical strata, whereas meta-regression examined whether continuous participant- or intervention-related variables were linearly associated with effect sizes. Therefore, statistically significant between-subgroup differences were not interpreted as evidence of linear dose–response relationships unless supported by corresponding meta-regression results. Model parameters were estimated using restricted maximum likelihood (REML), and the final model form was determined by comparing the bias-corrected goodness of fit between linear functions and cubic spline functions. However, because only a limited number of randomized controlled trials were available for several outcomes and subgroups, all subgroup analyses and meta-regression should be interpreted as exploratory and hypothesis-generating. These analyses were used to identify possible sources of heterogeneity rather than to establish causal moderator effects or definitive exercise-prescription thresholds. Relative to conventional maximum likelihood estimation, this approach generally provides greater robustness in modelling frameworks that incorporate both fixed and random effects (53).

Publication bias and small-study effects were assessed by visual inspection of funnel plots and Egger's linear regression test (54, 55). When the *p* value for Egger's test exceeded 0.05, no clear evidence of substantial publication bias was considered to be present. All statistical tests were two-tailed; *p* < 0.05 was considered statistically significant, whereas 0.05 ≤ *p* < 0.10 was interpreted as indicating a statistical trend.

### 2.9 Certainty of the evidence

The certainty of evidence for the primary outcomes was evaluated using the GRADE framework and classified as high, moderate, low, or very low. Because all included studies were randomized controlled trials, the initial certainty rating was set as high. Certainty was then downgraded according to five domains: risk of bias, inconsistency, indirectness, imprecision, and publication bias.

Risk of bias was downgraded when the included studies showed important methodological limitations, particularly in random sequence generation, allocation concealment, outcome measurement, incomplete reporting, or selective outcome reporting. Inconsistency was downgraded when moderate or substantial heterogeneity was present, when prediction intervals suggested important variability in the likely true effects, or when sensitivity analyses materially altered the robustness of the pooled estimate. Indirectness was assessed with respect to population, intervention, and outcome applicability. Specifically, evidence was downgraded for indirectness when the included trials did not fully correspond to the target population of adults with hypertension or overweight/obesity-related vascular risk, when intervention formats were clinically heterogeneous, or when pooled outcomes combined different measurement indices within the same vascular domain. Imprecision was downgraded when the confidence interval crossed the line of no effect, when the number of studies or participants was small, or when the estimate was considered unstable. Publication bias was downgraded when funnel-plot asymmetry or Egger's test suggested possible small-study effects.

Each domain was judged as not serious, serious, or very serious. Certainty was downgraded by one level for each serious concern and by two levels for each very serious concern. All GRADE assessments were conducted independently by two authors, and disagreements were resolved through discussion.

## 3 Results

### 3.1 Literature search results

**Figure 1:**
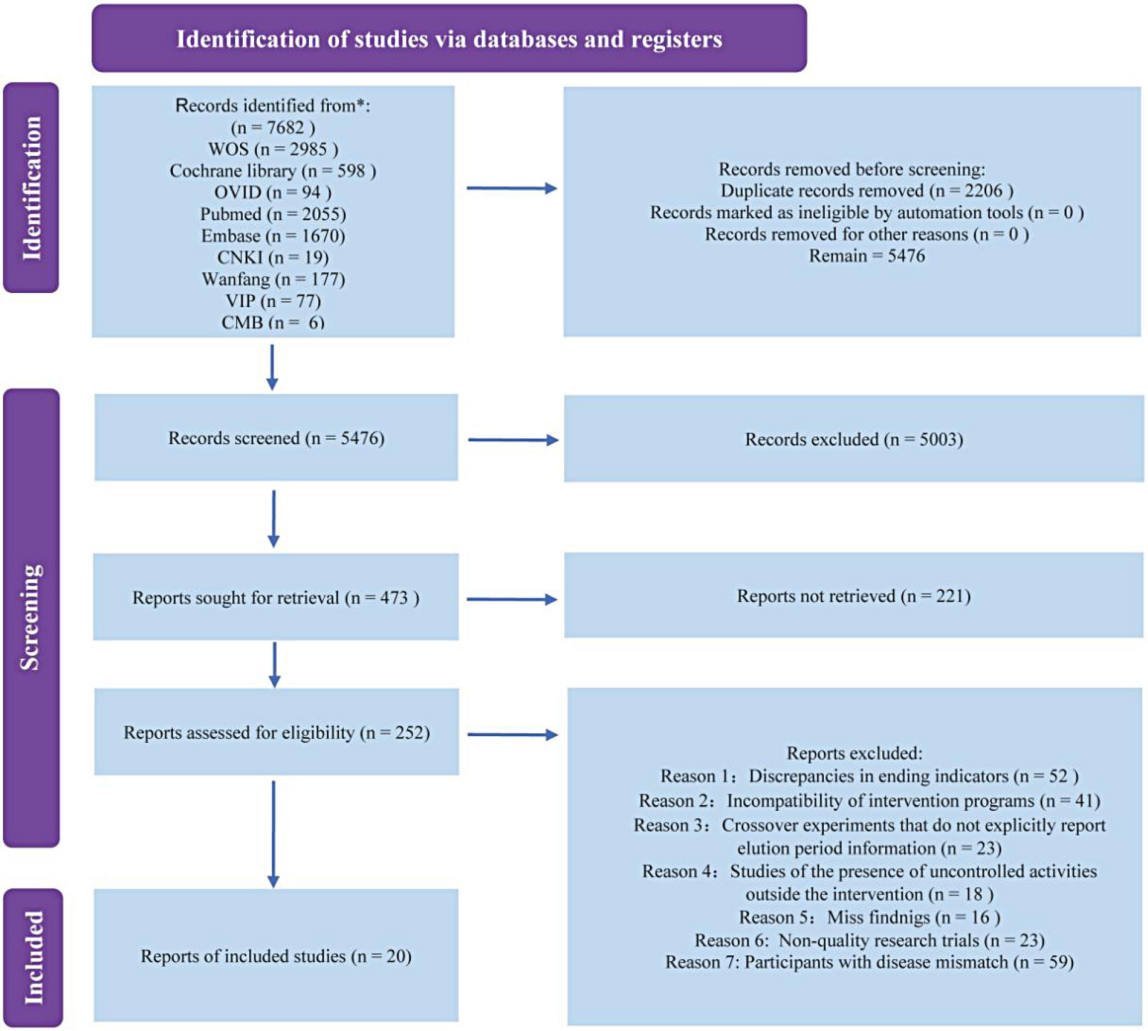
Flow diagram of literature selection.

A total of 7,682 records were identified through the database search. Following stepwise screening and eligibility assessment, 20 studies met the inclusion criteria and were ultimately included in this systematic review and meta-analysis. The study selection process and reasons for exclusion at each stage are presented in Figure 1.

### 3.2 Analysis of basic characteristics of included studies

The 20 studies ultimately included were all randomized controlled trials, and the study populations generally comprised adults with hypertension or overweight/obesity-related vascular risk. Participant age varied substantially across studies, ranging approximately from 21 to 78 years, although a small number of studies enrolled broader age-range samples, such as 18–40 years or 30–65 years. The sex distribution also varied, with some studies including single-sex samples and others enrolling mixed-sex cohorts; overall, however, middle-aged and older adults, as well as female or mixed-sex populations, predominated. Sample sizes differed considerably across studies, suggesting a degree of heterogeneity in both population characteristics and study scale. With respect to intervention characteristics, most included studies examined resistance training alone, whereas a smaller number employed resistance training combined with aerobic training. The specific training modalities were diverse and included traditional dynamic resistance training, isometric handgrip training, circuit resistance training, whole-body vibration training, and brief intermittent resistance-type activity. Marked variation was also evident in prescription parameters, including training intensity, number of repetitions, number of sets, weekly frequency, session duration, and intervention length. Training intensity was predominantly moderate to vigorous, and training frequency generally ranged from 2 to 6 sessions per week, with 3 sessions per week being the most common. Individual session duration ranged from approximately 11–90 min, and intervention duration ranged from approximately 1–52 weeks, although most studies were concentrated within an 8- to 12-week timeframe. Outcome measures were primarily centered on arterial stiffness and endothelial function, with commonly reported indices including pulse wave velocity (PWV) and its variants, as well as flow-mediated dilation (FMD). Some studies also reported augmentation index (AIx) and peripheral haemodynamic outcomes. Overall, the included studies exhibited substantial clinical diversity in participant characteristics, intervention modalities, and outcome assessment. This diversity provides an important basis for comprehensively evaluating the vascular effects of resistance training-based interventions, while also underscoring the need to carefully consider between-study heterogeneity when interpreting the pooled findings (Table 1).

**Table 1:** Characteristics of included studies.

Study	Age (years)	Participants (M/F)		Intervention		Description of Intervention	Intensity	Repetitions	Sets	Frequency (t/wk)	Time (min)	Duration (wk)	Outcome
	Intervention/Control	Intervention	Control	Intervention	Control								
Menêses et al., 2015 (56)	57	19	19	AT+RT	non-exercise	E+R: treadmill walking followed by resistance exercise; R+E: same components in reverse order. Endurance exercise was 30 min at 50%–60% HRR; resistance exercise involved seven exercises, 3 × 10 repetitions at 50% 1RM.	RT: 50% 1RM AT: 50%–60% HRR Moderate intensity	10	3	NA	50	NA	PVR
				RT+AT									
Banks et al., 2024 (41)	54	5/8	5/8	RT	non-exercise	RET was performed 3 days/week for 9 weeks (∼40 min/session) using bench press, hack squat, lat pulldown, leg extension, seated row, leg curl, and plank exercises, with 1–3 min rest and 2 + 2 rule-based load progression.	Progressive based on RM (10RM to 12RM) Moderate intensity	NA	NA	3	40	9	FMD cfPWV
Boeno et al., 2020 (57)	45	6/9	5/7	RT	non-exercise	Supervised dynamic resistance training was performed 3 days/week for 12 weeks, using eight resistance exercises with 2–3 sets of 8–20 submaximal repetitions, 120-s rest between sets, and 3 × 15 abdominal crunches each session.	submaximal load	8–20	2–3	3	45–50	12	FMD
Rodrigues et al., 2019 (58)	60	17	16	IHT	non-exercise	Isometric handgrip training was performed 3 days/week for 12 weeks, consisting of 4 × 2-min contractions at 30% MVC with alternating hands, 1-min rest between contractions, and load readjustment at week 6.	30% MVC Moderate intensity	NA	4	3	NA	12	cPWV pPWV FMD
Farah et al., 2018 (59)	59	5/9	5/11	home-based IHT	non-exercise	IHT was performed 3 days/week for 12 weeks: 4 × 2-min handgrip contractions at 30% MVC, alternating hands, with 1-min rest and week-6 load adjustment; sessions were laboratory-supervised or home-based after initial familiarization.	30% MVC Moderate intensity	1	4	3	20	12	cPWV pPWV AIx
	60	6/12	5/11	Supervised IHT	non-exercise								
Beck et al., 2013 (60)	21	11/4	9/6	RT	non-exercise	RT was performed 3 days/week for 8 weeks, with each 60-min session including seven machine-based exercises, 2 sets of 8–12 repetitions to volitional fatigue, 2–3 min rest, and 5% load progression when 12 repetitions were completed in the second set.	to failure	8–12	2	3	60	8	crPWV fdPWV cfPWV AIx
Yoon et al., 2019 (61)	69	5/12	4/14	IHT	non-exercise	Isometric handgrip contractions were performed at 30% of MVC for 2 min, with 1 min of rest between contractions, alternating hands for two contractions each (four contractions per session). Training was conducted three times per week for 12 weeks under center-based supervision.	30% MVC moderate intensity	5	4	3	11	12	cfPWV AIx FMD
Miura et al., 2015 (62)	71	92		CRT	non-exercise	Supervised group exercise was performed twice weekly for 12 weeks (∼90 min/session), including recreational activities, circuit resistance training with elastic tubes/light dumbbells, and chair-based lower-limb exercises.	NA	15–20	35	2	90	12	baPWV
	72	108		CRT									
McGowan et al., 2007 (63)	62	5/2	7/2	Bilateral IHT	non-exercise	Bilateral IHG, 3 days/week for 8 weeks: 4 × 2-min contractions at 30% MVC, alternating hands, with 1-min rest between contractions; two supervised and one home-based session weekly.	30% MVC moderate-intensity	4	4	3	20	8	FMD
	66	7/2	7/2	Unilateral IHT	non-exercise	Unilateral IHG, 3 days/week for 8 weeks: 4 × 2-min contractions at 30% MVC using the non-dominant hand, with 4-min rest between contractions; two supervised and one home-based session weekly.							
Jung et al., 2024 (64)	78	0/14	0/14	CRT	non-exercise	The circuit training program … consisted of ten exercises: Walking in place, shoulder presses and squats, twist dashes, lunges, jumping jacks, kickbacks, modified push-ups, crunches, hip bridges, and bird dogs.	60%–85% HRR High intensity	NA	2–4	3	45–75	12	baPWV FMD
Franklin et al., 2015 (65)	18–40	0/10	0/8	CRT	non-exercise	Supervised CRT was performed twice weekly for 8 weeks on non-consecutive days, using 8–10 dynamic resistance exercises in circuit format at 80%–90% 10RM, with 8–10 repetitions to volitional fatigue, 30-s rest between exercises, 2–3 circuits/session, and progressive load increases.	80%–90% 10RM Moderate intensity	8–10	2–3	2	60	8	FMD
Dobrosielski et al., 2021 (66)	30–65	25/26		RT+AT	non-exercise	Combined aerobic and resistance training was performed 3 days/week for 6 weeks (∼60–65 min/session), including 40 min of aerobic exercise progressing from 60%–70% to 80%–90% HRR, followed by resistance exercises performed as 2 sets of 10–15 repetitions at an initial load of 60% 1RM.	AT: 60%–90% of heart rate reserve; RT: 60% 1 repetition maximum progressively increased High intensity	10–15	2	3	60–65	6	FMD AIx cfPWV
Fernandez-del-Valle et al., 2018 (67)	24	0/6	0/5	RT	non-exercise	Supervised circuit RT was performed 3 days/week for 3 weeks (∼50 min/session), using seven exercises completed as 3 × 10 repetitions at ∼70%–75% 1RM, with 30-s rest between exercises and 2-min rest between sets.	70%–75% 1RM; HR 70%–85% HRmax High intensity	10	3	3	50	3	PWV
Figueroa et al., 2014 (68)	56	0/13	0/12	RT-WBV	non-exercise	Supervised WBV exercise training was performed 3 days/week for 12 weeks, using dynamic/static squats, lunges, and calf raises on a vibration platform, with progressive increases in vibration frequency, amplitude, set duration, and number of sets.	25–40 Hz; 1–2 mm; 2-s tempo. High intensity	NA	1–6	3	NA	12	aPWV faPWV baPWV
Ho et al., 2012 (69)	52	16	16	RT	non-exercise	AT: 30 min/day of treadmill walking; RT: machine-based resistance exercises, 4 × 8–12 repetitions at 10RM load; COMB: 15 min aerobic plus 15 min resistance exercise. All protocols were performed 5 days/week for 12 weeks.	AT: moderate intensity RT:10RM moderate intensity	8–12	4	5	30	12	AIx
	53	17	16	AT+RT				1	1	5	30	12	
Jamka et al., 2021 (70)	55	0/41	0/44	AT+RT	non-exercise	Endurance-strength training was performed 3 days/week for 3 months (∼60 min/session), including 20 min of strength exercises at 50%–60% 1RM followed by 25 min of cycling at 50%–70% HRmax, plus warm-up and cool-down.	RT:50–60%1RM AT:50%–70% HRmax moderate intensity	1	NA	3	60	12	cfPWV AIx
Olson et al., 2006 (71)	38	0/15	0/15	RT	non-exercise	RT, ≥2 days/week. machine/free-weight exercises targeting major muscle groups, 3 × 8–10 repetitions, after a brief aerobic warm-up and core exercises; supervised for 16 weeks, then self-directed.	moderate-intensity	8–12	3	≥2	NA	52	FMD
Croymans et al., 2014 (72)	21	28/0	8/0	RT	non-exercise	Supervised high-intensity RT was performed 3 days/week for 12 weeks (∼1 h/session), using a linear periodized programme progressing from 2 × 12–15RM to 3 × 6–8RM across two alternating whole-body routines performed as supersets.	100% 6–15RM High intensity	6–15	2–3	3	60	12	cfPWV
Climie et al., 2019 (73)	57	11/8	118	SRA	non-exercise	SRA condition: 5 h of sitting interrupted every 30 min by 3 min of simple bodyweight resistance activities, including half-squats, calf raises, and single knee raises with gluteal contractions.	bodyweight	10	3	1	30	1	FMD
Craighead et al., 2021 (74)	67	9/9	10/8	IMST	sham IMST	IMST was performed 6 days/week for 6 weeks using a POWERbreathe K3 device: 30 breaths/day, completed as 5 × 6 inspiratory efforts with 1-min rest between sets; training intensity progressed from 55% to 75% PIMAX.	IMST: 55→65→75% High intensity PIMAX; Sham: 15% PIMAX	30	5	6	5	6	FMD cfPWV β-stiffness index

RT, resistance training; AT, aerobic training; RET, resistance exercise training; SRA3 or 6, simple resistance activities; CRT, circuit resistance training; SHT, supervised isometric handgrip training; HBT, home-based isometric handgrip training; aortic PWV, aortic pulse wave velocity; PWV, pulse wave velocity; cfPWV, carotid–femoral pulse wave velocity; baPWV, brachial-ankle pulse wave velocity; pPWV, peripheral pulse wave velocity; cPWV, central pulse wave velocity; FMD, flow-mediated dilation; PVR, peripheral vascular resistance; BF, blood flow; AIx, augmentation index; IMST: inspiratory muscle strength training.

### 3.3 Results of risk of bias assessment

**Figure 2:**
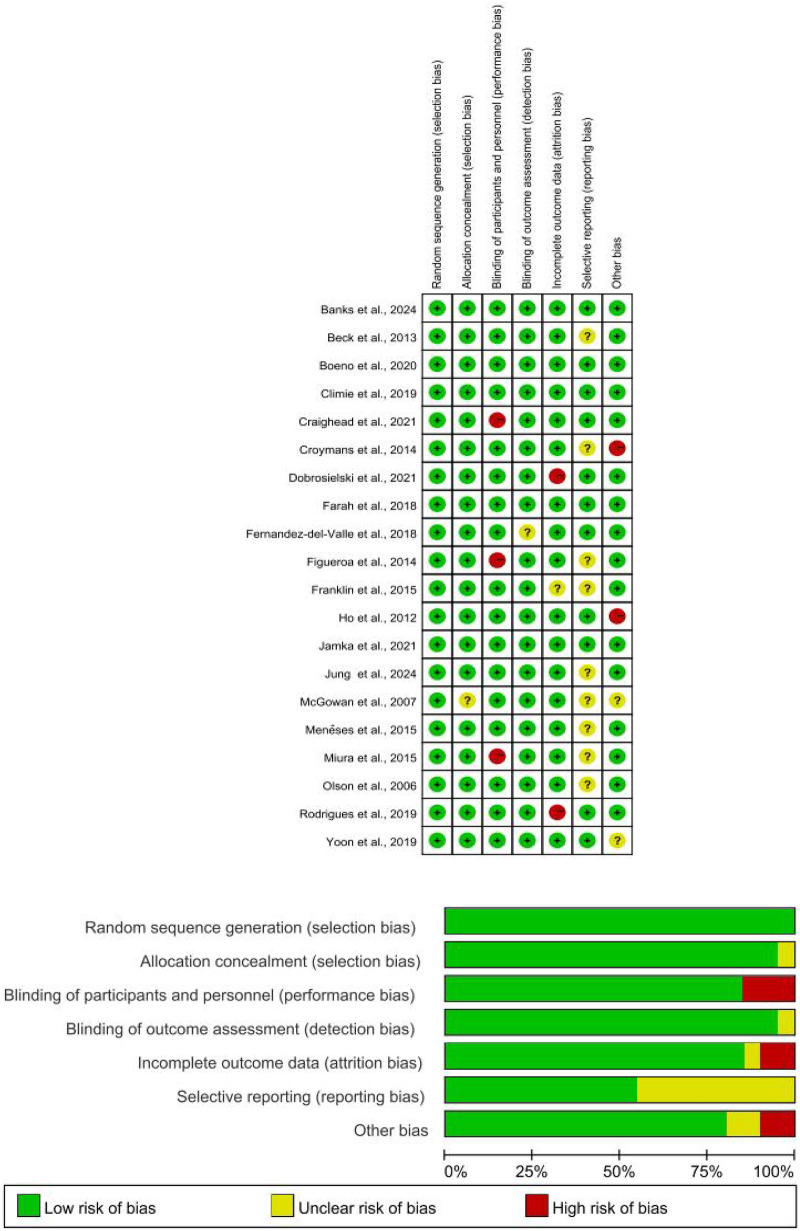
Risk of bias results.

The methodological quality of the included trials was systematically assessed using the Cochrane risk-of-bias tool, and a risk-of-bias summary figure was generated in Review Manager (RevMan 5.4.1). Overall, the principal methodological limitations of the included studies were concentrated in domains related to the randomization process, outcome measurement, and selective outcome reporting. These issues may have reduced the credibility of the findings and potentially influenced the effect estimates (Figure 2).

### 3.4 Results of meta-analysis

#### 3.4.1 Arterial stiffness

**Figure 3:**
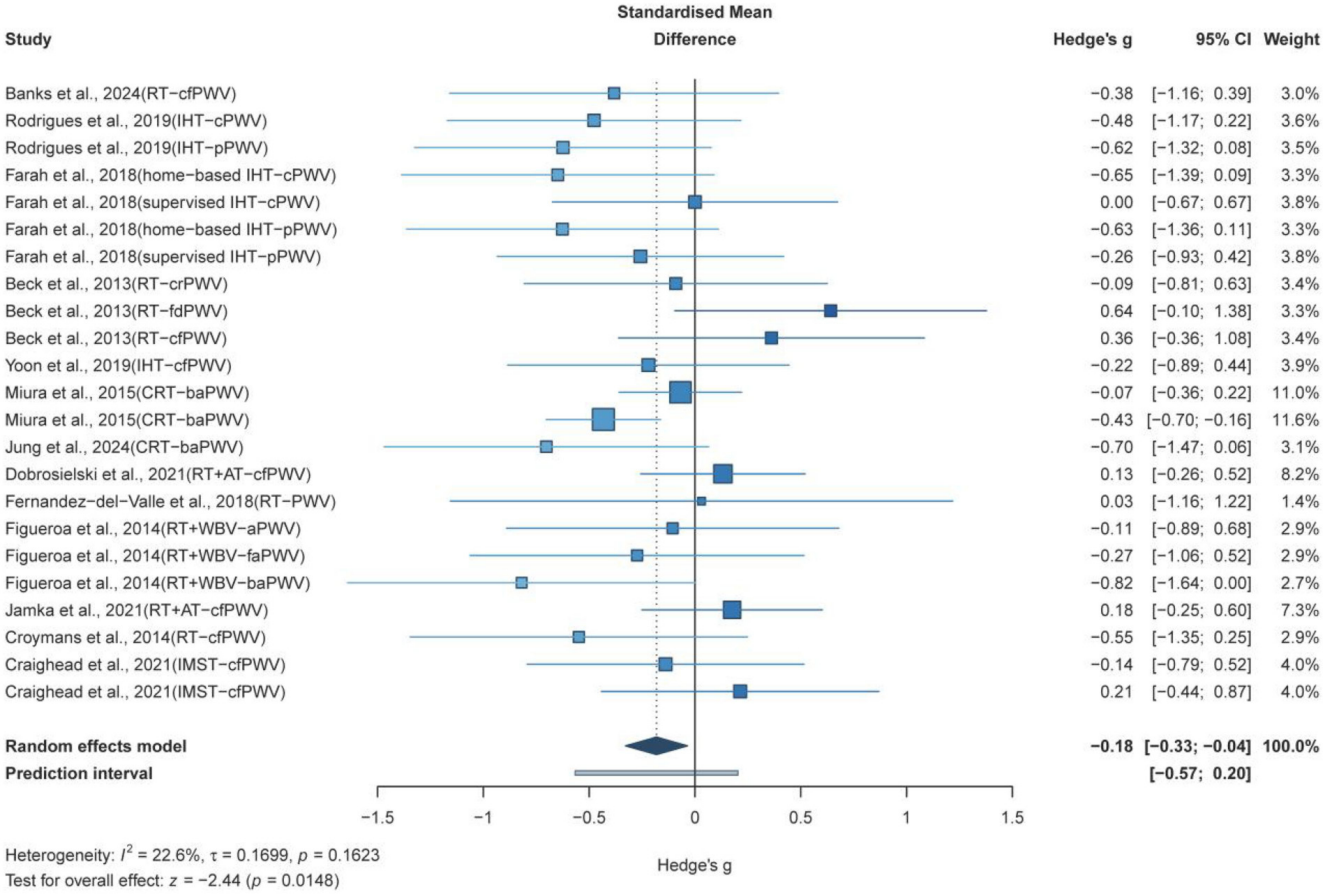
Forest plot for arterial stiffness. Forest plot of the effects of resistance training alone or resistance training combined with aerobic training on arterial stiffness in adults with hypertension or overweight/obesity-related vascular risk, compared with non-exercise/usual-care controls; 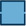, squares represent study-specific effect estimates, with square area proportional to study weight; 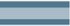, horizontal lines indicate 95% confidence intervals; 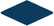, the diamond represents the pooled effect estimate, and its width indicates the 95% CI. Positive values favour exercise for arterial stiffness.

Meta-analysis of 13 studies (23 effect sizes; 1,154 participants) showed that, compared with non-exercise controls, resistance training-based interventions performed alone or combined with aerobic training produced a small but statistically significant reduction in arterial stiffness in adults with hypertension or overweight/obesity-related vascular risk (*g* = −0.18, 95% CI −0.33 to −0.04; *p* = 0.01; Figure 3). Repeated study references in Figure 3 represent distinct eligible effect sizes derived from different arterial stiffness indices, intervention arms, or independent comparisons, rather than duplicate inclusion of the same data.

However, sensitivity analyses suggested that this result was not fully robust after excluding non-traditional resistance-based modalities. When isometric handgrip training, whole-body vibration, brief intermittent resistance-type activity, and inspiratory muscle strength training were excluded, the pooled effect was attenuated and became non-significant (*g* = −0.08, 95% CI −0.30–0.14; *p* = 0.459). A stricter analysis retaining only conventional dynamic resistance training and combined aerobic–resistance training also showed a non-significant effect (*g* = 0.09, 95% CI −0.12–0.31; *p* = 0.391). Therefore, the arterial stiffness finding should be interpreted cautiously and may be partly influenced by non-traditional resistance-based modalities (Supplementary Figure 1).

Beyond intervention modality, subgroup analyses for arterial stiffness were also performed according to age category, sex composition, training intensity, repetitions, number of sets, weekly training frequency, session duration, and intervention duration, with full results presented in Table 2. Apparent between-subgroup differences were observed for sex composition, number of sets, and weekly training frequency (*p* < 0.05), whereas no significant between-subgroup differences were observed for age category, intervention modality, training intensity, repetitions, session duration, or intervention duration. Given the limited number of trials within several subgroups and the absence of significant associations in corresponding meta-regression analyses, these findings should be interpreted as exploratory patterns rather than definitive moderator effects.

**Table 2:** Subgroup analysis of meta-analysis results of arterial stiffness.

Moderator	Subgroup	K (N)	Hedge’s g	95% CI	P_d_	Q	I^2^ (%)	Power (%)	P_m_
Age									0.11
	Young Adult: 19–24	5 (137)	0.10	−0.33 to 0.53	0.65	5.4	26	21.73	
	Adults aged 25–44	1 (101)	0.13	−0.26 to 0.52	*n/a*	*n/a*	*n/a*	*n/a*	
	Middle Aged: 45–64	12 (482)	−0.24	−0.46 to −0.02	0.03	13.33	17.5	21.77	
	Aged: ≥65	7 (637)	−0.16	−0.38 to 0.06	0.15	9.61	37.6	35.98	
Sex									0.01
	Male	1 (36)	−0.55	−1.35 to 0.25	*n/a*	*n/a*	*n/a*	*n/a*	
	Female	6 (199)	−0.23	−0.61 to 0.14	0.21	6.96	28.2	24.89	
	Mixed-gender	16 (919)	−0.16	−0.33 to 0.00	0.05	20.6	27.2	36.67	
Intervention									0.7
	RT	21 (967)	−0.24	−0.39 to −0.10	0.001	22.21	10	17.92	
	RT+AT	2 (184)	0.15	−0.14 to 0.44	0.3	0.02	0	10	
Intensity									0.54
	Bodyweight	*n/a*	*n/a*	*n/a*	*n/a*	*n/a*	*n/a*	*n/a*	
	Low intensity	*n/a*	*n/a*	*n/a*	*n/a*	*n/a*	*n/a*	*n/a*	
	Moderate intensity	9 (340)	−0.28	−0.52 to −0.03	0.02	8.09	1.2	10.5	
	High intensity	9 (324)	−0.18	−0.44 to 0.08	0.18	8.89	10.1	14.98	
	To failure	3 (90)	0.30	−0.12 to 0.72	0.16	1.99	0	10	
Repetitions									0.47
	≤5	6 (248)	−0.19	−0.48 to 0.11	0.2	5.92	15.6	16.68	
	6–15	6 (239)	0.12	−0.14 to 0.38	0.3	5.41	7.7	12.91	
	>15	4 (472)	−0.18	−0.45 to 0.10	0.2	5.18	42.1	31.29	
Sets									0.0001
	≤3	10 (342)	−0.10	−0.39 to 0.18	0.48	13.46	33.2	36.84	
	4–6	13 (404)	−0.33	−0.53 to −0.14	0.0001	8.82	0	10	
	>6	2 (400)	−0.25	−0.61 to 0.10	0.1	3.25	69.3	44.4	
Frequency (t/wk)									0.02
	2	2 (400)	−0.25	−0.61 to 0.10	0.16	3.25	69.3	44.4	
	3	19 (682)	−0.19	−0.37 to −0.01	0.04	22.97	21.7	31.16	
	6	2 (72)	0.04	−0.43 to 0.50	0.87	0.55	0	10	
Time (min)									0.07
	≤20	7 (235)	−0.22	−0.47 to 0.04	0.1	4.62	0	10	
	21–59	3 (65)	−0.44	−0.94 to 0.55	0.07	1.07	0	10	
	≥60	9 (741)	−0.07	−0.31 to 0.17	0.5	17.87	55.2	70.12	
Duration (wk)									0.7
	3	1 (11)	0.03	−1.16 to 1.22	*n/a*	*n/a*	*n/a*	*n/a*	
	6	3 (174)	0.09	−0.20 to 0.39	0.5	0.65	0	10	
	8	3 (90)	0.30	−0.12 to 0.72	0.16	1.99	0	10	
	9	1 (26)	−0.38	−1.16 to 0.39	*n/a*	*n/a*	*n/a*	*n/a*	
	12	15 (853)	−0.30	−0.47 to −0.14	0.0004	14.84	5.7	13.40	

K (N) denotes the number of effect sizes included in the pooled estimate and the total number of participants contributing to that estimate. Negative values indicate improved arterial stiffness following resistance training alone or resistance training combined with aerobic training. P_d_, *p* value for the pooled effect estimate; Power, statistical power of the pooled subgroup estimate; P_m_, *p* value for between-subgroup differences; n/a, data unavailable.

Descriptive subgroup analyses for arterial stiffness suggested apparent between-subgroup differences according to sex composition, number of sets, and weekly training frequency. Mixed-sex samples, programmes involving 4–6 sets, and programmes performed three times per week showed more favourable descriptive estimates. However, these patterns should be interpreted cautiously because several strata contained few effect sizes and the corresponding meta-regression analyses did not identify significant linear associations. Therefore, these subgroup findings should not be interpreted as dose–response effects or optimal prescription thresholds.

The intervention-modality comparison also requires caution. Resistance training-based interventions performed alone showed a statistically significant pooled effect, whereas resistance training combined with aerobic training did not; however, the between-subgroup difference was not significant (*p* = 0.7). Thus, the available evidence is insufficient to conclude that resistance training-based interventions performed alone are superior to combined aerobic–resistance training. Overall, the subgroup findings for arterial stiffness should be considered exploratory and hypothesis-generating only.

**Figure 4:**
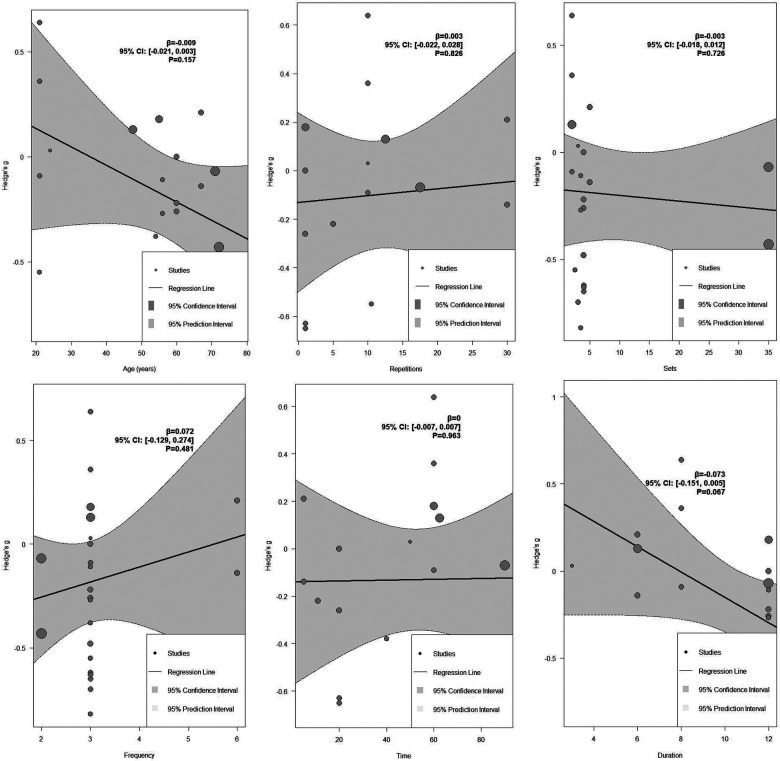
Meta-regression analysis based on meta-analysis results of arterial stiffness. Meta-regression bubble plot for age, repetitions, sets, frequency, time, duration. Bubble plots for meta-regression analyses of continuous variables. Each bubble denotes an individual effect size, and bubble size is proportional to its weight in the pooled analysis. β, represents the meta-regression coefficient, indicating the estimated change in *Hedge's g* for each 1-unit increase in the moderator. The black line represents the fitted regression line, the dark gray area the 95% confidence interval, and the light gray area the 95% prediction interval.

Table 2 and Figure 4 present different exploratory analyses: Table 2 compares predefined categorical subgroups, whereas Figure 4 tests linear associations between continuous moderators and effect sizes. Thus, apparent subgroup differences should not be interpreted as evidence of continuous dose–response effects. Meta-regression showed no significant linear associations between arterial stiffness effects and age, repetitions, sets, training frequency, session duration, or intervention duration (all *p* > 0.05; Figure 4).

#### 3.4.2 FMD

**Figure 5:**
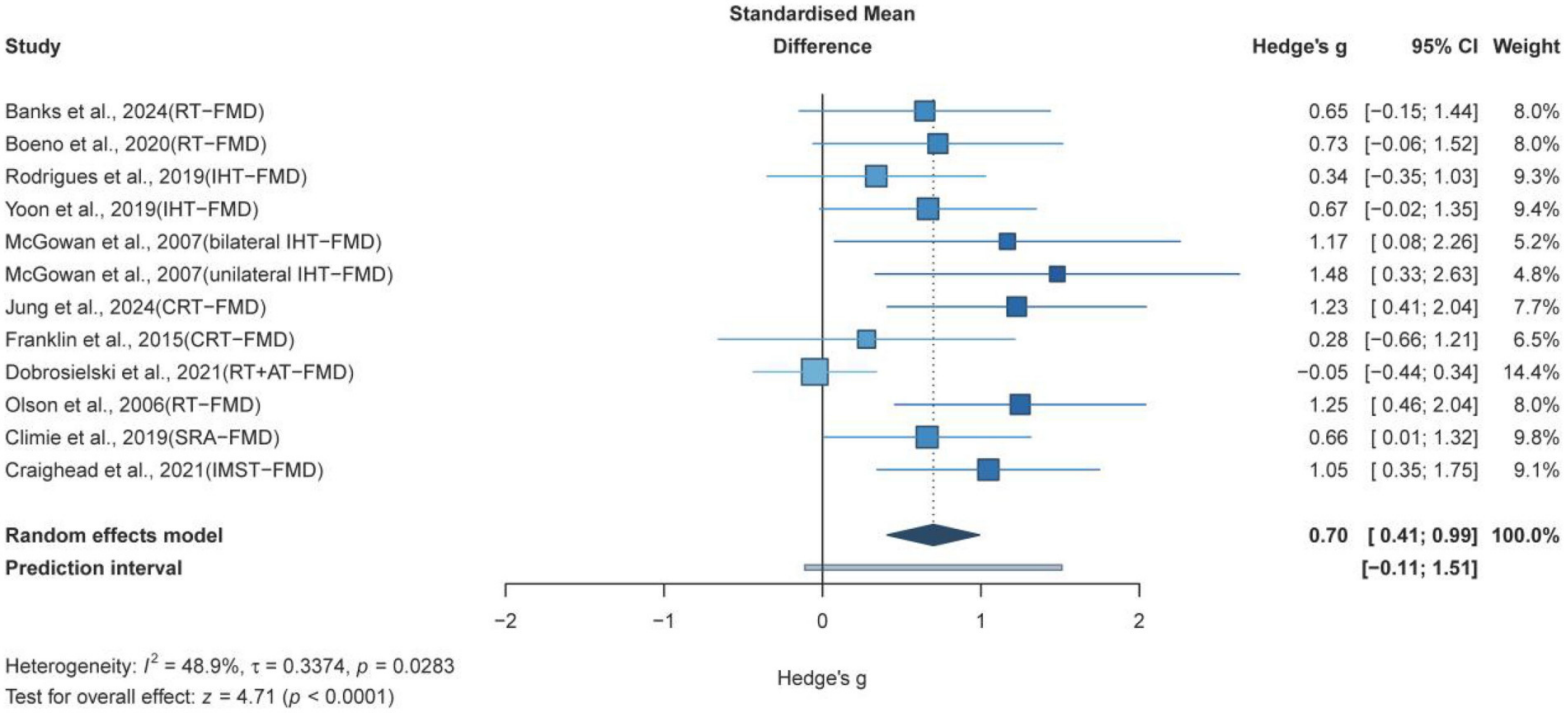
Forest plot for FMD. Forest plot of the effects of resistance training alone or resistance training combined with aerobic training on FMD in adults with hypertension or overweight/obesity-related vascular risk, compared with non-exercise/usual-care controls; 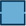, squares represent study-specific effect estimates, with square area proportional to study weight; 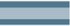, horizontal lines indicate 95% confidence intervals; 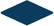, the diamond represents the pooled effect estimate, and its width indicates the 95% CI. Positive values favour exercise for FMD.

Meta-analysis of 11 studies (12 effect sizes; 389 participants) showed that, compared with non-exercise controls, resistance training-based interventions performed alone or combined with aerobic training produced a large and statistically significant improvement in endothelial function in adults with hypertension or overweight/obesity-related vascular risk (*g* = 0.70, *95% CI* 0.41–0.99; *p* < 0.0001; Figure 5). Repeated study references in Figure 5 denote distinct eligible effect sizes derived from different intervention arms or independent comparisons, rather than duplicate inclusion of the same data.

Sensitivity analyses were conducted to examine whether the FMD finding was driven by non-traditional resistance-based modalities. After excluding isometric handgrip training, brief intermittent resistance-type activity, and inspiratory muscle strength training, while retaining conventional dynamic resistance training, circuit resistance training, and combined aerobic–resistance training, the pooled effect remained statistically significant (*g* = 0.64, *95% CI* 0.14–1.14; *p* = 0.012). In a stricter sensitivity analysis retaining only conventional dynamic resistance training and combined aerobic–resistance training, the effect remained positive but was no longer statistically significant (*g* = 0.58, *95% CI* −0.04–1.20; *p* = 0.066). These findings suggest that the observed improvement in FMD was not solely driven by non-traditional resistance-based modalities, although the evidence became less precise when restricted to conventional RT and RT+AT only (Supplementary Figure 2).

Beyond intervention modality, subgroup analyses for FMD were also performed according to age category, sex composition, training intensity, repetitions, number of sets, weekly training frequency, session duration, and intervention duration, with full results presented in Table 3. Apparent between-subgroup differences were observed for age category, sex composition, training intensity, repetitions, number of sets, weekly training frequency, session duration, and intervention duration, whereas the between-subgroup difference for intervention modality was not statistically significant. Because several subgroups contained few effect sizes and corresponding meta-regression analyses did not confirm significant linear associations, these findings should be interpreted cautiously as hypothesis-generating patterns rather than definitive evidence of moderator effects.

**Table 3:** Subgroup analysis of meta-analysis results of FMD.

Moderator	Subgroup	K (N)	Hedge's g	95% CI	P_d_	Q	I^2^ (%)	Power (%)	P_m_
Age									0.0002
	Young Adult: 19–24	1 (18)	0.28	−0.66 to 1.21	*n/a*	*n/a*	*n/a*	*n/a*	
	Adults aged 25–44	3 (150)	0.45	−0.34 to 1.23	0.2	8.32	76	72.32	
	Middle Aged: 45–64	7 (258)	0.56	0.18 to 0.94	0.004	12.1	50.4	53.9	
	Aged: ≥65	4 (201)	0.66	0.06 to 1.26	0.03	12.89	76.7	84.34	
Sex									0.0001
	Male	*n/a*	*n/a*	*n/a*	*n/a*	*n/a*	*n/a*	*n/a*	
	Female	3 (76)	0.96	0.38 to 1.54	0.001	2.94	32.1	21.12	
	Mixed-gender	9 (329)	0.62	0.30 to 0.95	0.0002	15.35	47.9	56.83	
Intervention									0.35
	RT	11 (303)	0.81	0.57 to 1.05	0.001	7.94	0	10	
	RT+AT	1 (101)	−0.05	−0.44 to 0.34	*n/a*	*n/a*	*n/a*	*n/a*	
Intensity									0.0001
	Bodyweight	1 (38)	0.66	0.01 to 1.32	*n/a*	*n/a*	*n/a*	*n/a*	
	Low intensity	*n/a*	*n/a*	*n/a*	*n/a*	*n/a*	*n/a*	*n/a*	
	Moderate intensity	7 (174)	0.74	0.43 to 1.06	0.0001	6.1	1.7	10.65	
	High intensity	4 (193)	0.67	0.06 to 1.29	0.03	12.97	76.9	84.66	
	To failure	*n/a*	*n/a*	*n/a*	*n/a*	*n/a*	*n/a*	*n/a*	
Repetitions									0.001
	≤5	3 (67)	0.94	0.43 to 1.46	0.0003	1.63	0	10	
	6–15	5 (215)	0.52	0.04 to 0.99	0.03	10.76	62.8	64.82	
	>15	2 (63)	0.91	0.38 to 1.43	0.0007	0.35	0	10	
Sets									0.0001
	≤3	6 (243)	0.63	0.17 to 1.08	0.006	14.7	66	76.92	
	>3	6 (164)	0.88	0.54 to 1.22	0.0001	4.97	0	10	
Frequency (t/wk)									0.0001
	1	1 (38)	0.66	0.01 to 1.32	*n/a*	*n/a*	*n/a*	*n/a*	
	2	2 (48)	0.80	−0.15 to 1.74	0.09	2.4	58.4	32.48	
	3	9 (313)	0.72	0.35 to 1.09	0.0001	19.19	58.3	75.81	
	6	2 (66)	1.14	0.61 to 1.66	0.0001	0.13	0	10	
Time (min)									0.0001
	≤20	4 (103)	0.98	0.56 to 1.40	0.0001	1.69	0	10	
	21–59	4 (119)	0.79	0.42 to 1.17	0.0001	1.39	0	10	
	≥60	3 (148)	0.43	−0.34 to 1.20	0.27	7.65	73.9	67.77	
Duration (wk)									0.0001
	1	1 (38)	0.66	0.01 to 1.32	*n/a*	*n/a*	*n/a*	*n/a*	
	6	2 (138)	0.46	−0.61 to 1.53	0.4	7.16	86.1	79.92	
	8	3 (50)	0.92	0.17 to 1.66	0.01	2.9	31.2	20.66	
	9	1 (26)	0.65	−0.15 to 1.44	*n/a*	*n/a*	*n/a*	*n/a*	
	12	4 (123)	0.70	0.33 to 1.07	0.0002	2.65	0	10	
	52	1 (30)	1.25	0.46 to 2.04	*n/a*	*n/a*	*n/a*	*n/a*	

K (N) denotes the number of effect sizes included in the pooled estimate and the total number of participants contributing to that estimate. Positive values indicate improved FMD following resistance training alone or resistance training combined with aerobic training. P_d_, *p* value for the pooled effect estimate; Power, statistical power of the pooled subgroup estimate; P_m_, *p* value for between-subgroup differences; n/a, data unavailable.

Descriptive subgroup analyses for FMD suggested apparent between-subgroup differences across several participant and intervention-related categories, including age category, sex composition, training intensity, repetitions, number of sets, weekly training frequency, session duration, and intervention duration. However, these findings should be interpreted cautiously because several subgroups contained few effect sizes and the corresponding meta-regression analyses did not identify significant linear associations. Therefore, these subgroup patterns should not be interpreted as dose–response effects, confirmed moderator effects, or optimal exercise-prescription thresholds.

Although some categories, such as older adults, female samples, moderate-intensity training, lower repetition ranges, higher set numbers, six sessions per week, 21–59 min sessions, and 8-week interventions, showed larger descriptive estimates, these patterns remain exploratory and may reflect study-level differences rather than true causal moderation. The intervention-modality comparison also requires caution: resistance training-based interventions performed alone showed a statistically significant pooled effect, whereas RT+AT did not; however, the between-subgroup difference was not significant (*p* = 0.35), and only one RT+AT effect size was available. Thus, the current evidence is insufficient to determine whether resistance training-based interventions performed alone differ from combined aerobic–resistance training in their effects on FMD.

**Figure 6:**
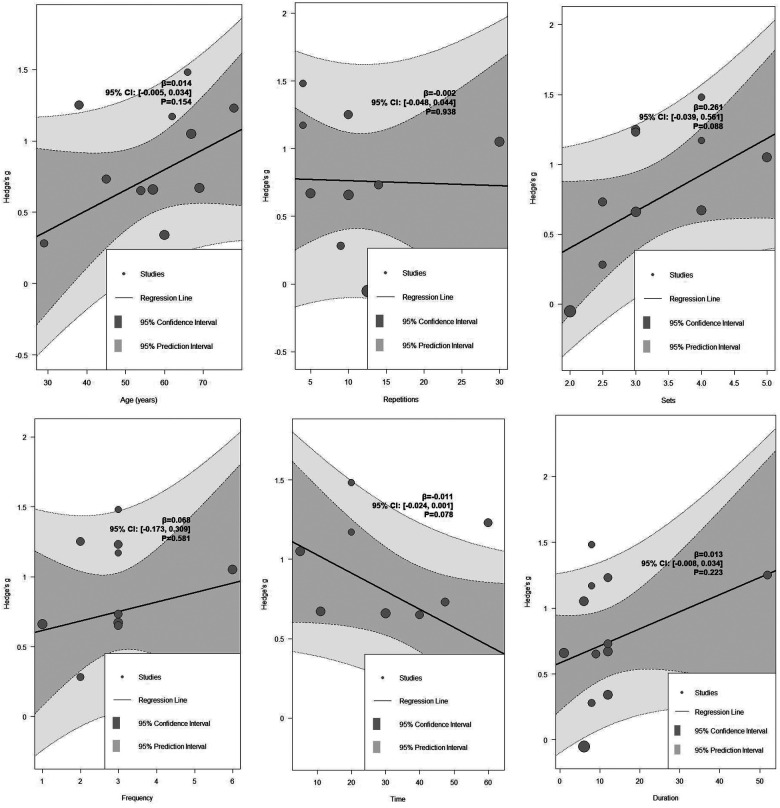
Meta-regression analysis based on meta-analysis results of FMD. Meta-regression bubble plot for age, repetitions, sets, frequency, time, duration. Bubble plots for meta-regression analyses of continuous variables. Each bubble denotes an individual effect size, and bubble size is proportional to its weight in the pooled analysis. β, represents the meta-regression coefficient, indicating the estimated change in *Hedge's g* for each 1-unit increase in the moderator. The black line represents the fitted regression line, the dark gray area the 95% confidence interval, and the light gray area the 95% prediction interval.

Table 3 and Figure 6 should be interpreted as distinct exploratory analyses: the Table 3 compares predefined categorical strata, whereas Figure 6 tests continuous linear associations between moderators and FMD effect sizes. Thus, apparent subgroup differences do not necessarily imply dose–response effects. Meta-regression showed no significant linear associations between FMD effects and age, repetitions, sets, training frequency, session duration, or intervention duration (all *p* > 0.05; Figure 6).

#### 3.4.3 Wave reflection indices

**Figure 7:**
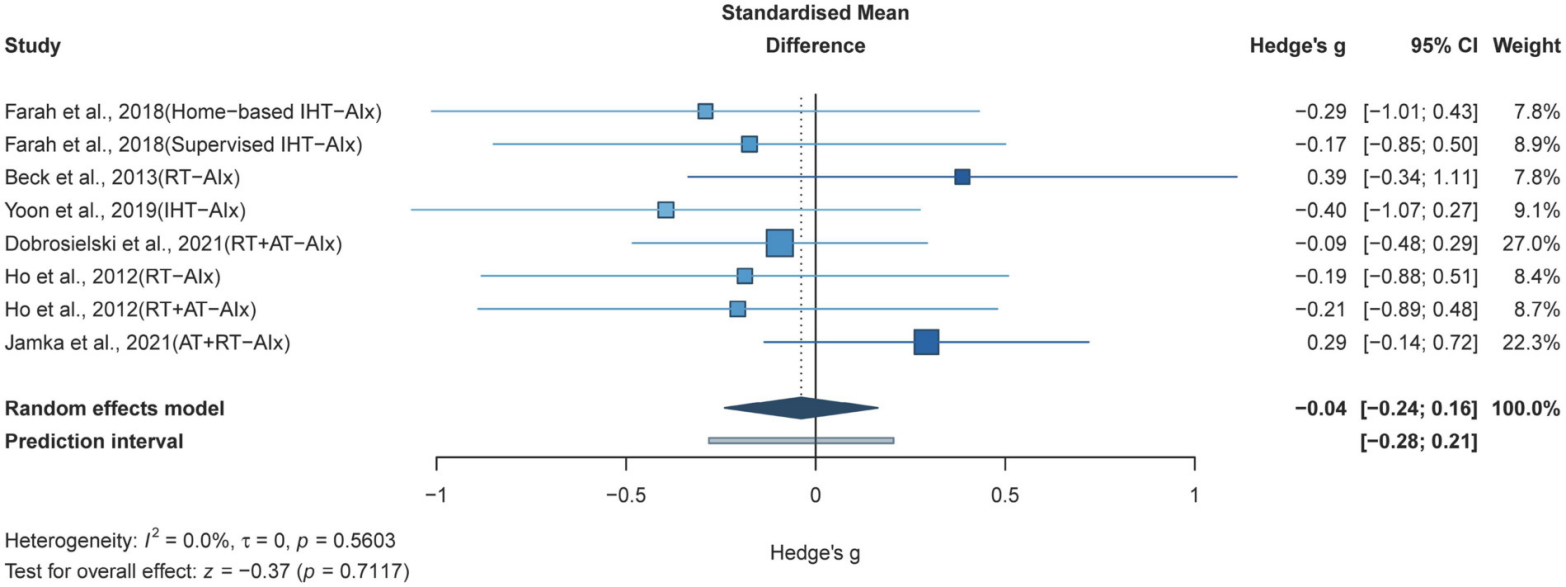
Forest plot for wave reflection indices. Forest plot of the effects of resistance training alone or resistance training combined with aerobic training on wave reflection indices in adults with hypertension or overweight/obesity-related vascular risk, compared with non-exercise/usual-care controls; 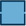, squares represent study-specific effect estimates, with square area proportional to study weight; 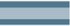, horizontal lines indicate 95% confidence intervals; 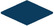, the diamond represents the pooled effect estimate, and its width indicates the 95% CI. Positive values favour exercise for wave reflection indices.

Meta-analysis of six studies (eight effect sizes; 389 participants) showed that, compared with non-exercise controls, resistance training-based interventions performed alone or combined with aerobic training did not significantly improve wave reflection indices in adults with hypertension or overweight/obesity-related vascular risk (*g* = −0.04, 95% CI −0.24–0.16; *p* = 0.71; Figure 7). Repeated study references in Figure 7 denote distinct eligible effect sizes derived from different intervention arms, wave reflection indices, or independent comparisons, rather than duplicate inclusion of the same data.

Sensitivity analysis was conducted to examine whether this null finding was influenced by non-traditional resistance-based modalities. In the AIx dataset, the only non-traditional modality was isometric handgrip training. After excluding isometric handgrip training and retaining conventional dynamic resistance training and combined aerobic–resistance training, the pooled effect remained non-significant (*g* = 0.05, 95% CI −0.19–0.28; *p* = 0.683). This was consistent with the primary analysis, suggesting that the null finding for wave reflection indices was not driven by isometric handgrip training (Supplementary Figure 3).

#### 3.4.4 Peripheral hemodynamics

Only one acute study reported peripheral haemodynamic outcomes; therefore, no meta-analysis was performed and the findings are presented narratively. In that study, Menêses et al. (56) examined immediate post-exercise haemodynamic responses in hypertensive women following two combined exercise sequences: endurance exercise followed by resistance exercise and resistance exercise followed by endurance exercise. Both exercise sequences attenuated the increase in peripheral vascular resistance and the reduction in cardiac output observed under the non-exercise control condition. Stroke volume decreased and heart rate increased after both exercise sessions, with no significant between-sequence differences.

Because this evidence was derived from a single acute study, these findings should be interpreted only as short-term post-exercise haemodynamic responses under specific experimental conditions. They do not provide evidence for sustained peripheral haemodynamic adaptation to chronic resistance training-based interventions.

### 3.5 Publication bias

**Figure 8:**
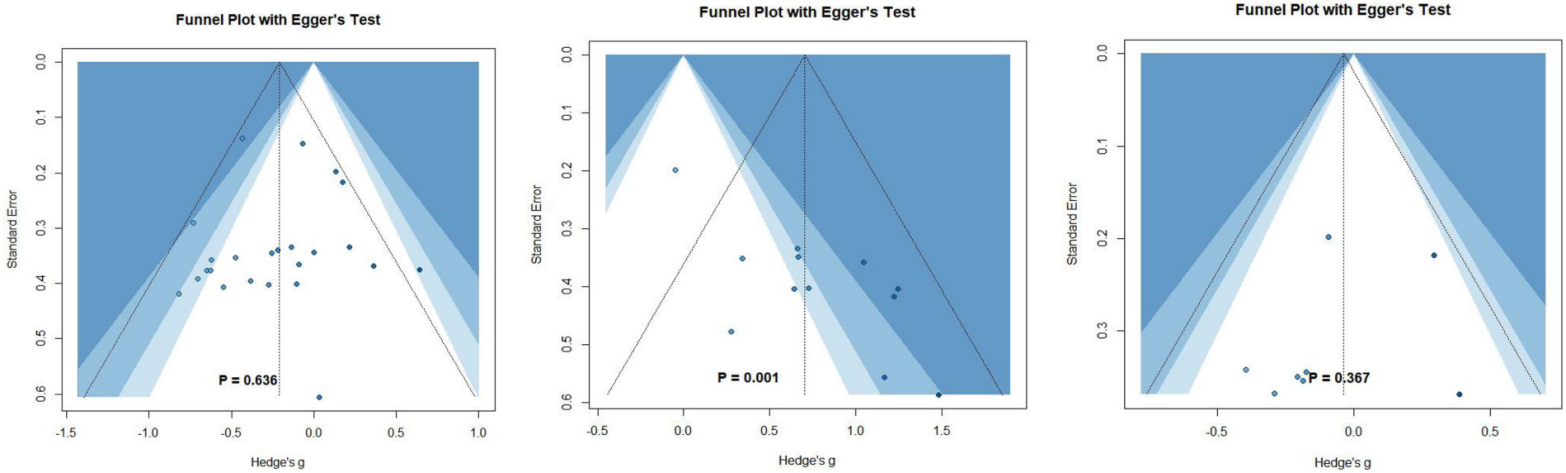
Publication bias. Funnel plots for arterial stiffness, FMD, wave reflection indices. Each dot represents an individual study effect size. The *x*-axis indicates *Hedge's g*, and the *y*-axis indicates the standard error. The vertical line represents the pooled effect estimate, and the diagonal lines indicate the pseudo 95% confidence limits.

According to Egger's test, the pooled results for arterial stiffness and AIx showed no clear evidence of publication bias (*p* > 0.05), whereas the findings for FMD suggested a potential risk of publication bias or small-study effects (*p* < 0.05). Therefore, the magnitude of the pooled FMD effect should be interpreted cautiously and may have been overestimated. The corresponding funnel plots are presented in Figure 8.

### 3.6 GRADE level

**Figure 9:**
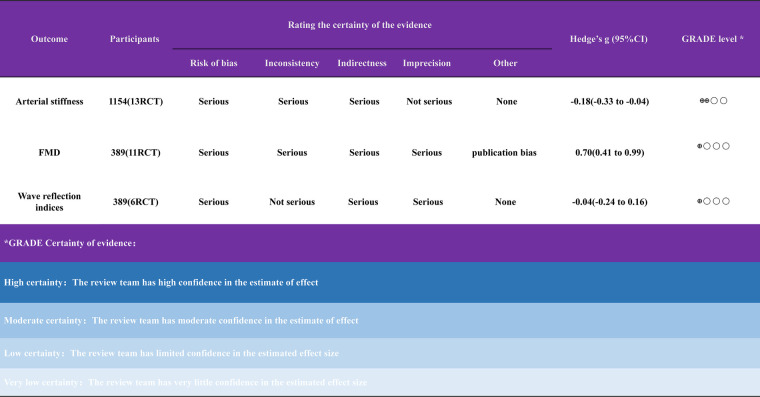
GRADE level of evidence for this study's findings. Randomised controlled trials start as high-certainty evidence. Certainty was downgraded by one level for each domain judged as serious and by two levels for any domain judged as very serious.

The evidence certainty (quality) ratings for the outcomes in this systematic review are presented in Figure 9.

## 4 Discussion

### 4.1 Effects of resistance training alone or combined with aerobic training on arterial stiffness in adults with hypertension or overweight/obesity-related vascular risk

The present analysis showed that resistance training alone or combined with aerobic training produced a small but statistically significant reduction in arterial stiffness in adults with hypertension or overweight/obesity-related vascular risk. This finding is clinically relevant because arterial stiffening is a key manifestation of hypertension-related vascular injury and is closely associated with target-organ damage and cardiovascular risk (75). Thus, resistance training-based interventions may confer vascular benefits beyond conventional blood pressure control.

This effect is physiologically plausible in this high-risk population. Overweight/obesity with hypertension is characterized by adipose tissue dysfunction, inflammation, oxidative stress, sympathetic overactivity, impaired nitric oxide bioavailability, and increased haemodynamic load. Resistance training-based exercise may therefore reduce arterial stiffness through combined effects on insulin sensitivity, adiposity, blood pressure load, peripheral vascular tone, and endothelial regulation. Evidence linking exercise-induced irisin and nitric oxide production with lower arterial stiffness, as well as evidence connecting adipokine imbalance with vascular inflammation and remodelling, supports this interpretation (75–77).

Compared with previous evidence, the present findings suggest that the effect of resistance training on arterial stiffness may depend on population risk profile and training configuration. Recent HIIT-focused evidence provides useful but not directly interchangeable context. Jiao et al. reported that HIIT improved several haemostatic and vascular stiffness-related outcomes, including cfPWV, AIx, AIx@75HR, and FMD, although HIIT was not consistently superior to moderate-intensity continuous training across all parameters (78). This is broadly consistent with the present finding that exercise-related vascular adaptations may be outcome-specific. However, comparability is limited because HIIT mainly provides an aerobic interval stimulus characterized by repeated cardiorespiratory and shear-stress exposure, whereas resistance training-based interventions involve muscular loading, intermittent vascular compression, and pressure-load responses (79, 80). Moreover, the HIIT review included broader populations and haemostatic outcomes, whereas the present review focused on resistance training-based interventions in adults with hypertension or overweight/obesity-related vascular risk. Thus, HIIT evidence supports the broader exercise–vascular health framework, but it cannot be used as direct evidence for resistance training effects (78). Reviews in general adult or healthy populations have reported inconsistent effects of resistance training on PWV, with some high-intensity or upper-limb-dominant protocols associated with increased arterial stiffness or wave reflection (81–84). By contrast, in adults with hypertension or overweight/obesity-related vascular risk, greater baseline vascular burden may provide greater scope for improvement, particularly when training reduces metabolic stress, blood pressure load, and endothelial dysfunction.

Findings from cardiovascular or metabolic risk populations are more consistent with the direction of the present result. Resistance training has been reported not to worsen arterial stiffness in individuals at increased cardiovascular risk, and progressive resistance training without excessive volume progression does not appear to increase central or peripheral arterial stiffness (85, 86). However, short-term resistance training has also been associated with increased PWV in untreated prehypertension or stage 1 hypertension (87). These discrepancies suggest that blood pressure and arterial stiffness may not change in parallel: blood pressure may respond rapidly to neurohumoral regulation, whereas arterial stiffness may require longer-term vascular wall adaptation. This is consistent with evidence that improvements in arterial stiffness in prehypertensive or hypertensive populations are more likely after longer interventions or when systolic blood pressure reductions are more pronounced (88).

Subgroup analyses in the present study suggested that sex composition, number of sets, and training frequency were associated with apparent differences in arterial stiffness effect estimates, with mixed-sex samples and programmes involving 4–6 sets per session and three sessions per week showing more favorable patterns. However, these findings warrant cautious interpretation and should not be presented as confirmed moderator effects. The number of studies within several subgroups was limited, and meta-regression did not confirm significant linear associations between effect size and age, number of repetitions, number of sets, training frequency, session duration, or intervention length. Accordingly, these subgroup results are better regarded as hypothesis-generating observations than as quantitatively generalizable training thresholds. The apparent patterns observed for mixed-sex samples, programmes involving 4–6 sets, and programmes performed three times per week may reflect differences in sample composition, intervention design, baseline vascular risk, or measurement variability rather than true independent prescription effects. Similarly, the non-significant between-subgroup difference between resistance training-based interventions performed alone and those combined with aerobic training indicates that the current evidence is insufficient to determine whether one intervention model is superior to the other. This interpretation is consistent with previous evidence showing that the vascular effects of resistance training may vary according to intensity, loading pattern, age, baseline blood pressure status, and sex-related vascular characteristics (89, 90).

The modality-specific findings also require caution. Although the resistance training alone subgroup reached statistical significance whereas the RT+AT subgroup did not, the between-subgroup difference was not significant. Therefore, the present evidence does not support the conclusion that RT is superior to RT+AT, nor does it indicate that RT+AT is ineffective. Previous evidence has similarly shown no consistent advantage of combined training for PWV in broader populations, and exercise effects on PWV in individuals with overweight/obesity appear less consistent for resistance and combined training than for aerobic training (81, 91).

From a practical perspective, structured resistance training may represent a feasible complementary strategy for vascular risk management in adults with hypertension or overweight/obesity-related vascular risk, particularly for individuals who have difficulty adhering to prolonged aerobic training because of time constraints, joint burden, low muscle mass, functional limitations, or poor exercise adherence (92–94). Nevertheless, the present findings should be interpreted within several limitations. Different arterial stiffness indices were pooled, relatively few studies evaluated RT+AT, and subgroup findings were not supported by significant continuous meta-regression results. Future trials should use standardized arterial stiffness measures, particularly cfPWV, directly compare RT with RT+AT under time- and energy-matched conditions, and report relevant mechanistic markers such as blood pressure, adiposity, inflammation, and nitric oxide-related biomarkers.

### 4.2 Effects of resistance training alone or combined with aerobic training on FMD in adults with hypertension or overweight/obesity-related vascular risk

The present analysis showed that resistance training alone or combined with aerobic training significantly improved FMD in adults with hypertension or overweight/obesity-related vascular risk, with a larger effect than that observed for arterial stiffness. This suggests that resistance training-based interventions may act more clearly on endothelial dysfunction than on structural vascular stiffening in this high-risk population.

This finding is physiologically plausible. FMD reflects endothelium-dependent vasodilatory responsiveness to shear stress and is largely mediated by the nitric oxide (NO) pathway. Although resistance training differs haemodynamically from continuous aerobic exercise, repeated increases in blood flow, arterial diameter fluctuation, and shear stress during exercise may stimulate endothelial adaptation and improve vasodilatory function (95). In adults with hypertension or overweight/obesity-related vascular risk, who are commonly characterized by inflammation, oxidative stress, sympathetic activation, and impaired NO bioavailability, FMD may therefore be particularly responsive to exercise-induced vascular stimuli (96, 97).

The present findings are broadly consistent with previous evidence that exercise training improves endothelial function across different populations and modalities (98, 99). Improvements in FMD have been reported in metabolic syndrome, type 2 diabetes, overweight/obesity, and middle-aged or older adults (100–104). More directly relevant to the present review, resistance training-specific evidence suggests that RT can improve endothelial function, although responses may vary by training intensity, population characteristics, and baseline cardiovascular risk (30, 41, 105). Evidence from aerobic and interval-based exercise also indicates that training intensity may influence FMD responses in obesity-related or metabolic-risk populations (106–108). However, these findings should not be interpreted as evidence that higher-intensity training is universally superior.

Compared with previous reviews in hypertensive populations, the present study provides a more specific synthesis focused on adults with hypertension or overweight/obesity-related vascular risk. Pedralli et al. reported that exercise training improved FMD in individuals with hypertension, although the evidence was mainly derived from aerobic training studies and was accompanied by substantial heterogeneity (109). Waclawovsky et al. similarly noted that moderate-intensity continuous aerobic training has the strongest evidence base in prehypertensive and hypertensive adults, whereas resistance training has been viewed more as a complementary strategy (110). The present findings extend this literature by suggesting that resistance training-based interventions, including isometric handgrip training, circuit resistance training, and brief intermittent resistance-type activity, can also produce meaningful endothelial benefits in a higher-risk metabolic–vascular phenotype.

Evidence from adults with overweight/obesity further supports this interpretation. Cortes et al. reported that exercise training improved FMD overall, although the resistance training subgroup did not reach statistical significance, likely because of the small number of available resistance training studies (103). The clearer positive effect observed in the present analysis may reflect the more concentrated vascular risk profile of adults with coexisting hypertension and overweight/obesity-related vascular risk, in whom baseline endothelial dysfunction may be greater and the potential for improvement correspondingly larger.

The exploratory subgroup findings for FMD require cautious interpretation. Although categorical subgroup analyses suggested apparent variation across participant and intervention-related characteristics, the corresponding meta-regression analyses did not identify significant linear associations. Thus, these findings should not be interpreted as evidence of dose–response effects, confirmed moderator effects, or optimal exercise-prescription thresholds. These apparent subgroup patterns may reflect sparse data, categorical cut-points, and uneven distributions of participant characteristics, baseline vascular risk, intervention modality, or FMD assessment protocols across subgroups. Accordingly, they should be regarded as descriptive and hypothesis-generating only. They may help inform future adequately powered trials, but they do not justify quantitative prescription recommendations in the present evidence base (111).

From a clinical perspective, the improvement in FMD may be meaningful because endothelial function has prognostic relevance. Previous evidence suggests that each 1% increase in endothelial function or FMD may be associated with an approximately 12%–13% lower risk of future cardiovascular events (109, 110). Although the present analysis used standardized effect sizes and therefore cannot be directly converted into absolute percentage changes in FMD, the direction and magnitude of the pooled effect support the potential translational value of resistance training-based interventions for endothelial health in adults with hypertension or overweight/obesity-related vascular risk.

Several limitations should be considered. The FMD analysis was based on 11 studies, 12 effect sizes, and 389 participants, indicating a relatively limited evidence base. Publication bias was suggested for FMD, so the pooled effect may have been overestimated. In addition, FMD is sensitive to methodological factors such as cuff position, baseline arterial diameter, image analysis, and pre-test standardization. Future studies should use standardized FMD protocols, directly compare RT with RT+AT under time- and energy-matched conditions, and report mechanistic markers such as NO bioavailability, oxidative stress, inflammation, body composition, and blood pressure to clarify whether endothelial improvements are driven primarily by local shear-stress stimulation or broader metabolic and haemodynamic adaptation.

Overall, the subgroup analyses in the present review should be understood as exploratory tools for identifying possible sources of heterogeneity rather than as a basis for clinical prescription. The absence of significant continuous meta-regression findings means that no firm conclusions can be drawn regarding optimal training intensity, volume, frequency, session duration, or intervention duration.

### 4.3 Effects of resistance training alone or combined with aerobic training on wave reflection indices in adults with hypertension or overweight/obesity-related vascular risk

The present analysis found no consistent improvement in wave reflection indices following resistance training alone or resistance training combined with aerobic training in adults with hypertension or overweight/obesity-related vascular risk. This pattern contrasts with the modest reduction observed for arterial stiffness and the clearer improvement observed for FMD, suggesting that the vascular effects of resistance training-based interventions may be outcome-specific rather than uniform across all vascular domains.

This null finding is physiologically plausible because wave reflection indices, particularly the augmentation index (AIx), represent a more composite vascular phenotype than PWV or FMD. AIx is influenced not only by arterial stiffness, but also by heart rate, height, age, systolic blood pressure, left ventricular ejection duration, the timing of reflected wave return, and the distribution of peripheral reflection sites (112–115). Therefore, even when resistance training-based interventions improve endothelial function or reduce arterial stiffness, these changes may not translate directly into a measurable reduction in AIx. Small changes in heart rate, autonomic tone, or peripheral vascular resistance may also offset or obscure potential improvements in wave reflection (116, 117).

The present finding is broadly consistent with previous evidence showing that AIx and related wave reflection measures respond less consistently to exercise training than PWV or FMD. Although some individual studies have reported reductions in AIx after exercise-based interventions (118, 119), meta-analytic evidence in high-risk populations has shown that exercise may improve PWV and blood pressure without producing a parallel reduction in AIx (120). Evidence from acute exercise studies also indicates that wave reflection responses vary according to exercise modality, timing of post-exercise assessment, and autonomic state (121, 122). These findings support the interpretation that wave reflection indices are sensitive to both vascular and non-vascular determinants, and may therefore show greater heterogeneity than arterial stiffness or endothelial function outcomes.

Accordingly, the absence of a significant effect on wave reflection indices should not be interpreted as evidence that resistance training-based interventions lack vascular benefit. Rather, it suggests that their benefits in this population may be more clearly expressed through improved endothelial function and reduced arterial stiffness than through changes in central pressure wave morphology. This conclusion should be interpreted cautiously because only six studies and eight effect sizes were available for wave reflection outcomes. Future trials should standardize the assessment of AIx or AIx@75, report heart rate and central blood pressure concurrently, and examine whether longer or more targeted resistance training-based interventions can produce reproducible changes in wave reflection burden (123, 124).

### 4.4 Effects of resistance training alone or combined with aerobic training on peripheral hemodynamics in adults with hypertension or overweight/obesity-related vascular risk

The evidence for peripheral haemodynamic outcomes was extremely limited. Unlike arterial stiffness, FMD, and wave reflection indices, this outcome was informed by only one acute study, and no meta-analysis could be performed. Therefore, no reliable conclusion can be drawn regarding whether resistance training-based interventions produce stable or sustained changes in peripheral blood flow, peripheral vascular resistance, systemic vascular resistance, cardiac output, or stroke volume (125, 126).

The single eligible study by Menêses et al. (56), examined immediate post-exercise haemodynamic responses in hypertensive women following two combined exercise sequences: endurance exercise followed by resistance exercise and resistance exercise followed by endurance exercise. The study suggested that both exercise sequences attenuated the post-exercise increase in peripheral vascular resistance observed under the control condition and helped maintain cardiac output. However, these findings reflect acute recovery-phase haemodynamic responses after a single exercise session rather than chronic vascular adaptation to repeated training (127, 128).

Accordingly, the present review should not be interpreted as providing evidence that resistance training alone or combined aerobic–resistance training improves peripheral haemodynamics over the long term. The available evidence only indicates that acute combined exercise may transiently influence peripheral vascular resistance under specific experimental conditions. Its generalizability is limited by the single-study evidence base, the acute design, the specific hypertensive female sample, the combined-exercise format, and the recovery-phase measurement conditions. Future randomized trials should evaluate long-term peripheral haemodynamic adaptations using standardized measures of peripheral blood flow, peripheral vascular resistance, systemic vascular resistance, cardiac output, and stroke volume.

## 5 Conclusion

This systematic review and meta-analysis suggests that resistance training-based interventions may improve selected vascular function outcomes in adults with hypertension or overweight/obesity-related vascular risk. The most consistent positive signal was observed for endothelial function, as reflected by improved FMD; however, this finding should be interpreted cautiously because potential publication bias or small-study effects were detected, the evidence base was limited, and the pooled effect may have been overestimated. Arterial stiffness showed a small overall reduction, but this finding was attenuated and became non-significant in sensitivity analyses excluding non-traditional resistance-based modalities. No consistent benefit was observed for wave reflection indices, and evidence for peripheral haemodynamic outcomes remains insufficient.

The current evidence does not support the conclusion that resistance training-based interventions performed alone are superior to resistance training combined with aerobic training, because relatively few studies directly evaluated combined protocols and between-subgroup differences were not statistically significant. Likewise, subgroup patterns related to participant or prescription characteristics should be interpreted as exploratory and hypothesis-generating only, rather than as evidence of optimal training prescriptions. Given the low to very low certainty of evidence, methodological limitations, intervention heterogeneity, and sparse data for several outcomes, these findings should be interpreted cautiously. Larger, well-designed randomized controlled trials are needed to confirm the effects on FMD and arterial stiffness and to directly compare resistance training-based interventions performed alone with combined aerobic–resistance training. No consistent benefit was observed for wave reflection indices, and evidence for peripheral haemodynamic outcomes was derived from only one acute study and remains insufficient to support conclusions regarding stable or long-term training adaptations.

## 6 Limitations

This study has several limitations.

1. The methodological quality of the included trials was not consistently high, with the main concerns involving the randomization process, outcome measurement, and selective reporting, which may have affected the precision of the effect estimates.
2. Despite focusing on resistance training-based interventions, substantial variation remained in training modality and prescription parameters across studies, and some outcomes were pooled from different measurement indices, making heterogeneity unavoidable.
3. Although the review focused on adults with hypertension and/or elevated vascular risk related to overweight/obesity, the included trials did not all enroll the same population phenotype. Some studies included participants with established hypertension or antihypertensive medication use, whereas others enrolled individuals with elevated blood pressure, prehypertension, overweight/obesity, obesity-related cardiometabolic risk, or overweight/obesity-related vascular risk. Therefore, the pooled estimates may involve population indirectness, and their applicability to adults with both established hypertension and overweight/obesity should be interpreted cautiously.
4. The evidence base was uneven across outcomes: arterial stiffness and FMD were supported by relatively more studies, whereas evidence for wave reflection indices was limited and Peripheral haemodynamic evidence was derived from only one acute post-exercise study; therefore, it cannot be used to infer chronic haemodynamic adaptations to resistance training-based interventions.
5. Few studies examined resistance training combined with aerobic training, and between-subgroup differences were not statistically significant; therefore, its comparative advantage over resistance training alone remains uncertain.
6. Publication bias was suggested for FMD, and several apparent subgroup differences were not supported by meta-regression. Together with methodological limitations, population indirectness, intervention heterogeneity, and sparse evidence for several outcomes, these issues led to conservative GRADE ratings. Therefore, analyses of participant and prescription characteristics should be interpreted as exploratory and hypothesis-generating rather than as evidence for definitive moderator effects or optimal resistance-training prescriptions.

## 7 Future research prospects

1. Future studies should employ larger, more rigorous, and better-reported randomized controlled trials.
2. Core vascular outcomes should be more standardized, particularly the measurement and reporting of arterial stiffness, FMD, and wave reflection indices, with peripheral hemodynamics incorporated into long-term studies.
3. More direct, adequately powered comparisons between resistance training alone and resistance training combined with aerobic training are needed. Future trials should also evaluate dose–response relationships for key prescription variables using designs that can isolate the independent effects of intensity, volume, frequency, and intervention duration.
4. Future research should integrate blood pressure, body composition, and mechanistic markers to clarify how resistance training-based interventions improve vascular function and to inform more precise exercise prescriptions.

## Data Availability

The original contributions presented in the study are included in the article/Supplementary Material, further inquiries can be directed to the corresponding author.
