# Supplementary figures and images for: Can resistance training alone or resistance training combined with aerobic training improve arterial stiffness, endothelial function, and other vascular function indicators in adults with hypertension or overweight/obesity-related vascular risk? A systematic review and meta-analysis of randomized controlled trials
Source: Front Cardiovasc Med. 2026 Jun 24;13:1835366. doi: 10.3389/fcvm.2026.1835366 (PMC13341816; doi:10.3389/fcvm.2026.1835366)

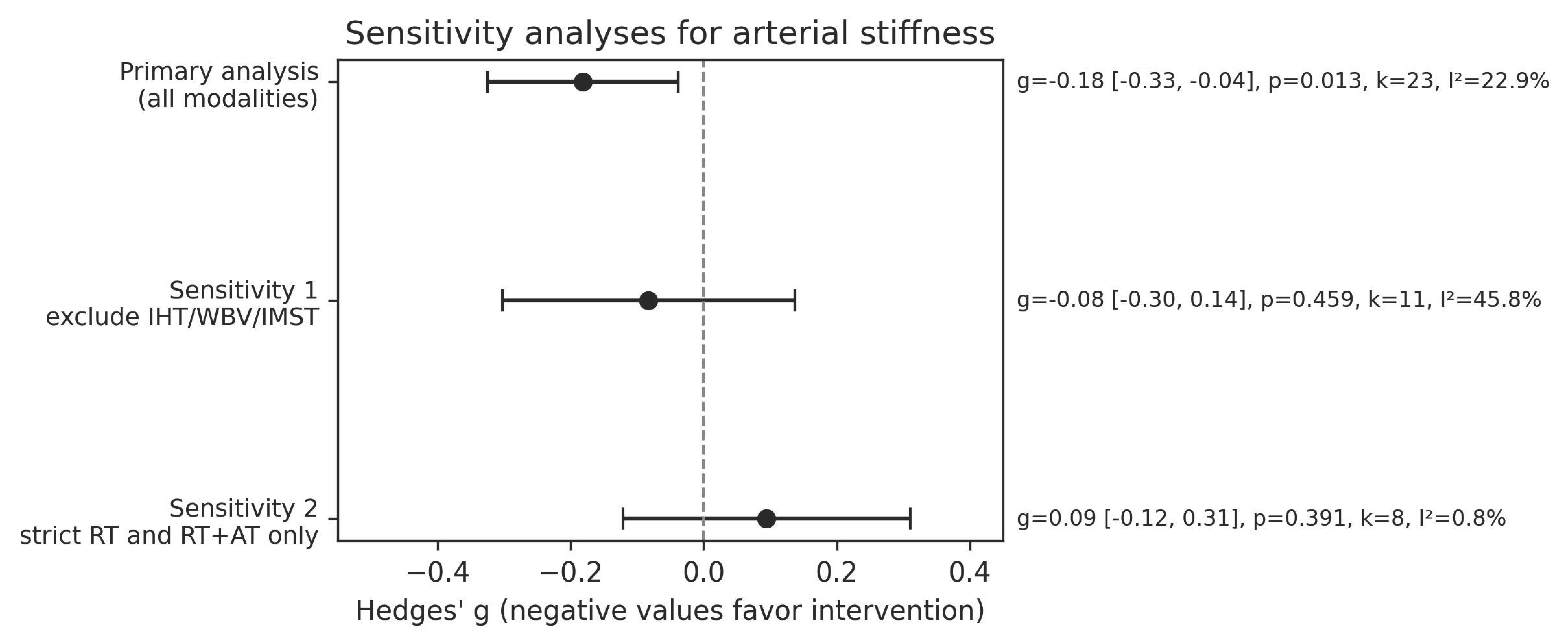

Supplement: Supplementary file 1 [file Supplementaryfile1.zip › Supplementary Fig/Supplementary Fig. 1.tif]

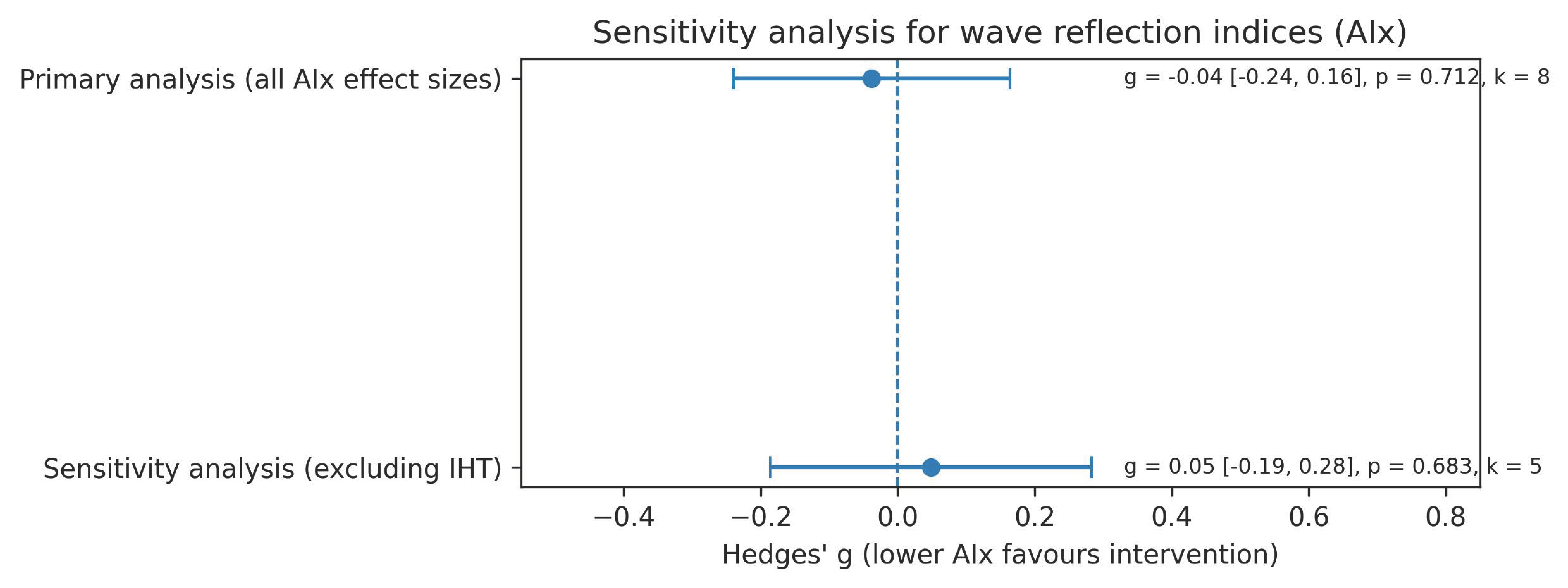

Supplement: Supplementary file 1 [file Supplementaryfile1.zip › Supplementary Fig/Supplementary Fig. 2.tif]

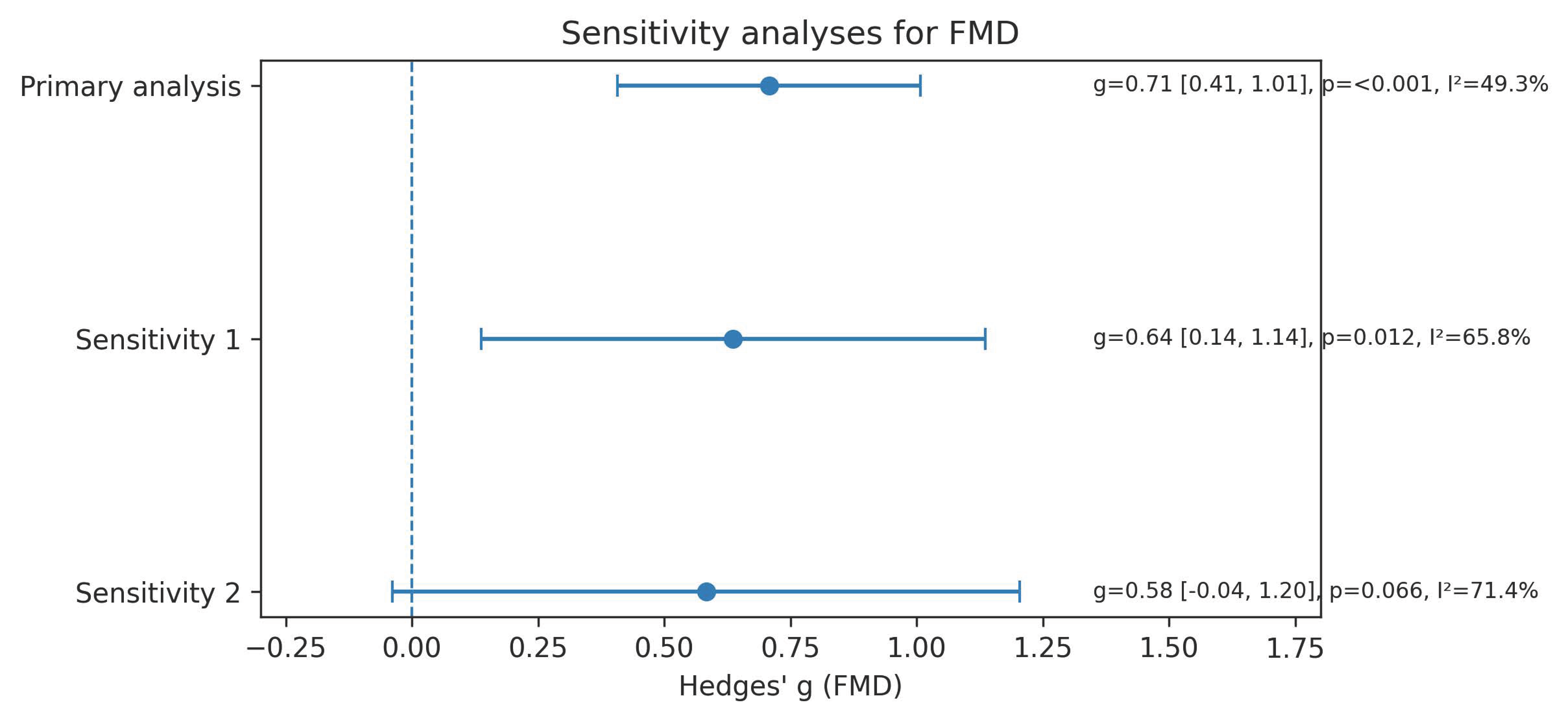

Supplement: Supplementary file 1 [file Supplementaryfile1.zip › Supplementary Fig/Supplementary Fig. 3.tif]

| Study | Experiment | | | Control | | |
| --- | --- | --- | --- | --- | --- | --- |
| Total | MEAN | SD | Total | MEAN | SD |
| Boeno et al., 2020 | 15 | 8.58 | 2.37 | 12 | 6.8 | 2.37 |
| Craighead et al., 2021 | 18 | 7.68 | 2.97 | 18 | 4.50 | 2.97 |


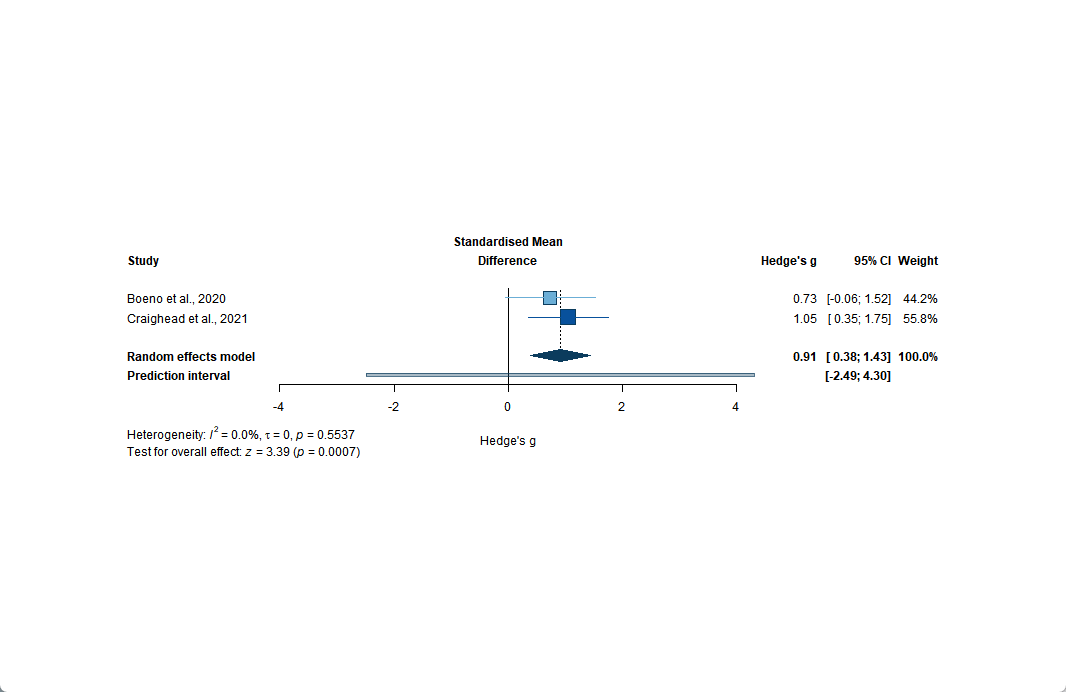

Supplement: Supplementary file 3 [file Supplementaryfile3.zip › Data/FMD/Subgroup analysis/Repetitions/> 15.docx]
