# Supplementary material for: Can resistance training alone or resistance training combined with aerobic training improve arterial stiffness, endothelial function, and other vascular function indicators in adults with hypertension or overweight/obesity-related vascular risk? A systematic review and meta-analysis of randomized controlled trials
Source: Front Cardiovasc Med. 2026 Jun 24;13:1835366. doi: 10.3389/fcvm.2026.1835366 (PMC13341816; doi:10.3389/fcvm.2026.1835366)
Supplement: Supplementary file 2 [file Supplementaryfile2.zip › Supplementary Table S1/Supplementary Table S1.docx]

**Supplementary Table S1. Study-specific verification of eligibility for adults with hypertension and/or individuals at elevated vascular risk related to overweight/obesity**

Operational definition used for this supplementary table: studies were considered eligible if they enrolled adults with diagnosed hypertension, elevated blood pressure, prehypertension, antihypertensive medication use, overweight/obesity, obesity-related cardiometabolic risk, or overweight/obesity-related vascular risk, and reported at least one eligible vascular function outcome.

| **Study** | **Hypertension / elevated BP evidence** | **Overweight / obesity or obesity-related vascular-risk evidence** | **Eligibility under the broader review population** | **Applicability to narrower phenotype** |
| --- | --- | --- | --- | --- |
| **Menêses et al., 2015** | Hypertensive women were recruited; participants used antihypertensive medication and underwent acute combined endurance and resistance exercise. | The article primarily contributes through the hypertension component. Overweight/obesity was not the primary eligibility criterion in the available trial description. | Eligible via hypertension. | Directly applicable to hypertension-related vascular risk; less direct for the narrower overweight/obesity-plus-hypertension phenotype unless baseline adiposity confirms overweight/obesity. |
| **Banks et al., 2024** | Middle-aged/older adults had untreated elevated blood pressure or stage 1 hypertension; eligibility included SBP 120-139 mmHg and/or DBP 80-89 mmHg. | BMI eligibility allowed 18.5-39.9 kg/m2; group mean BMI values were in the overweight range. | Eligible via elevated BP/stage 1 hypertension, with overweight-range group characteristics. | Applicable to the broader elevated-BP vascular-risk population; indirect only if restricted to established hypertension in all participants. |
| **Boeno et al., 2020** | Hypertensive men and women aged 30-59 years receiving at least one antihypertensive drug were enrolled. | BMI was considered in allocation and severe obesity was excluded (BMI >=40 kg/m2), but overweight/obesity was not the main inclusion criterion. | Eligible via diagnosed/treated hypertension. | Directly applicable to medicated hypertension; applicability to the narrower overweight/obesity phenotype depends on baseline adiposity distribution. |
| **Rodrigues et al., 2019** | Hypertensive patients using antihypertensive medication were included in the isometric handgrip training trial. | Baseline BMI values were in the overweight/obese range at group level. | Eligible via hypertension and overweight/obesity-range group characteristics. | Relatively direct for the narrower phenotype, although overweight/obesity was supported mainly by group-level baseline characteristics. |
| **Farah et al., 2018** | Medicated hypertensive adults were recruited; use of antihypertensive medication was an inclusion criterion. | Overweight/obesity was not the primary eligibility criterion in the trial description. | Eligible via treated hypertension. | Directly applicable to hypertension-related vascular risk; indirect for the narrower phenotype if adiposity status is not documented for all participants. |
| **Beck et al., 2013** | Young adults were unmedicated prehypertensive subjects, defined by SBP 120-139 mmHg or DBP 80-89 mmHg across screening visits. | Overweight/obesity was not required; participants were otherwise young and healthy. | Eligible via elevated BP/prehypertension. | Applicable to elevated BP vascular risk, but indirect for established hypertension with overweight/obesity. |
| **Yoon et al., 2019** | Older hypertensive patients were included; baseline BP and antihypertensive medication categories were reported. | Mean BMI was approximately 26 kg/m2 across groups, supporting overweight-range group characteristics. | Eligible via hypertension and overweight-range group characteristics. | Relatively direct for older adults with hypertension and overweight-range BMI. |
| **Miura et al., 2015** | Older women with stage 1 essential hypertension were randomized; hypertensive participants were not taking antihypertensive medication before screening. | Overweight/obesity was not used as the main eligibility criterion in the available description. | Eligible via untreated stage 1 hypertension. | Directly applicable to hypertension; less direct for overweight/obesity unless baseline adiposity confirms this component. |
| **McGowan et al., 2007** | Participants were medicated for hypertension and randomized to bilateral or unilateral isometric handgrip training. | Overweight/obesity was not the main eligibility criterion in the available description. | Eligible via treated hypertension. | Direct for hypertension-related vascular risk; indirect for the narrower adiposity-defined phenotype. |
| **Jung et al., 2024** | Blood pressure was assessed as an outcome; hypertension was discussed as part of the cardiometabolic risk profile of obese older women, but was not clearly required for inclusion. | Participants were older women with sarcopenia and obesity; obesity was defined by body fat percentage >30%. | Eligible via obesity-related vascular/cardiometabolic risk. | Applicable to obesity-related vascular risk; indirect for established hypertension unless participants were confirmed hypertensive. |
| **Franklin et al., 2015** | Participants with a history of hypertension were excluded. | Participants were young obese sedentary women with BMI 30.0-40.0 kg/m2, and endothelial function was assessed. | Eligible via obesity-related vascular risk. | Relevant to obesity-related endothelial dysfunction; not directly applicable to established hypertension. |
| **Dobrosielski et al., 2021** | Uncontrolled hypertension was excluded; hypertension was not required for inclusion. | Adults had BMI >27 kg/m2 and obstructive sleep apnea, a cardiometabolic/vascular-risk condition linked to impaired vascular function. | Eligible via overweight/obesity-related vascular risk. | Applicable to overweight adults with OSA-related vascular risk; indirect for strict hypertension. |
| **Fernandez-del-Valle et al., 2018** | Hypertension was not clearly required as an inclusion criterion. | Participants were young women with obesity; mean BMI was 34.13 +/- 3.16 kg/m2. | Eligible via obesity-related vascular risk. | Relevant to obesity and arterial stiffness; indirect for established hypertension. |
| **Figueroa et al., 2014** | Postmenopausal women had prehypertension or hypertension; mean SBP was 139 mmHg. | Participants were overweight or obese; mean BMI was 34.7 kg/m2 and BMI >25 kg/m2 was required. | Eligible via both elevated BP/hypertension and overweight/obesity. | Highly applicable to the narrower phenotype, although some participants had prehypertension rather than established hypertension. |
| **Ho et al., 2012** | Hypertension was present in a subset and defined by elevated BP or antihypertensive medication; the entire cohort was not necessarily hypertensive. | Participants were overweight or obese adults with BMI 25-40 kg/m2. | Eligible via overweight/obesity-related vascular risk, with partial hypertension representation. | Applicable to overweight/obesity-related vascular risk; partly indirect for strictly hypertensive samples. |
| **Jamka et al., 2021** | Poorly controlled hypertension was excluded; hypertension was not required for inclusion. | Inclusion required obesity: BMI >=30 kg/m2, waist circumference >80 cm, and body fat percentage >=32%. | Eligible via obesity-related vascular risk. | Relevant to abdominal obesity and vascular outcomes; indirect for established hypertension. |
| **Olson et al., 2006** | Participants were required to have BP below stage 1 hypertension and not to be taking antihypertensive medication. | Participants were overweight women with BMI >25 kg/m2. | Eligible via overweight-related vascular risk. | Relevant to overweight-related endothelial function; not directly applicable to established hypertension. |
| **Croymans et al., 2014** | Central and brachial blood pressure were assessed, but hypertension was not required; cardiovascular medications were excluded. | Participants were sedentary overweight/obese young men. | Eligible via overweight/obesity-related vascular risk. | Relevant to obesity-related central BP/vascular outcomes; indirect for established hypertension. |
| **Climie et al., 2019** | Participants were adults at increased cardiovascular disease risk; hypertension was not required as an inclusion criterion. | Participants were sedentary overweight/obese adults with BMI 25-40 kg/m2. | Eligible via overweight/obesity-related vascular risk. | Applicable to overweight/obesity-related vascular risk and sedentary behaviour; indirect for established hypertension. |
| **Craighead et al., 2021** | Participants had above-normal SBP, defined as average SBP >=120 mmHg; randomization considered elevated/stage 1 and stage 2 hypertension categories. | Mean BMI was in the overweight range; severe obesity was excluded. | Eligible via above-normal/elevated BP and overweight-range group characteristics. | Applicable to elevated-BP vascular risk; indirect only if restricted to established hypertension in all participants. |

Note. This table uses the broader population definition adopted to address reviewer concerns: adults with hypertension and/or individuals at elevated vascular risk related to overweight/obesity. "Eligibility under the broader review population" indicates the route through which each study contributed to the review population. The final column clarifies applicability to the narrower phenotype of adults with overweight/obesity and established hypertension. Accordingly, a study may be eligible under the broader definition while still providing indirect evidence for the narrower phenotype.
