# Supplementary material for: Can resistance training alone or resistance training combined with aerobic training improve arterial stiffness, endothelial function, and other vascular function indicators in adults with hypertension or overweight/obesity-related vascular risk? A systematic review and meta-analysis of randomized controlled trials
Source: Front Cardiovasc Med. 2026 Jun 24;13:1835366. doi: 10.3389/fcvm.2026.1835366 (PMC13341816; doi:10.3389/fcvm.2026.1835366)
Supplement: Supplementary file 3 [file Supplementaryfile3.zip › Data/AIX/Data.docx]

| Study | Experiment | | | Control | | |
| --- | --- | --- | --- | --- | --- | --- |
|  | Total | MEAN | SD | Total | MEAN | SD |
| Farah et al., 2018(Home-based IHT-AIx) | 14 | 25.4 | 14.97 | 16 | 29.1 | 9.60 |
| Farah et al., 2018(Supervised IHT-AIx) | 18 | 27.2 | 11.46 | 16 | 29.1 | 9.60 |
| Beck et al., 2013(RT-AIx) | 15 | 3.20 | 10.88 | 15 | -1.21 | 11.27 |
| Yoon et al., 2019(IHT-AIx) | 17 | 33.2 | 8.6 | 18 | 36.1 | 5.5 |
| Dobrosielski et al., 2021(RT+AT-AIx) | 51 | 35.7 | 11.8 | 51 | 36.8 | 11.3 |
| Ho et al., 2012(RT-AIx) | 16 | 29.56 | 10.16 | 16 | 31.31 | 8.00 |
| Ho et al., 2012(RT+AT-AIx) | 17 | 29.59 | 8.33 | 16 | 31.31 | 8.00 |
| Jamka et al., 2021(AT+RT-AIx) | 41 | 34 | 21 | 44 | 29 | 12 |

## ================================

## 0. 环境准备

## ================================

library(meta)

## ================================

## 1. 构建数据

## ================================

data <- data.frame(

Study = c(

"Farah et al., 2018(Home-based IHT-AIx)",

"Farah et al., 2018(Supervised IHT-AIx)",

"Beck et al., 2013(RT-AIx)",

"Yoon et al., 2019(IHT-AIx)",

"Dobrosielski et al., 2021(RT+AT-AIx)",

"Ho et al., 2012(RT-AIx)",

"Ho et al., 2012(RT+AT-AIx)",

"Jamka et al., 2021(AT+RT-AIx)"

),

n_e = c(14, 18, 15, 17, 51, 16, 17, 41),

mean_e = c(25.4, 27.2, 3.20, 33.2, 35.7, 29.56, 29.59, 34),

sd_e = c(14.97, 11.46, 10.88, 8.6, 11.8, 10.16, 8.33, 21),

n_c = c(16, 16, 15, 18, 51, 16, 16, 44),

mean_c = c(29.1, 29.1, -1.21, 36.1, 36.8, 31.31, 31.31, 29),

sd_c = c(9.60, 9.60, 11.27, 5.5, 11.3, 8.00, 8.00, 12)

)

## ================================

## 2. Meta 分析（随机效应）

## ================================

meta_res <- metacont(

n.e = n_e, mean.e = mean_e, sd.e = sd_e,

n.c = n_c, mean.c = mean_c, sd.c = sd_c,

studlab = Study,

data = data,

sm = "SMD",

method.smd = "Hedges",

method.tau = "REML",

method.tau.ci = "J",

comb.random = TRUE,

comb.fixed = FALSE,

prediction = TRUE

)

## ================================

## 3. 配色：渐变蓝

## ================================

pal_fn <- grDevices::colorRampPalette(c("#6BAED6", "#3182BD", "#08519C"))

pal <- pal_fn(200)

col_line <- "#0B3C5D"

map_to_col <- function(x, pal, rng = NULL) {

if (is.null(rng)) rng <- range(x, na.rm = TRUE)

if (!is.finite(diff(rng)) || diff(rng) == 0) return(rep(pal[length(pal)], length(x)))

idx <- floor((x - rng[1]) / diff(rng) * (length(pal) - 1)) + 1

pal[pmax(1, pmin(length(pal), idx))]

}

te_rng <- range(meta_res$TE, na.rm = TRUE)

col_sq_vec <- map_to_col(meta_res$TE, pal, rng = te_rng)

col_predict <- grDevices::adjustcolor(col_line, alpha.f = 0.35)

col_predict_lines <- grDevices::adjustcolor(col_line, alpha.f = 0.70)

## ================================

## 4. 绘制森林图：显示 Test for overall effect + 防挤压

## ================================

forest(

meta_res,

plotwidth = "13cm",

leftcols = c("studlab"),

rightcols = c("effect", "ci", "w.random"),

rightlabs = c("Hedge's g", "95% CI", "Weight"),

col.square = col_sq_vec,

col.square.lines = col_line,

col.study = col_sq_vec,

col.diamond = col_line,

col.diamond.lines = col_line,

col.predict = col_predict,

col.predict.lines = col_predict_lines,

fontsize = 9,

spacing = 1,

fs.hetstat = 9,

fs.axis = 9,

prediction = TRUE,

digits = 2,

print.tau2 = TRUE,

print.tau2.ci = TRUE,

print.tau = TRUE,

## ✅ 关键1：直接让 forest 打印 overall effect 的 Z 与 p（随机效应）

test.overall.random = TRUE,

## ✅ 关键2：在“总体结果”和“异质性/检验信息(x轴下方)”之间加空行，避免挤在一起

addrows.below.overall = 2,

## x轴标题直接用 forest 的 xlab（比 mtext 稳）

xlab = "Hedge's g"

)
