# Supplementary material for: Can resistance training alone or resistance training combined with aerobic training improve arterial stiffness, endothelial function, and other vascular function indicators in adults with hypertension or overweight/obesity-related vascular risk? A systematic review and meta-analysis of randomized controlled trials
Source: Front Cardiovasc Med. 2026 Jun 24;13:1835366. doi: 10.3389/fcvm.2026.1835366 (PMC13341816; doi:10.3389/fcvm.2026.1835366)
Supplement: Supplementary file 3 [file Supplementaryfile3.zip › Data/AIX/Sensitivity Analysis/Sensitivity Analysis.docx]

| Study | Experiment | | | Control | | |
| --- | --- | --- | --- | --- | --- | --- |
|  | Total | MEAN | SD | Total | MEAN | SD |
| Farah et al., 2018(Home-based IHT-AIx) | 14 | 25.4 | 14.97 | 16 | 29.1 | 9.60 |
| Farah et al., 2018(Supervised IHT-AIx) | 18 | 27.2 | 11.46 | 16 | 29.1 | 9.60 |
| Beck et al., 2013(RT-AIx) | 15 | 3.20 | 10.88 | 15 | -1.21 | 11.27 |
| Yoon et al., 2019(IHT-AIx) | 17 | 33.2 | 8.6 | 18 | 36.1 | 5.5 |
| Dobrosielski et al., 2021(RT+AT-AIx) | 51 | 35.7 | 11.8 | 51 | 36.8 | 11.3 |
| Ho et al., 2012(RT-AIx) | 16 | 29.56 | 10.16 | 16 | 31.31 | 8.00 |
| Ho et al., 2012(RT+AT-AIx) | 17 | 29.59 | 8.33 | 16 | 31.31 | 8.00 |
| Jamka et al., 2021(AT+RT-AIx) | 41 | 34 | 21 | 44 | 29 | 12 |

## ================================

## Sensitivity analysis for wave reflection indices (AIx)

## Excluding non-traditional resistance-based modalities

## ================================

## Install packages if needed:

## install.packages(c("meta", "dplyr", "readr"))

library(meta)

library(dplyr)

library(readr)

## ----------------

## 1. Enter data

## ----------------

dat <- data.frame(

Study = c(

"Farah et al., 2018 (Home-based IHT-AIx)",

"Farah et al., 2018 (Supervised IHT-AIx)",

"Beck et al., 2013 (RT-AIx)",

"Yoon et al., 2019 (IHT-AIx)",

"Dobrosielski et al., 2021 (RT+AT-AIx)",

"Ho et al., 2012 (RT-AIx)",

"Ho et al., 2012 (RT+AT-AIx)",

"Jamka et al., 2021 (AT+RT-AIx)"

),

n_e = c(14, 18, 15, 17, 51, 16, 17, 41),

mean_e = c(25.4, 27.2, 3.20, 33.2, 35.7, 29.56, 29.59, 34),

sd_e = c(14.97, 11.46, 10.88, 8.6, 11.8, 10.16, 8.33, 21),

n_c = c(16, 16, 15, 18, 51, 16, 16, 44),

mean_c = c(29.1, 29.1, -1.21, 36.1, 36.8, 31.31, 31.31, 29),

sd_c = c(9.60, 9.60, 11.27, 5.5, 11.3, 8.00, 8.00, 12)

)

dat <- dat %>%

mutate(

modality = case_when(

grepl("IHT", Study) ~ "Non-traditional IHT",

grepl("RT\\+AT|AT\\+RT", Study) ~ "RT+AT",

grepl("\\(RT-", Study) ~ "RT",

TRUE ~ "Other"

),

nontraditional = grepl("IHT", Study)

)

## ----------------

## 2. Primary analysis

## ----------------

m_primary <- metacont(

n.e = n_e, mean.e = mean_e, sd.e = sd_e,

n.c = n_c, mean.c = mean_c, sd.c = sd_c,

studlab = Study,

data = dat,

sm = "SMD",

method.smd = "Hedges",

method.tau = "DL",

common = FALSE,

random = TRUE,

hakn = FALSE

)

## ----------------

## 3. Sensitivity analysis

## Exclude non-traditional modalities.

## In this AIx dataset, non-traditional modalities are IHT studies.

## ----------------

dat_sens <- dat %>%

filter(!nontraditional)

m_sens <- metacont(

n.e = n_e, mean.e = mean_e, sd.e = sd_e,

n.c = n_c, mean.c = mean_c, sd.c = sd_c,

studlab = Study,

data = dat_sens,

sm = "SMD",

method.smd = "Hedges",

method.tau = "DL",

common = FALSE,

random = TRUE,

hakn = FALSE

)

## ----------------

## 4. Export summary results

## ----------------

summary_table <- data.frame(

Analysis = c("Primary analysis (all AIx effect sizes)",

"Sensitivity analysis (excluding IHT)"),

k = c(m_primary$k, m_sens$k),

Hedges_g = c(m_primary$TE.random, m_sens$TE.random),

lower_95_CI = c(m_primary$lower.random, m_sens$lower.random),

upper_95_CI = c(m_primary$upper.random, m_sens$upper.random),

p_value = c(m_primary$pval.random, m_sens$pval.random),

I2_percent = c(m_primary$I2, m_sens$I2),

tau2 = c(m_primary$tau^2, m_sens$tau^2)

)

print(summary_table)

write_csv(summary_table, "sensitivity_analysis_AIx_summary.csv")

## ----------------

## 5. Forest plot for sensitivity analysis

## ----------------

png("sensitivity_forest_excluding_nontraditional_AIx.png",

width = 3000, height = 2100, res = 300)

forest(

m_sens,

sortvar = TE,

prediction = TRUE,

print.tau2 = TRUE,

print.I2 = TRUE,

print.pval.Q = TRUE,

leftcols = c("studlab", "n.e", "n.c"),

leftlabs = c("Study", "Exercise", "Control"),

rightcols = c("effect", "ci", "w.random"),

rightlabs = c("Hedges' g", "95% CI", "Weight"),

smlab = "AIx",

xlab = "Hedges' g (lower AIx favours intervention)",

common = FALSE,

random = TRUE

)

dev.off()

## ----------------

## 6. Optional: save primary forest plot

## ----------------

png("primary_forest_AIx.png",

width = 3000, height = 2400, res = 300)

forest(

m_primary,

sortvar = TE,

prediction = TRUE,

print.tau2 = TRUE,

print.I2 = TRUE,

print.pval.Q = TRUE,

leftcols = c("studlab", "n.e", "n.c"),

leftlabs = c("Study", "Exercise", "Control"),

rightcols = c("effect", "ci", "w.random"),

rightlabs = c("Hedges' g", "95% CI", "Weight"),

smlab = "AIx",

xlab = "Hedges' g (lower AIx favours intervention)",

common = FALSE,

random = TRUE

)

dev.off()
