# Supplementary material for: Can resistance training alone or resistance training combined with aerobic training improve arterial stiffness, endothelial function, and other vascular function indicators in adults with hypertension or overweight/obesity-related vascular risk? A systematic review and meta-analysis of randomized controlled trials
Source: Front Cardiovasc Med. 2026 Jun 24;13:1835366. doi: 10.3389/fcvm.2026.1835366 (PMC13341816; doi:10.3389/fcvm.2026.1835366)
Supplement: Supplementary file 3 [file Supplementaryfile3.zip › Data/Arterial stiffness/Data.docx]

| Study | Experiment | | | Control | | |
| --- | --- | --- | --- | --- | --- | --- |
|  | Total | MEAN | SD | Total | MEAN | SD |
| Banks et al., 2024(RT-cfPWV) | 13 | 6.8 | 1.10 | 13 | 7.2 | 0.92 |
| Rodrigues et al., 2019(IHT-cPWV) | 17 | 8.0 | 1.2 | 16 | 8.8 | 2.0 |
| Rodrigues et al., 2019(IHT-pPWV) | 17 | 8.5 | 1.2 | 16 | 9.4 | 1.6 |
| Farah et al., 2018(home-based IHT-cPWV) | 14 | 7.7 | 1.12 | 16 | 8.8 | 2.00 |
| Farah et al., 2018(supervised IHT-cPWV) | 18 | 8.8 | 1.27 | 16 | 8.8 | 2.00 |
| Farah et al., 2018(home-based IHT-pPWV) | 14 | 8.5 | 1.12 | 16 | 9.4 | 1.60 |
| Farah et al., 2018(supervised IHT-pPWV) | 18 | 8.9 | 2.12 | 16 | 9.4 | 1.60 |
| Beck et al., 2013(RT-crPWV) | 15 | 7.81 | 1.16 | 15 | 7.92 | 1.20 |
| Beck et al., 2013(RT-fdPWV) | 15 | 9.39 | 1.39 | 15 | 8.60 | 0.97 |
| Beck et al., 2013(RT-cfPWV) | 15 | 6.81 | 0.70 | 15 | 6.55 | 0.70 |
| Yoon et al., 2019(IHT-cfPWV) | 17 | 9.9 | 2.1 | 18 | 10.3 | 1.4 |
| Miura et al., 2015(CRT-baPWV) | 92 | 1821.0 | 311.8 | 92 | 1841.9 | 294.9 |
| Miura et al., 2015(CRT-baPWV) | 108 | 1552 | 208.6 | 108 | 1641.5 | 203.6 |
| Jung et al., 2024(CRT-baPWV) | 14 | 1718.82 | 215.67 | 14 | 1856.11 | 159.77 |
| Dobrosielski et al., 2021(RT+AT-cfPWV) | 51 | 8.3 | 1.4 | 51 | 8.1 | 1.6 |
| Fernandez-del-Valle et al., 2018(RT-PWV) | 6 | 6.73 | 0.94 | 5 | 6.70 | 0.82 |
| Figueroa et al., 2014(RT+WBV-aPWV) | 13 | 12.2 | 2.16 | 12 | 12.4 | 1.39 |
| Figueroa et al., 2014(RT+WBV-faPWV) | 13 | 9.4 | 1.08 | 12 | 9.7 | 1.04 |
| Figueroa et al., 2014(RT+WBV-baPWV) | 13 | 12.8 | 1.44 | 12 | 14.0 | 1.39 |
| Jamka et al., 2021(RT+AT-cfPWV) | 41 | 6.7 | 1.4 | 44 | 6.5 | 0.8 |
| Croymans et al., 2014(RT-cfPWV) | 28 | 6.67 | 1.19 | 8 | 7.27 | 0.30 |
| Craighead et al., 2021(IMST-cfPWV) | 18 | 9.64 | 1.53 | 18 | 9.92 | 2.33 |
| Craighead et al., 2021(IMST-cfPWV) | 18 | 10.01 | 5.12 | 18 | 9.08 | 3.16 |

## ================================

## 0. 环境准备

## ================================

library(meta)

## ================================

## 1. 构建数据

## ================================

data <- data.frame(

Study = c(

"Banks et al., 2024(RT-cfPWV)",

"Rodrigues et al., 2019(IHT-cPWV)",

"Rodrigues et al., 2019(IHT-pPWV)",

"Farah et al., 2018(home-based IHT-cPWV)",

"Farah et al., 2018(supervised IHT-cPWV)",

"Farah et al., 2018(home-based IHT-pPWV)",

"Farah et al., 2018(supervised IHT-pPWV)",

"Beck et al., 2013(RT-crPWV)",

"Beck et al., 2013(RT-fdPWV)",

"Beck et al., 2013(RT-cfPWV)",

"Yoon et al., 2019(IHT-cfPWV)",

"Miura et al., 2015(CRT-baPWV)",

"Miura et al., 2015(CRT-baPWV)",

"Jung et al., 2024(CRT-baPWV)",

"Dobrosielski et al., 2021(RT+AT-cfPWV)",

"Fernandez-del-Valle et al., 2018(RT-PWV)",

"Figueroa et al., 2014(RT+WBV-aPWV)",

"Figueroa et al., 2014(RT+WBV-faPWV)",

"Figueroa et al., 2014(RT+WBV-baPWV)",

"Jamka et al., 2021(RT+AT-cfPWV)",

"Croymans et al., 2014(RT-cfPWV)",

"Craighead et al., 2021(IMST-cfPWV)",

"Craighead et al., 2021(IMST-cfPWV)"

),

n_e = c(

13, 17, 17, 14, 18, 14, 18, 15, 15, 15, 17, 92, 108, 14, 51, 6, 13, 13, 13, 41, 28, 18, 18

),

mean_e = c(

6.8, 8.0, 8.5, 7.7, 8.8, 8.5, 8.9, 7.81, 9.39, 6.81, 9.9, 1821.0, 1552.0, 1718.82, 8.3, 6.73, 12.2, 9.4, 12.8, 6.7, 6.67, 9.64, 10.01

),

sd_e = c(

1.10, 1.2, 1.2, 1.12, 1.27, 1.12, 2.12, 1.16, 1.39, 0.70, 2.1, 311.8, 208.6, 215.67, 1.4, 0.94, 2.16, 1.08, 1.44, 1.4, 1.19, 1.53, 5.12

),

n_c = c(

13, 16, 16, 16, 16, 16, 16, 15, 15, 15, 18, 92, 108, 14, 51, 5, 12, 12, 12, 44, 8, 18, 18

),

mean_c = c(

7.2, 8.8, 9.4, 8.8, 8.8, 9.4, 9.4, 7.92, 8.60, 6.55, 10.3, 1841.9, 1641.5, 1856.11, 8.1, 6.70, 12.4, 9.7, 14.0, 6.5, 7.27, 9.92, 9.08

),

sd_c = c(

0.92, 2.0, 1.6, 2.00, 2.00, 1.60, 1.60, 1.20, 0.97, 0.70, 1.4, 294.9, 203.6, 159.77, 1.6, 0.82, 1.39, 1.04, 1.39, 0.8, 0.30, 2.33, 3.16

)

)

## ================================

## 2. Meta 分析（随机效应）

## ================================

meta_res <- metacont(

n.e = n_e, mean.e = mean_e, sd.e = sd_e,

n.c = n_c, mean.c = mean_c, sd.c = sd_c,

studlab = Study,

data = data,

sm = "SMD",

method.smd = "Hedges",

method.tau = "REML",

method.tau.ci = "J",

comb.random = TRUE,

comb.fixed = FALSE,

prediction = TRUE

)

## ================================

## 3. 配色：渐变蓝

## ================================

pal_fn <- grDevices::colorRampPalette(c("#6BAED6", "#3182BD", "#08519C"))

pal <- pal_fn(200)

col_line <- "#0B3C5D"

map_to_col <- function(x, pal, rng = NULL) {

if (is.null(rng)) rng <- range(x, na.rm = TRUE)

if (!is.finite(diff(rng)) || diff(rng) == 0) return(rep(pal[length(pal)], length(x)))

idx <- floor((x - rng[1]) / diff(rng) * (length(pal) - 1)) + 1

pal[pmax(1, pmin(length(pal), idx))]

}

te_rng <- range(meta_res$TE, na.rm = TRUE)

col_sq_vec <- map_to_col(meta_res$TE, pal, rng = te_rng)

col_predict <- grDevices::adjustcolor(col_line, alpha.f = 0.35)

col_predict_lines <- grDevices::adjustcolor(col_line, alpha.f = 0.70)

## ================================

## 4. 绘制森林图：显示 Test for overall effect + 防挤压

## ================================

forest(

meta_res,

plotwidth = "13cm",

leftcols = c("studlab"),

rightcols = c("effect", "ci", "w.random"),

rightlabs = c("Hedge's g", "95% CI", "Weight"),

col.square = col_sq_vec,

col.square.lines = col_line,

col.study = col_sq_vec,

col.diamond = col_line,

col.diamond.lines = col_line,

col.predict = col_predict,

col.predict.lines = col_predict_lines,

fontsize = 9,

spacing = 1,

fs.hetstat = 9,

fs.axis = 9,

prediction = TRUE,

digits = 2,

print.tau2 = TRUE,

print.tau2.ci = TRUE,

print.tau = TRUE,

test.overall.random = TRUE,

addrows.below.overall = 2,

xlab = "Hedge's g"

)
