# Supplementary material for: Can resistance training alone or resistance training combined with aerobic training improve arterial stiffness, endothelial function, and other vascular function indicators in adults with hypertension or overweight/obesity-related vascular risk? A systematic review and meta-analysis of randomized controlled trials
Source: Front Cardiovasc Med. 2026 Jun 24;13:1835366. doi: 10.3389/fcvm.2026.1835366 (PMC13341816; doi:10.3389/fcvm.2026.1835366)

| X：Duration | Y：（effect size）Hedge's g | Weight（%） |
| --- | --- | --- |
| 9 | -0.38 | 3.0 |
| 12 | -0.48 | 3.6 |
| 12 | -0.62 | 3.5 |
| 12 | -0.65 | 3.3 |
| 12 | 0.00 | 3.8 |
| 12 | -0.63 | 3.3 |
| 12 | -0.26 | 3.8 |
| 8 | -0.09 | 3.4 |
| 8 | 0.64 | 3.3 |
| 8 | 0.36 | 3.4 |
| 12 | -0.22 | 3.9 |
| 12 | -0.07 | 11.0 |
| 12 | -0.43 | 11.6 |
| 12 | -0.70 | 3.1 |
| 6 | 0.13 | 8.2 |
| 3 | 0.03 | 1.4 |
| 12 | -0.11 | 2.9 |
| 12 | -0.27 | 2.9 |
| 12 | -0.82 | 2.7 |
| 12 | 0.18 | 7.3 |
| 12 | -0.55 | 2.9 |
| 6 | -0.14 | 4.0 |
| 6 | 0.21 | 4.0 |

# 加载必要的包

library(metafor)

# 创建数据框（按 Duration.docx 替换：X=Duration, Y=Hedge's g, Weight%）

df <- data.frame(

Duration = c(

9, 12, 12, 12, 12, 12, 12,

8, 8, 8,

12, 12, 12, 12,

6, 3,

12, 12, 12, 12, 12,

6, 6

),

g = c(

-0.38, -0.48, -0.62, -0.65, 0.00, -0.63, -0.26,

-0.09, 0.64, 0.36,

-0.22, -0.07, -0.43, -0.70,

0.13, 0.03,

-0.11, -0.27, -0.82, 0.18, -0.55,

-0.14, 0.21

),

Weight = c(

3.0, 3.6, 3.5, 3.3, 3.8, 3.3, 3.8,

3.4, 3.3, 3.4,

3.9, 11.0, 11.6, 3.1,

8.2, 1.4,

2.9, 2.9, 2.7, 7.3, 2.9,

4.0, 4.0

)

)

# 将 Duration 转为数值

df$Duration_num <- as.numeric(df$Duration)

# 计算方差（权重为1/vi）

df$vi <- 1 / df$Weight

# 执行 Meta 回归分析（混合效应模型）

res <- rma(yi = g, vi = vi, mods = ~ Duration_num, data = df)

# 提取统计结果（稳健写法：从 summary(res) 的系数表取数值）

tab <- coef(summary(res)) # estimate, se, zval, pval, ci.lb, ci.ub

beta <- round(tab[2, "estimate"], 3)

ci_lb <- round(tab[2, "ci.lb"], 3)

ci_ub <- round(tab[2, "ci.ub"], 3)

p_value <- ifelse(tab[2, "pval"] < 0.001, "< 0.001", round(tab[2, "pval"], 3))

# 绘制气泡图

regplot(

res,

mod = "Duration_num",

pred = TRUE,

ci = TRUE,

pi = TRUE,

shade = c("gray85", "gray95"),

xlab = "Duration",

ylab = "Hedge's g",

psize = sqrt(df$Weight),

col = "black",

las = 1

)

# 添加统计结果文本

text(

x = max(df$Duration_num) - 0.2 * (max(df$Duration_num) - min(df$Duration_num)),

y = max(df$g) - 0.1 * (max(df$g) - min(df$g)),

labels = paste0(

"β=", beta, "\n",

"95% CI: [", ci_lb, ", ", ci_ub, "]\n",

"P=", p_value

),

pos = 2,

cex = 1.1,

col = "black",

font = 2

)

# 添加紧凑图例（稳定版）

legend(

"bottomright",

legend = c(

"Studies",

"Regression Line",

"95% Confidence Interval",

"95% Prediction Interval"

),

pch = c(19, NA, 15, 15),

lty = c(0, 1, 0, 0),

pt.cex = c(1, NA, 2, 2),

col = c("black", "black", "gray85", "gray95"),

bty = "o",

bg = "white",

cex = 1

)


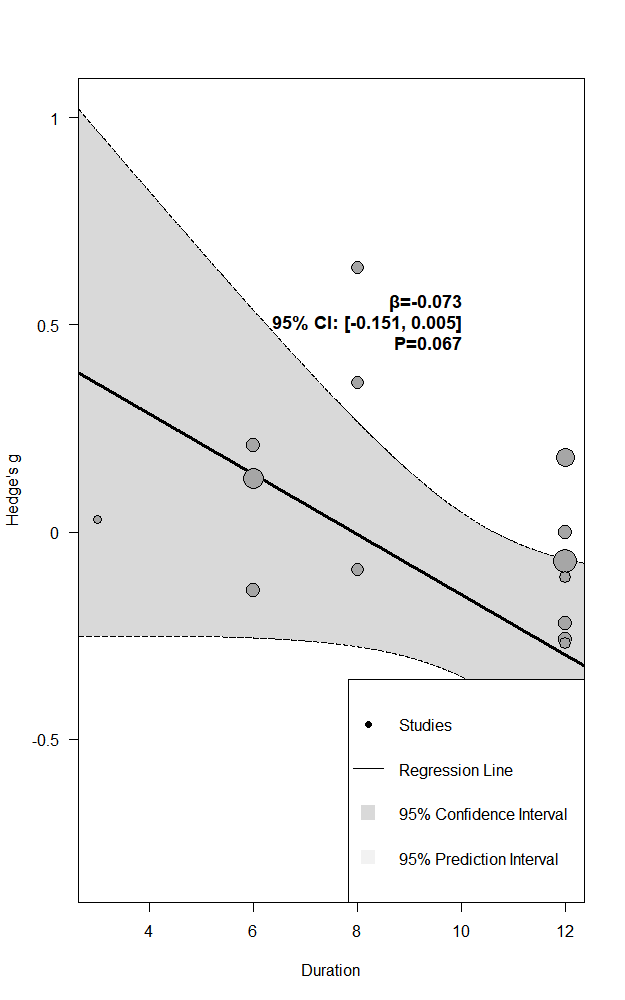

Supplement: Supplementary file 3 [file Supplementaryfile3.zip › Data/Arterial stiffness/Meta-regression analysis/Duration.docx]
