# Supplementary material for: Can resistance training alone or resistance training combined with aerobic training improve arterial stiffness, endothelial function, and other vascular function indicators in adults with hypertension or overweight/obesity-related vascular risk? A systematic review and meta-analysis of randomized controlled trials
Source: Front Cardiovasc Med. 2026 Jun 24;13:1835366. doi: 10.3389/fcvm.2026.1835366 (PMC13341816; doi:10.3389/fcvm.2026.1835366)

| X：Sets | Y：（effect size）Hedge's g | Weight（%） |
| --- | --- | --- |
| 4 | -0.48 | 3.6 |
| 4 | -0.62 | 3.5 |
| 4 | -0.65 | 3.3 |
| 4 | 0.00 | 3.8 |
| 4 | -0.63 | 3.3 |
| 4 | -0.26 | 3.8 |
| 2 | -0.09 | 3.4 |
| 2 | 0.64 | 3.3 |
| 2 | 0.36 | 3.4 |
| 4 | -0.22 | 3.9 |
| 35 | -0.07 | 11.0 |
| 35 | -0.43 | 11.6 |
| 2-4 | -0.70 | 3.1 |
| 2 | 0.13 | 8.2 |
| 3 | 0.03 | 1.4 |
| 1-6 | -0.11 | 2.9 |
| 1-6 | -0.27 | 2.9 |
| 1-6 | -0.82 | 2.7 |
| 2-3 | -0.55 | 2.9 |
| 5 | -0.14 | 4.0 |
| 5 | 0.21 | 4.0 |

# 加载必要的包

library(metafor)

# 创建数据框（已按 Sets.docx 替换：X=Sets, Y=Hedge's g, Weight%）

df <- data.frame(

Sets = c(

"4", "4", "4", "4", "4", "4",

"2", "2", "2",

"4",

"35", "35",

"2-4",

"2",

"3",

"1-6", "1-6", "1-6",

"2-3",

"5", "5"

),

g = c(

-0.48, -0.62, -0.65, 0.00, -0.63, -0.26,

-0.09, 0.64, 0.36,

-0.22,

-0.07, -0.43,

-0.70,

0.13,

0.03,

-0.11, -0.27, -0.82,

-0.55,

-0.14, 0.21

),

Weight = c(

3.6, 3.5, 3.3, 3.8, 3.3, 3.8,

3.4, 3.3, 3.4,

3.9,

11.0, 11.6,

3.1,

8.2,

1.4,

2.9, 2.9, 2.7,

2.9,

4.0, 4.0

)

)

# 将 Sets 转为数值

# 若为区间（如 2-4），取中点用于回归

df$Sets_num <- ifelse(

grepl("-", df$Sets),

sapply(strsplit(df$Sets, "-"), function(x) mean(as.numeric(x))),

as.numeric(df$Sets)

)

# 计算方差（权重为1/vi）

df$vi <- 1 / df$Weight

# 执行Meta回归分析（混合效应模型）

res <- rma(yi = g, vi = vi, mods = ~ Sets_num, data = df)

# 提取统计结果（稳健写法：从 summary(res) 的系数表取数值）

tab <- coef(summary(res)) # estimate, se, zval, pval, ci.lb, ci.ub

beta <- round(tab[2, "estimate"], 3)

ci_lb <- round(tab[2, "ci.lb"], 3)

ci_ub <- round(tab[2, "ci.ub"], 3)

p_value <- ifelse(tab[2, "pval"] < 0.001, "< 0.001", round(tab[2, "pval"], 3))

# 绘制气泡图

regplot(

res,

mod = "Sets_num",

pi = TRUE,

pred = TRUE,

xlab = "Sets",

ylab = "Hedge's g",

psize = sqrt(df$Weight),

col = "black",

ci.col = "darkgray",

pi.col = "lightgray",

las = 1

)

# 添加统计结果文本

text(

x = max(df$Sets_num) - 0.2*(max(df$Sets_num) - min(df$Sets_num)),

y = max(df$g) - 0.1*(max(df$g) - min(df$g)),

labels = paste0(

"β=", beta, "\n",

"95% CI: [", ci_lb, ", ", ci_ub, "]\n",

"P=", p_value

),

pos = 2,

cex = 1.1,

col = "black",

font = 2

)

# 添加紧凑图例

legend(

"bottomright",

legend = c("Studies", "Regression Line", "95% Confidence Interval", "95% Prediction Interval"),

pch = c(19, NA, NA, NA),

lty = c(NA, 1, NA, NA),

fill = c(NA, NA, "darkgray", "lightgray"),

border = c(NA, NA, "darkgray", "lightgray"),

col = c("gray60", "black", NA, NA),

pt.cex = 1.0,

cex = 1,

x.intersp = 1,

y.intersp = 1,

bg = "white"

)


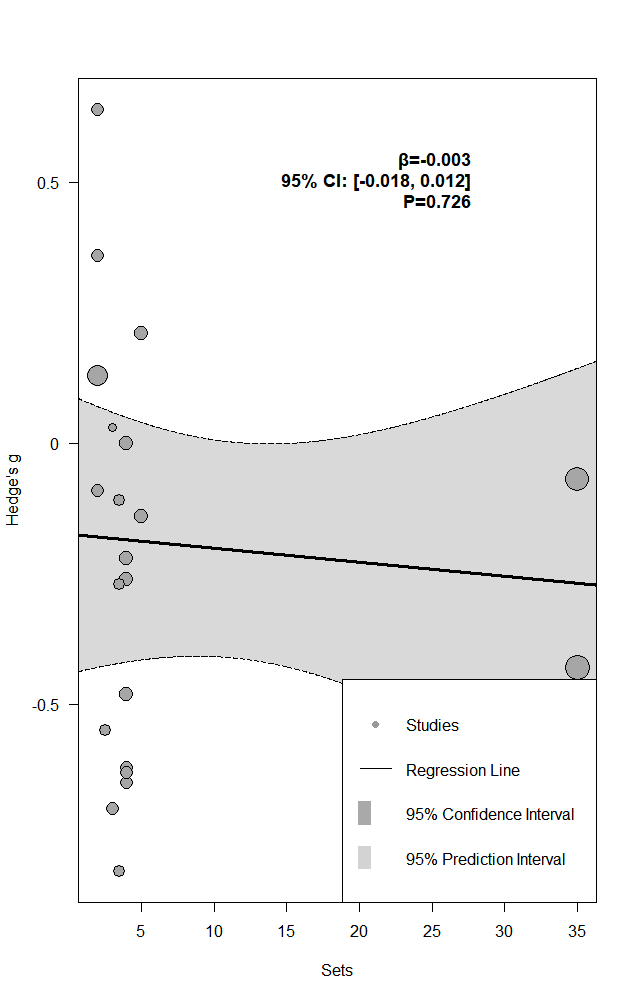

Supplement: Supplementary file 3 [file Supplementaryfile3.zip › Data/Arterial stiffness/Meta-regression analysis/Sets.docx]
