# Supplementary material for: Can resistance training alone or resistance training combined with aerobic training improve arterial stiffness, endothelial function, and other vascular function indicators in adults with hypertension or overweight/obesity-related vascular risk? A systematic review and meta-analysis of randomized controlled trials
Source: Front Cardiovasc Med. 2026 Jun 24;13:1835366. doi: 10.3389/fcvm.2026.1835366 (PMC13341816; doi:10.3389/fcvm.2026.1835366)
Supplement: Supplementary file 3 [file Supplementaryfile3.zip › Data/Arterial stiffness/Sensitivity Analysis/Sensitivity Analysis.docx]

| Study | Experiment | | | Control | | |
| --- | --- | --- | --- | --- | --- | --- |
|  | Total | MEAN | SD | Total | MEAN | SD |
| Banks et al., 2024(RT-cfPWV) | 13 | 6.8 | 1.10 | 13 | 7.2 | 0.92 |
| Rodrigues et al., 2019(IHT-cPWV) | 17 | 8.0 | 1.2 | 16 | 8.8 | 2.0 |
| Rodrigues et al., 2019(IHT-pPWV) | 17 | 8.5 | 1.2 | 16 | 9.4 | 1.6 |
| Farah et al., 2018(home-based IHT-cPWV) | 14 | 7.7 | 1.12 | 16 | 8.8 | 2.00 |
| Farah et al., 2018(supervised IHT-cPWV) | 18 | 8.8 | 1.27 | 16 | 8.8 | 2.00 |
| Farah et al., 2018(home-based IHT-pPWV) | 14 | 8.5 | 1.12 | 16 | 9.4 | 1.60 |
| Farah et al., 2018(supervised IHT-pPWV) | 18 | 8.9 | 2.12 | 16 | 9.4 | 1.60 |
| Beck et al., 2013(RT-crPWV) | 15 | 7.81 | 1.16 | 15 | 7.92 | 1.20 |
| Beck et al., 2013(RT-fdPWV) | 15 | 9.39 | 1.39 | 15 | 8.60 | 0.97 |
| Beck et al., 2013(RT-cfPWV) | 15 | 6.81 | 0.70 | 15 | 6.55 | 0.70 |
| Yoon et al., 2019(IHT-cfPWV) | 17 | 9.9 | 2.1 | 18 | 10.3 | 1.4 |
| Miura et al., 2015(CRT-baPWV) | 92 | 1821.0 | 311.8 | 92 | 1841.9 | 294.9 |
| Miura et al., 2015(CRT-baPWV) | 108 | 1552 | 208.6 | 108 | 1641.5 | 203.6 |
| Jung et al., 2024(CRT-baPWV) | 14 | 1718.82 | 215.67 | 14 | 1856.11 | 159.77 |
| Dobrosielski et al., 2021(RT+AT-cfPWV) | 51 | 8.3 | 1.4 | 51 | 8.1 | 1.6 |
| Fernandez-del-Valle et al., 2018(RT-PWV) | 6 | 6.73 | 0.94 | 5 | 6.70 | 0.82 |
| Figueroa et al., 2014(RT+WBV-aPWV) | 13 | 12.2 | 2.16 | 12 | 12.4 | 1.39 |
| Figueroa et al., 2014(RT+WBV-faPWV) | 13 | 9.4 | 1.08 | 12 | 9.7 | 1.04 |
| Figueroa et al., 2014(RT+WBV-baPWV) | 13 | 12.8 | 1.44 | 12 | 14.0 | 1.39 |
| Jamka et al., 2021(RT+AT-cfPWV) | 41 | 6.7 | 1.4 | 44 | 6.5 | 0.8 |
| Croymans et al., 2014(RT-cfPWV) | 28 | 6.67 | 1.19 | 8 | 7.27 | 0.30 |
| Craighead et al., 2021(IMST-cfPWV) | 18 | 9.64 | 1.53 | 18 | 9.92 | 2.33 |
| Craighead et al., 2021(IMST-cfPWV) | 18 | 10.01 | 5.12 | 18 | 9.08 | 3.16 |

## ============================================================

## Sensitivity analyses excluding non-traditional resistance-based modalities

## Outcome: arterial stiffness

## Effect size: Hedges' g / standardized mean difference

## Negative Hedges' g favors intervention because lower arterial stiffness is beneficial.

## ============================================================

## install.packages(c("meta", "dplyr"))

library(meta)

library(dplyr)

## -----------------------------

## 1. Enter data

## -----------------------------

dat <- data.frame(

Study = c(

"Banks et al., 2024 (RT-cfPWV)",

"Rodrigues et al., 2019 (IHT-cPWV)",

"Rodrigues et al., 2019 (IHT-pPWV)",

"Farah et al., 2018 (home-based IHT-cPWV)",

"Farah et al., 2018 (supervised IHT-cPWV)",

"Farah et al., 2018 (home-based IHT-pPWV)",

"Farah et al., 2018 (supervised IHT-pPWV)",

"Beck et al., 2013 (RT-crPWV)",

"Beck et al., 2013 (RT-fdPWV)",

"Beck et al., 2013 (RT-cfPWV)",

"Yoon et al., 2019 (IHT-cfPWV)",

"Miura et al., 2015 (CRT-baPWV)",

"Miura et al., 2015 (CRT-baPWV)",

"Jung et al., 2024 (CRT-baPWV)",

"Dobrosielski et al., 2021 (RT+AT-cfPWV)",

"Fernandez-del-Valle et al., 2018 (RT-PWV)",

"Figueroa et al., 2014 (RT+WBV-aPWV)",

"Figueroa et al., 2014 (RT+WBV-faPWV)",

"Figueroa et al., 2014 (RT+WBV-baPWV)",

"Jamka et al., 2021 (RT+AT-cfPWV)",

"Croymans et al., 2014 (RT-cfPWV)",

"Craighead et al., 2021 (IMST-cfPWV)",

"Craighead et al., 2021 (IMST-cfPWV)"

),

n.e = c(13,17,17,14,18,14,18,15,15,15,17,92,108,14,51,6,13,13,13,41,28,18,18),

mean.e = c(6.8,8.0,8.5,7.7,8.8,8.5,8.9,7.81,9.39,6.81,9.9,1821.0,1552,1718.82,8.3,6.73,12.2,9.4,12.8,6.7,6.67,9.64,10.01),

sd.e = c(1.10,1.2,1.2,1.12,1.27,1.12,2.12,1.16,1.39,0.70,2.1,311.8,208.6,215.67,1.4,0.94,2.16,1.08,1.44,1.4,1.19,1.53,5.12),

n.c = c(13,16,16,16,16,16,16,15,15,15,18,92,108,14,51,5,12,12,12,44,8,18,18),

mean.c = c(7.2,8.8,9.4,8.8,8.8,9.4,9.4,7.92,8.60,6.55,10.3,1841.9,1641.5,1856.11,8.1,6.70,12.4,9.7,14.0,6.5,7.27,9.92,9.08),

sd.c = c(0.92,2.0,1.6,2.00,2.00,1.60,1.60,1.20,0.97,0.70,1.4,294.9,203.6,159.77,1.6,0.82,1.39,1.04,1.39,0.8,0.30,2.33,3.16),

Modality = c(

"RT","IHT","IHT","IHT","IHT","IHT","IHT","RT","RT","RT","IHT",

"CRT","CRT","CRT","RT+AT","RT","WBV","WBV","WBV","RT+AT","RT","IMST","IMST"

)

)

## -----------------------------

## 2. Classify modalities

## -----------------------------

dat <- dat %>%

mutate(

nontraditional_primary = Modality %in% c("IHT", "WBV", "SRA", "IMST"),

strict_conventional = Modality %in% c("RT", "RT+AT")

)

## Sensitivity 1:

## Exclude clearly non-traditional modalities: IHT, WBV, SRA, IMST.

## Retain conventional dynamic RT, circuit RT, and RT+AT.

dat_sens1 <- dat %>%

filter(!nontraditional_primary)

## Sensitivity 2:

## Stricter analysis retaining only conventional dynamic RT and RT+AT.

## This excludes circuit RT as well.

dat_sens2 <- dat %>%

filter(strict_conventional)

## -----------------------------

## 3. Run meta-analyses

## -----------------------------

run_meta <- function(data_input) {

metacont(

n.e = n.e, mean.e = mean.e, sd.e = sd.e,

n.c = n.c, mean.c = mean.c, sd.c = sd.c,

studlab = Study,

data = data_input,

sm = "SMD",

method.smd = "Hedges",

method.tau = "DL", # Change to "REML" if your manuscript uses REML for the primary meta-analysis.

common = FALSE,

random = TRUE,

hakn = FALSE,

prediction = TRUE

)

}

m_all <- run_meta(dat)

m_sens1 <- run_meta(dat_sens1)

m_sens2 <- run_meta(dat_sens2)

summary(m_all)

summary(m_sens1)

summary(m_sens2)

## -----------------------------

## 4. Forest plot for sensitivity analysis 1

## -----------------------------

png("Sensitivity_forest_excluding_IHT_WBV_IMST_arterial_stiffness.png",

width = 3200, height = 2400, res = 300)

forest(

m_sens1,

sortvar = TE,

prediction = TRUE,

print.tau2 = TRUE,

print.I2 = TRUE,

print.pval.Q = TRUE,

leftcols = c("studlab", "n.e", "n.c"),

leftlabs = c("Study", "Intervention n", "Control n"),

rightcols = c("effect", "ci", "w.random"),

rightlabs = c("Hedges' g", "95% CI", "Weight"),

xlab = "Hedges' g (negative values favor intervention)",

smlab = "Sensitivity analysis: excluding IHT, WBV, SRA, and IMST",

col.square = "black",

col.square.lines = "black",

col.diamond = "black",

col.diamond.lines = "black",

col.predict = "gray40"

)

dev.off()

## -----------------------------

## 5. Robustness summary plot:

## primary analysis vs sensitivity analyses

## -----------------------------

get_row <- function(m, label) {

data.frame(

Analysis = label,

k = m$k,

g = m$TE.random,

lower = m$lower.random,

upper = m$upper.random,

p = m$pval.random,

I2 = as.numeric(m$I2)

)

}

sumdat <- bind_rows(

get_row(m_all, "Primary analysis: all modalities"),

get_row(m_sens1, "Sensitivity 1: exclude IHT/WBV/SRA/IMST"),

get_row(m_sens2, "Sensitivity 2: strict RT and RT+AT only")

)

print(sumdat)

png("Sensitivity_summary_arterial_stiffness.png",

width = 3200, height = 1600, res = 300)

par(mar = c(5, 12, 4, 5))

ypos <- rev(seq_len(nrow(sumdat)))

plot(

sumdat$g, ypos,

xlim = range(c(sumdat$lower, sumdat$upper, 0), na.rm = TRUE) + c(-0.10, 0.10),

ylim = c(0.5, nrow(sumdat) + 0.5),

yaxt = "n",

pch = 19,

xlab = "Hedges' g (negative values favor intervention)",

ylab = "",

main = "Sensitivity analyses for arterial stiffness"

)

abline(v = 0, lty = 2)

axis(2, at = ypos, labels = sumdat$Analysis, las = 1)

segments(sumdat$lower, ypos, sumdat$upper, ypos)

arrows(sumdat$lower, ypos, sumdat$upper, ypos, angle = 90, code = 3, length = 0.04)

text(

x = max(sumdat$upper, na.rm = TRUE) + 0.05,

y = ypos,

labels = sprintf("g = %.2f [%.2f, %.2f]; p = %.3f; k = %d; I² = %.1f%%",

sumdat$g, sumdat$lower, sumdat$upper, sumdat$p, sumdat$k, sumdat$I2),

pos = 4,

cex = 0.75

)

dev.off()

## -----------------------------

## 6. Optional: save numerical results

## -----------------------------

write.csv(sumdat, "Sensitivity_summary_arterial_stiffness.csv", row.names = FALSE)
