# Supplementary material for: Can resistance training alone or resistance training combined with aerobic training improve arterial stiffness, endothelial function, and other vascular function indicators in adults with hypertension or overweight/obesity-related vascular risk? A systematic review and meta-analysis of randomized controlled trials
Source: Front Cardiovasc Med. 2026 Jun 24;13:1835366. doi: 10.3389/fcvm.2026.1835366 (PMC13341816; doi:10.3389/fcvm.2026.1835366)
Supplement: Supplementary file 3 [file Supplementaryfile3.zip › Data/Arterial stiffness/Subgroup analysis/Duration(wk)/9.docx]

| Study | Experiment | | | Control | | |
| --- | --- | --- | --- | --- | --- | --- |
|  | Total | MEAN | SD | Total | MEAN | SD |
| Banks et al., 2024 | 13 | 6.8 | 1.10 | 13 | 7.2 | 0.92 |
