# Supplementary material for: Can resistance training alone or resistance training combined with aerobic training improve arterial stiffness, endothelial function, and other vascular function indicators in adults with hypertension or overweight/obesity-related vascular risk? A systematic review and meta-analysis of randomized controlled trials
Source: Front Cardiovasc Med. 2026 Jun 24;13:1835366. doi: 10.3389/fcvm.2026.1835366 (PMC13341816; doi:10.3389/fcvm.2026.1835366)

| Study | Experiment | | | Control | | |
| --- | --- | --- | --- | --- | --- | --- |
|  | Total | MEAN | SD | Total | MEAN | SD |
| Banks et al., 2024 | 13 | 6.8 | 1.10 | 13 | 7.2 | 0.92 |
| Rodrigues et al., 2019 | 17 | 8.0 | 1.2 | 16 | 8.8 | 2.0 |
| Rodrigues et al., 2019 | 17 | 8.5 | 1.2 | 16 | 9.4 | 1.6 |
| Farah et al., 2018 | 14 | 7.7 | 1.12 | 16 | 8.8 | 2.00 |
| Farah et al., 2018 | 18 | 8.8 | 1.27 | 16 | 8.8 | 2.00 |
| Farah et al., 2018 | 14 | 8.5 | 1.12 | 16 | 9.4 | 1.60 |
| Farah et al., 2018 | 18 | 8.9 | 2.12 | 16 | 9.4 | 1.60 |
| Beck et al., 2013 | 15 | 7.81 | 1.16 | 15 | 7.92 | 1.20 |
| Beck et al., 2013 | 15 | 9.39 | 1.39 | 15 | 8.60 | 0.97 |
| Beck et al., 2013 | 15 | 6.81 | 0.70 | 15 | 6.55 | 0.70 |
| Yoon et al., 2019 | 17 | 9.9 | 2.1 | 18 | 10.3 | 1.4 |
| Miura et al., 2015 | 92 | 1821.0 | 311.8 | 92 | 1841.9 | 294.9 |
| Miura et al., 2015 | 108 | 1552 | 208.6 | 108 | 1641.5 | 203.6 |
| Jung et al., 2024 | 14 | 1718.82 | 215.67 | 14 | 1856.11 | 159.77 |
| Fernandez-del-Valle et al., 2018 | 6 | 6.73 | 0.94 | 5 | 6.70 | 0.82 |
| Figueroa et al., 2014 | 13 | 12.2 | 2.16 | 12 | 12.4 | 1.39 |
| Figueroa et al., 2014 | 13 | 9.4 | 1.08 | 12 | 9.7 | 1.04 |
| Figueroa et al., 2014 | 13 | 12.8 | 1.44 | 12 | 14.0 | 1.39 |
| Croymans et al., 2014 | 28 | 6.67 | 1.19 | 8 | 7.27 | 0.30 |
| Craighead et al., 2021 | 18 | 9.64 | 1.53 | 18 | 9.92 | 2.33 |
| Craighead et al., 2021 | 18 | 10.01 | 5.12 | 18 | 9.08 | 3.16 |

## ================================

## 0. 环境准备

## ================================

library(meta)

## ================================

## 1. 构建数据

## ================================

data <- data.frame(

Study = c(

"Banks et al., 2024",

"Rodrigues et al., 2019",

"Rodrigues et al., 2019",

"Farah et al., 2018",

"Farah et al., 2018",

"Farah et al., 2018",

"Farah et al., 2018",

"Beck et al., 2013",

"Beck et al., 2013",

"Beck et al., 2013",

"Yoon et al., 2019",

"Miura et al., 2015",

"Miura et al., 2015",

"Jung et al., 2024",

"Fernandez-del-Valle et al., 2018",

"Figueroa et al., 2014",

"Figueroa et al., 2014",

"Figueroa et al., 2014",

"Croymans et al., 2014",

"Craighead et al., 2021",

"Craighead et al., 2021"

),

n_e = c(

13, 17, 17, 14, 18, 14, 18, 15, 15, 15, 17,

92, 108, 14, 6, 13, 13, 13, 28, 18, 18

),

mean_e = c(

6.8, 8.0, 8.5, 7.7, 8.8, 8.5, 8.9, 7.81, 9.39, 6.81, 9.9,

1821.0, 1552.0, 1718.82, 6.73, 12.2, 9.4, 12.8, 6.67, 9.64, 10.01

),

sd_e = c(

1.10, 1.2, 1.2, 1.12, 1.27, 1.12, 2.12, 1.16, 1.39, 0.70, 2.1,

311.8, 208.6, 215.67, 0.94, 2.16, 1.08, 1.44, 1.19, 1.53, 5.12

),

n_c = c(

13, 16, 16, 16, 16, 16, 16, 15, 15, 15, 18,

92, 108, 14, 5, 12, 12, 12, 8, 18, 18

),

mean_c = c(

7.2, 8.8, 9.4, 8.8, 8.8, 9.4, 9.4, 7.92, 8.60, 6.55, 10.3,

1841.9, 1641.5, 1856.11, 6.70, 12.4, 9.7, 14.0, 7.27, 9.92, 9.08

),

sd_c = c(

0.92, 2.0, 1.6, 2.00, 2.00, 1.60, 1.60, 1.20, 0.97, 0.70, 1.4,

294.9, 203.6, 159.77, 0.82, 1.39, 1.04, 1.39, 0.30, 2.33, 3.16

)

)

## ================================

## 2. Meta 分析（随机效应）

## ================================

meta_res <- metacont(

n.e = n_e, mean.e = mean_e, sd.e = sd_e,

n.c = n_c, mean.c = mean_c, sd.c = sd_c,

studlab = Study,

data = data,

sm = "SMD",

method.smd = "Hedges",

method.tau = "REML",

method.tau.ci = "J",

comb.random = TRUE,

comb.fixed = FALSE,

prediction = TRUE

)

## ================================

## 3. 配色：渐变蓝

## ================================

pal_fn <- grDevices::colorRampPalette(c("#6BAED6", "#3182BD", "#08519C"))

pal <- pal_fn(200)

col_line <- "#0B3C5D"

map_to_col <- function(x, pal, rng = NULL) {

if (is.null(rng)) rng <- range(x, na.rm = TRUE)

if (!is.finite(diff(rng)) || diff(rng) == 0) return(rep(pal[length(pal)], length(x)))

idx <- floor((x - rng[1]) / diff(rng) * (length(pal) - 1)) + 1

pal[pmax(1, pmin(length(pal), idx))]

}

te_rng <- range(meta_res$TE, na.rm = TRUE)

col_sq_vec <- map_to_col(meta_res$TE, pal, rng = te_rng)

col_predict <- grDevices::adjustcolor(col_line, alpha.f = 0.35)

col_predict_lines <- grDevices::adjustcolor(col_line, alpha.f = 0.70)

## ================================

## 4. 绘制森林图：显示 Test for overall effect + 防挤压

## ================================

forest(

meta_res,

plotwidth = "13cm",

leftcols = c("studlab"),

rightcols = c("effect", "ci", "w.random"),

rightlabs = c("Hedge's g", "95% CI", "Weight"),

col.square = col_sq_vec,

col.square.lines = col_line,

col.study = col_sq_vec,

col.diamond = col_line,

col.diamond.lines = col_line,

col.predict = col_predict,

col.predict.lines = col_predict_lines,

fontsize = 9,

spacing = 1,

fs.hetstat = 9,

fs.axis = 9,

prediction = TRUE,

digits = 2,

print.tau2 = TRUE,

print.tau2.ci = TRUE,

print.tau = TRUE,

## ✅ 关键1：直接让 forest 打印 overall effect 的 Z 与 p（随机效应）

test.overall.random = TRUE,

## ✅ 关键2：在“总体结果”和“异质性/检验信息(x轴下方)”之间加空行，避免挤在一起

addrows.below.overall = 2,

## x轴标题直接用 forest 的 xlab（比 mtext 稳）

xlab = "Hedge's g"

)

## ================================

## 2.1 查看完整统计结果（含Q等）

## ================================

print(summary(meta_res))

## ================================

## 2.2 提取 Q + 计算 Q-test Power(%)

## （基于观察到的Q的事后/近似 achieved power）

## ================================

Q_val <- meta_res$Q

df_Q <- meta_res$df.Q

p_Q <- meta_res$pval.Q

alpha_Q <- 0.10 # 常用于Q异质性检验；如需0.05改这里

Q_crit <- qchisq(1 - alpha_Q, df = df_Q)

## 非中心参数常用近似：lambda ≈ max(0, Q - df)

lambda <- max(0, Q_val - df_Q)

Power_Qtest_pct <- 100 * (1 - pchisq(Q_crit, df = df_Q, ncp = lambda))

out_Q_power <- data.frame(

Q = Q_val,

df = df_Q,

p_Q = p_Q,

alpha = alpha_Q,

Q_crit = Q_crit,

lambda = lambda,

Power_Qtest_pct = Power_Qtest_pct

)

print(out_Q_power)

## 如果你只要两项（Q 和 Power%），用这个：

Q_and_Power <- data.frame(

Q = Q_val,

Power_Qtest_pct = Power_Qtest_pct

)

print(Q_and_Power)


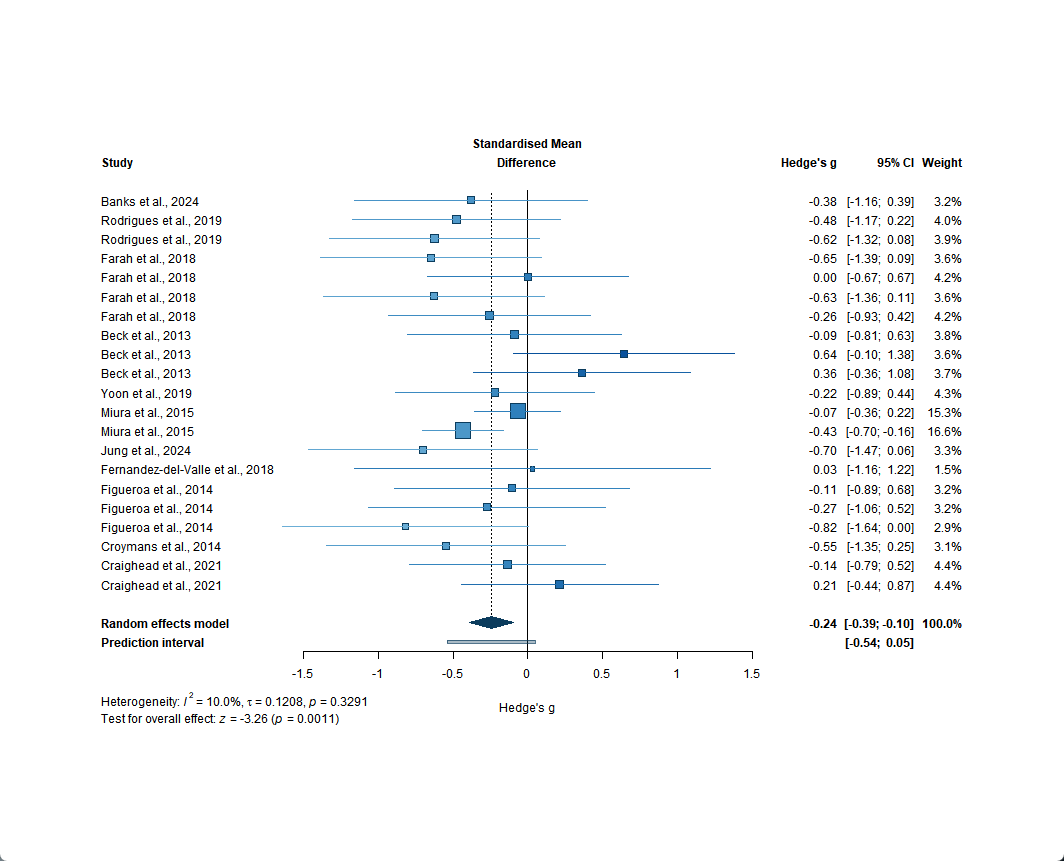

Supplement: Supplementary file 3 [file Supplementaryfile3.zip › Data/Arterial stiffness/Subgroup analysis/Intervention/RT.docx]
