# Supplementary material for: Can resistance training alone or resistance training combined with aerobic training improve arterial stiffness, endothelial function, and other vascular function indicators in adults with hypertension or overweight/obesity-related vascular risk? A systematic review and meta-analysis of randomized controlled trials
Source: Front Cardiovasc Med. 2026 Jun 24;13:1835366. doi: 10.3389/fcvm.2026.1835366 (PMC13341816; doi:10.3389/fcvm.2026.1835366)
Supplement: Supplementary file 3 [file Supplementaryfile3.zip › Data/FMD/Data.docx]

| Study | Experiment | | | Control | | |
| --- | --- | --- | --- | --- | --- | --- |
|  | Total | MEAN | SD | Total | MEAN | SD |
| Banks et al., 2024(RT-FMD) | 13 | 0.33 | 0.18 | 13 | 0.21 | 0.18 |
| Boeno et al., 2020(RT-FMD) | 15 | 8.58 | 2.37 | 12 | 6.8 | 2.37 |
| Rodrigues et al., 2019(IHT-FMD) | 17 | 0.51 | 0.21 | 16 | 0.45 | 0.12 |
| Yoon et al., 2019(IHT-FMD) | 17 | 8.2 | 3.8 | 18 | 6.1 | 2.2 |
| McGowan et al., 2007(bilateral IHT-FMD) | 7 | 4.4 | 1.59 | 9 | 2.5 | 1.50 |
| McGowan et al., 2007(unilateral IHT-FMD) | 7 | 6.6 | 3.60 | 9 | 2.5 | 1.50 |
| Jung et al., 2024(CRT-FMD) | 14 | 7.39 | 1.27 | 14 | 5.83 | 1.20 |
| Franklin et al., 2015(CRT-FMD) | 10 | 7.4 | 1.3 | 8 | 6.7 | 3.3 |
| Dobrosielski et al., 2021(RT+AT-FMD) | 51 | 7.0 | 4.1 | 51 | 7.2 | 4.1 |
| Olson et al., 2006(RT-FMD) | 15 | 8.9 | 3.49 | 15 | 5.1 | 2.32 |
| Climie et al., 2019(SRA-FMD) | 19 | 8.5 | 3.7 | 19 | 5.9 | 4.0 |
| Craighead et al., 2021(IMST-FMD) | 18 | 7.68 | 2.97 | 18 | 4.50 | 2.97 |

## ================================

## 0. 环境准备

## ================================

library(meta)

## ================================

## 1. 构建数据（已替换为 Data.docx 中的数据）

## ================================

data <- data.frame(

Study = c(

"Banks et al., 2024(RT-FMD)",

"Boeno et al., 2020(RT-FMD)",

"Rodrigues et al., 2019(IHT-FMD)",

"Yoon et al., 2019(IHT-FMD)",

"McGowan et al., 2007(bilateral IHT-FMD)",

"McGowan et al., 2007(unilateral IHT-FMD)",

"Jung et al., 2024(CRT-FMD)",

"Franklin et al., 2015(CRT-FMD)",

"Dobrosielski et al., 2021(RT+AT-FMD)",

"Olson et al., 2006(RT-FMD)",

"Climie et al., 2019(SRA-FMD)",

"Craighead et al., 2021(IMST-FMD)"

),

n_e = c(13, 15, 17, 17, 7, 7, 14, 10, 51, 15, 19, 18),

mean_e = c(0.33, 8.58, 0.51, 8.2, 4.4, 6.6, 7.39, 7.4, 7.0, 8.9, 8.5, 7.68),

sd_e = c(0.18, 2.37, 0.21, 3.8, 1.59, 3.60, 1.27, 1.3, 4.1, 3.49, 3.7, 2.97),

n_c = c(13, 12, 16, 18, 9, 9, 14, 8, 51, 15, 19, 18),

mean_c = c(0.21, 6.8, 0.45, 6.1, 2.5, 2.5, 5.83, 6.7, 7.2, 5.1, 5.9, 4.50),

sd_c = c(0.18, 2.37, 0.12, 2.2, 1.50, 1.50, 1.20, 3.3, 4.1, 2.32, 4.0, 2.97)

)

## ================================

## 2. Meta 分析（随机效应）

## ================================

meta_res <- metacont(

n.e = n_e, mean.e = mean_e, sd.e = sd_e,

n.c = n_c, mean.c = mean_c, sd.c = sd_c,

studlab = Study,

data = data,

sm = "SMD",

method.smd = "Hedges",

method.tau = "REML",

method.tau.ci = "J",

comb.random = TRUE,

comb.fixed = FALSE,

prediction = TRUE

)

## ================================

## 3. 配色：渐变蓝

## ================================

pal_fn <- grDevices::colorRampPalette(c("#6BAED6", "#3182BD", "#08519C"))

pal <- pal_fn(200)

col_line <- "#0B3C5D"

map_to_col <- function(x, pal, rng = NULL) {

if (is.null(rng)) rng <- range(x, na.rm = TRUE)

if (!is.finite(diff(rng)) || diff(rng) == 0) return(rep(pal[length(pal)], length(x)))

idx <- floor((x - rng[1]) / diff(rng) * (length(pal) - 1)) + 1

pal[pmax(1, pmin(length(pal), idx))]

}

te_rng <- range(meta_res$TE, na.rm = TRUE)

col_sq_vec <- map_to_col(meta_res$TE, pal, rng = te_rng)

col_predict <- grDevices::adjustcolor(col_line, alpha.f = 0.35)

col_predict_lines <- grDevices::adjustcolor(col_line, alpha.f = 0.70)

## ================================

## 4. 绘制森林图：显示 Test for overall effect + 防挤压

## ================================

forest(

meta_res,

plotwidth = "13cm",

leftcols = c("studlab"),

rightcols = c("effect", "ci", "w.random"),

rightlabs = c("Hedge's g", "95% CI", "Weight"),

col.square = col_sq_vec,

col.square.lines = col_line,

col.study = col_sq_vec,

col.diamond = col_line,

col.diamond.lines = col_line,

col.predict = col_predict,

col.predict.lines = col_predict_lines,

fontsize = 9,

spacing = 1,

fs.hetstat = 9,

fs.axis = 9,

prediction = TRUE,

digits = 2,

print.tau2 = TRUE,

print.tau2.ci = TRUE,

print.tau = TRUE,

test.overall.random = TRUE,

addrows.below.overall = 2,

xlab = "Hedge's g"

)
