# Supplementary material for: Can resistance training alone or resistance training combined with aerobic training improve arterial stiffness, endothelial function, and other vascular function indicators in adults with hypertension or overweight/obesity-related vascular risk? A systematic review and meta-analysis of randomized controlled trials
Source: Front Cardiovasc Med. 2026 Jun 24;13:1835366. doi: 10.3389/fcvm.2026.1835366 (PMC13341816; doi:10.3389/fcvm.2026.1835366)

| X：Frequency | Y：（effect size）Hedge's g | Weight（%） |
| --- | --- | --- |
| 3 | 0.65 | 8.0 |
| 3 | 0.73 | 8.0 |
| 3 | 0.34 | 9.3 |
| 3 | 0.67 | 9.4 |
| 3 | 1.17 | 5.2 |
| 3 | 1.48 | 4.8 |
| 3 | 1.23 | 7.7 |
| 2 | 0.28 | 6.5 |
| 3 | -0.05 | 14.4 |
| ≥2 | 1.25 | 8.0 |
| 1 | 0.66 | 9.8 |
| 6 | 1.05 | 9.1 |

# 加载必要的包

library(metafor)

# 创建数据框（已按 Frequency.docx 替换：X=Frequency, Y=Hedge's g, Weight%）

df <- data.frame(

Frequency = c("3", "3", "3", "3", "3", "3", "3", "2", "3", "≥2", "1", "6"),

g = c(

0.65, 0.73, 0.34, 0.67, 1.17, 1.48,

1.23, 0.28, -0.05, 1.25, 0.66, 1.05

),

Weight = c(

8.0, 8.0, 9.3, 9.4, 5.2, 4.8,

7.7, 6.5, 14.4, 8.0, 9.8, 9.1

)

)

# 将 Frequency 转为数值

# 对“≥2”这类写法提取其中数值用于回归

df$Frequency_num <- as.numeric(gsub("[^0-9.]", "", df$Frequency))

# 计算方差（权重为1/vi）

df$vi <- 1 / df$Weight

# 执行Meta回归分析（混合效应模型）

res <- rma(yi = g, vi = vi, mods = ~ Frequency_num, data = df)

# 提取统计结果（稳健写法：从 summary(res) 的系数表取数值）

tab <- coef(summary(res)) # estimate, se, zval, pval, ci.lb, ci.ub

beta <- round(tab[2, "estimate"], 3)

ci_lb <- round(tab[2, "ci.lb"], 3)

ci_ub <- round(tab[2, "ci.ub"], 3)

p_value <- ifelse(tab[2, "pval"] < 0.001, "< 0.001", round(tab[2, "pval"], 3))

# 绘制气泡图

regplot(

res,

mod = "Frequency_num",

pi = TRUE,

pred = TRUE,

xlab = "Frequency",

ylab = "Hedge's g",

psize = sqrt(df$Weight),

col = "black",

ci.col = "darkgray",

pi.col = "lightgray",

las = 1

)

# 添加统计结果文本

text(

x = max(df$Frequency_num) - 0.2 * (max(df$Frequency_num) - min(df$Frequency_num)),

y = max(df$g) - 0.1 * (max(df$g) - min(df$g)),

labels = paste0(

"β=", beta, "\n",

"95% CI: [", ci_lb, ", ", ci_ub, "]\n",

"P=", p_value

),

pos = 2,

cex = 1.1,

col = "black",

font = 2

)

# 添加紧凑图例

legend(

"bottomright",

legend = c("Studies", "Regression Line", "95% Confidence Interval", "95% Prediction Interval"),

pch = c(19, NA, NA, NA),

lty = c(NA, 1, NA, NA),

fill = c(NA, NA, "darkgray", "lightgray"),

border = c(NA, NA, "darkgray", "lightgray"),

col = c("gray60", "black", NA, NA),

pt.cex = 1.0,

cex = 1,

x.intersp = 1,

y.intersp = 1,

bg = "white"

)


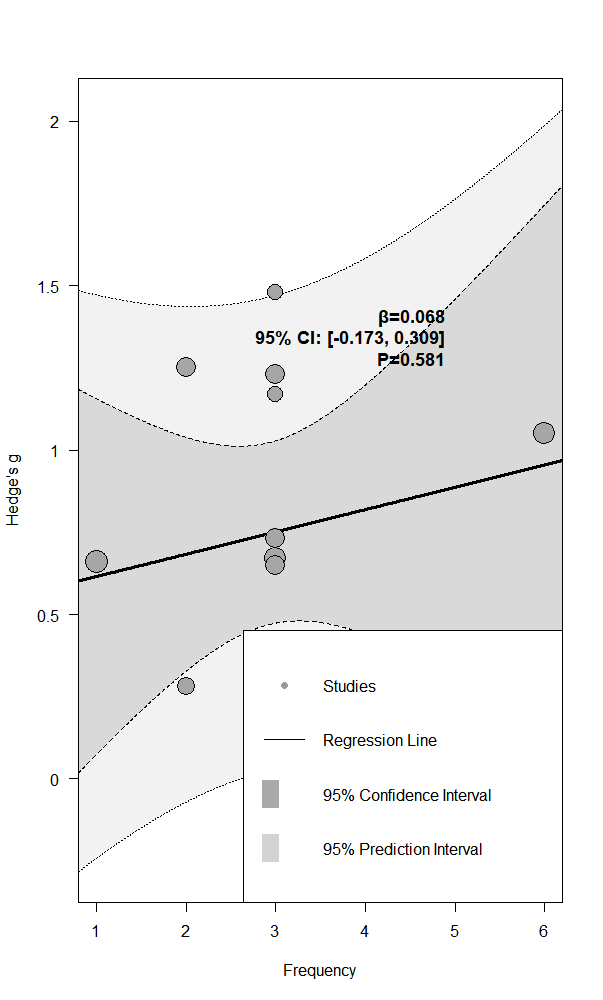

Supplement: Supplementary file 3 [file Supplementaryfile3.zip › Data/FMD/Meta-regression analysis/Frequency.docx]
