# Supplementary material for: Can resistance training alone or resistance training combined with aerobic training improve arterial stiffness, endothelial function, and other vascular function indicators in adults with hypertension or overweight/obesity-related vascular risk? A systematic review and meta-analysis of randomized controlled trials
Source: Front Cardiovasc Med. 2026 Jun 24;13:1835366. doi: 10.3389/fcvm.2026.1835366 (PMC13341816; doi:10.3389/fcvm.2026.1835366)

| Study | Experiment | | | Control | | |
| --- | --- | --- | --- | --- | --- | --- |
|  | Total | MEAN | SD | Total | MEAN | SD |
| Banks et al., 2024 | 13 | 0.33 | 0.18 | 13 | 0.21 | 0.18 |
| Boeno et al., 2020 | 15 | 8.58 | 2.37 | 12 | 6.8 | 2.37 |
| Rodrigues et al., 2019 | 17 | 0.51 | 0.21 | 16 | 0.45 | 0.12 |
| Yoon et al., 2019 | 17 | 8.2 | 3.8 | 18 | 6.1 | 2.2 |
| McGowan et al., 2007 | 7 | 4.4 | 1.59 | 9 | 2.5 | 1.50 |
| McGowan et al., 2007 | 7 | 6.6 | 3.60 | 9 | 2.5 | 1.50 |
| Jung et al., 2024 | 14 | 7.39 | 1.27 | 14 | 5.83 | 1.20 |
| Franklin et al., 2015 | 10 | 7.4 | 1.3 | 8 | 6.7 | 3.3 |
| Dobrosielski et al., 2021 | 51 | 7.0 | 4.1 | 51 | 7.2 | 4.1 |
| Olson et al., 2006 | 15 | 8.9 | 3.49 | 15 | 5.1 | 2.32 |
| Climie et al., 2019 | 19 | 8.5 | 3.7 | 19 | 5.9 | 4.0 |
| Craighead et al., 2021 | 18 | 7.68 | 2.97 | 18 | 4.50 | 2.97 |

# ================================

# 完整代码：森林图 + Egger + 漏斗图

# 配色：渐变蓝色系·深色增强版（森林图方块/研究名随TE渐变；菱形/线深蓝；漏斗图气泡同渐变蓝；漏斗背景等高线同蓝系）

# ================================

# 加载所需包

library(meta)

library(grid)

# 创建研究名称向量

study <- c(

"Banks et al., 2024",

"Boeno et al., 2020",

"Rodrigues et al., 2019",

"Yoon et al., 2019",

"McGowan et al., 2007^1^",

"McGowan et al., 2007^2^",

"Jung et al., 2024",

"Franklin et al., 2015",

"Dobrosielski et al., 2021",

"Olson et al., 2006",

"Climie et al., 2019",

"Craighead et al., 2021"

)

# 实验组和对照组数据

n1 <- c(13, 15, 17, 17, 7, 7, 14, 10, 51, 15, 19, 18)

m1 <- c(0.33, 8.58, 0.51, 8.2, 4.4, 6.6, 7.39, 7.4, 7.0, 8.9, 8.5, 7.68)

sd1 <- c(0.18, 2.37, 0.21, 3.8, 1.59, 3.60, 1.27, 1.3, 4.1, 3.49, 3.7, 2.97)

n2 <- c(13, 12, 16, 18, 9, 9, 14, 8, 51, 15, 19, 18)

m2 <- c(0.21, 6.8, 0.45, 6.1, 2.5, 2.5, 5.83, 6.7, 7.2, 5.1, 5.9, 4.50)

sd2 <- c(0.18, 2.37, 0.12, 2.2, 1.50, 1.50, 1.20, 3.3, 4.1, 2.32, 4.0, 2.97)

# Meta分析（Hedge's g）

meta_result <- metacont(

n1, m1, sd1,

n2, m2, sd2,

studlab = study,

sm = "SMD",

method.smd = "Hedges",

comb.fixed = FALSE,

comb.random = TRUE,

method.tau = "DL",

method.tau.ci = "J",

hakn = FALSE

)

# ================================

# 渐变蓝色系·深色增强版 配色函数

# ================================

pal_fn <- grDevices::colorRampPalette(c("#6BAED6", "#3182BD", "#08519C"))

pal <- pal_fn(200)

col_line <- "#0B3C5D"

map_to_col <- function(x, pal, rng = NULL) {

if (is.null(rng)) rng <- range(x, na.rm = TRUE)

if (!is.finite(diff(rng)) || diff(rng) == 0) return(rep(pal[length(pal)], length(x)))

idx <- floor((x - rng[1]) / diff(rng) * (length(pal) - 1)) + 1

pal[pmax(1, pmin(length(pal), idx))]

}

# 森林图方块/研究名的渐变色（按TE映射）

te_rng <- range(meta_result$TE, na.rm = TRUE)

col_sq_vec <- map_to_col(meta_result$TE, pal, rng = te_rng)

# 漏斗图气泡渐变色（同样按TE映射）

col_pt_vec <- col_sq_vec

# 漏斗图等高线区域（半透明蓝渐变）

col_contour_vec <- grDevices::adjustcolor(c("#6BAED6", "#3182BD", "#08519C"), alpha.f = 0.35)

# ================================

# 1) 森林图（隐藏原始数据；渐变蓝增强）

# ================================

forest(

meta_result,

sortvar = 1:length(study),

xlab = "Hedge's g (95% CI)",

leftcols = c("studlab", "effect", "ci"),

leftlabs = c("Study", "g", "95% CI"),

rightcols = FALSE,

col.square = col_sq_vec,

col.study = col_sq_vec,

col.square.lines = col_line,

col.diamond = col_line,

col.diamond.lines= col_line,

print.tau2 = TRUE,

print.tau2.ci = TRUE,

print.tau = TRUE

)

# ================================

# 2) Egger's 检验

# ================================

egger_test <- metabias(meta_result, method = "Egger", k.min = 3)

p_value <- ifelse(

egger_test$p.value < 0.001,

"P < 0.001",

paste0("P = ", round(egger_test$p.value, 3))

)

# ================================

# 3) 漏斗图（气泡也用渐变蓝；等高线区域用蓝系）

# ================================

funnel(

meta_result,

studlab = FALSE,

contour = c(0.9, 0.95, 0.99),

col.contour = col_contour_vec,

pch = 21,

bg = col_pt_vec,

col = col_line,

xlab = "Hedge's g",

main = "Funnel Plot with Egger's Test"

)

# 添加P值标注（左上角）

text(

x = min(meta_result$TE, na.rm = TRUE) + 0.2,

y = max(meta_result$seTE, na.rm = TRUE) * 0.95,

labels = p_value,

pos = 4,

cex = 1.1,

col = "black",

font = 2

)

# 控制台输出检验结果

cat(

"Egger's Linear Regression Test:\n",

"t =", round(egger_test$statistic, 3),

"| df =", egger_test$df,

"|", p_value

)


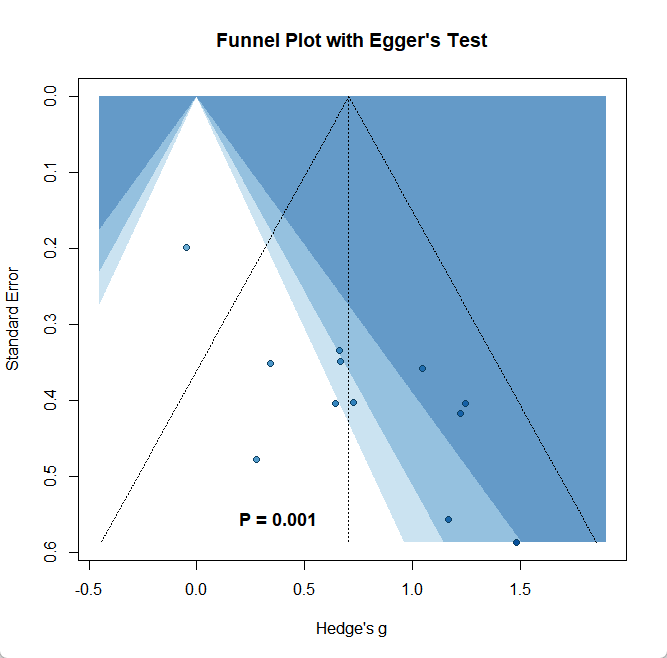

Supplement: Supplementary file 3 [file Supplementaryfile3.zip › Data/FMD/Publication Bias/Publication Bias.docx]
