# Supplementary material for: Can resistance training alone or resistance training combined with aerobic training improve arterial stiffness, endothelial function, and other vascular function indicators in adults with hypertension or overweight/obesity-related vascular risk? A systematic review and meta-analysis of randomized controlled trials
Source: Front Cardiovasc Med. 2026 Jun 24;13:1835366. doi: 10.3389/fcvm.2026.1835366 (PMC13341816; doi:10.3389/fcvm.2026.1835366)
Supplement: Supplementary file 3 [file Supplementaryfile3.zip › Data/FMD/Sensitivity Analysis/Sensitivity Analysis.docx]

| Study | Experiment | | | Control | | |
| --- | --- | --- | --- | --- | --- | --- |
|  | Total | MEAN | SD | Total | MEAN | SD |
| Banks et al., 2024(RT-FMD) | 13 | 0.33 | 0.18 | 13 | 0.21 | 0.18 |
| Boeno et al., 2020(RT-FMD) | 15 | 8.58 | 2.37 | 12 | 6.8 | 2.37 |
| Rodrigues et al., 2019(IHT-FMD) | 17 | 0.51 | 0.21 | 16 | 0.45 | 0.12 |
| Yoon et al., 2019(IHT-FMD) | 17 | 8.2 | 3.8 | 18 | 6.1 | 2.2 |
| McGowan et al., 2007(bilateral IHT-FMD) | 7 | 4.4 | 1.59 | 9 | 2.5 | 1.50 |
| McGowan et al., 2007(unilateral IHT-FMD) | 7 | 6.6 | 3.60 | 9 | 2.5 | 1.50 |
| Jung et al., 2024(CRT-FMD) | 14 | 7.39 | 1.27 | 14 | 5.83 | 1.20 |
| Franklin et al., 2015(CRT-FMD) | 10 | 7.4 | 1.3 | 8 | 6.7 | 3.3 |
| Dobrosielski et al., 2021(RT+AT-FMD) | 51 | 7.0 | 4.1 | 51 | 7.2 | 4.1 |
| Olson et al., 2006(RT-FMD) | 15 | 8.9 | 3.49 | 15 | 5.1 | 2.32 |
| Climie et al., 2019(SRA-FMD) | 19 | 8.5 | 3.7 | 19 | 5.9 | 4.0 |
| Craighead et al., 2021(IMST-FMD) | 18 | 7.68 | 2.97 | 18 | 4.50 | 2.97 |

## =========================================================

## Sensitivity analysis for FMD

## Resistance training-based interventions

## =========================================================

## Required packages

## install.packages(c("meta", "metafor"))

library(meta)

library(metafor)

## ---------------------------------------------------------

## 1. Data entry

## ---------------------------------------------------------

dat <- data.frame(

Study = c(

"Banks et al., 2024 (RT-FMD)",

"Boeno et al., 2020 (RT-FMD)",

"Rodrigues et al., 2019 (IHT-FMD)",

"Yoon et al., 2019 (IHT-FMD)",

"McGowan et al., 2007 (bilateral IHT-FMD)",

"McGowan et al., 2007 (unilateral IHT-FMD)",

"Jung et al., 2024 (CRT-FMD)",

"Franklin et al., 2015 (CRT-FMD)",

"Dobrosielski et al., 2021 (RT+AT-FMD)",

"Olson et al., 2006 (RT-FMD)",

"Climie et al., 2019 (SRA-FMD)",

"Craighead et al., 2021 (IMST-FMD)"

),

Modality = c(

"RT", "RT", "IHT", "IHT", "IHT", "IHT",

"CRT", "CRT", "RT+AT", "RT", "SRA", "IMST"

),

n.e = c(13, 15, 17, 17, 7, 7, 14, 10, 51, 15, 19, 18),

mean.e = c(0.33, 8.58, 0.51, 8.20, 4.40, 6.60, 7.39, 7.40, 7.00, 8.90, 8.50, 7.68),

sd.e = c(0.18, 2.37, 0.21, 3.80, 1.59, 3.60, 1.27, 1.30, 4.10, 3.49, 3.70, 2.97),

n.c = c(13, 12, 16, 18, 9, 9, 14, 8, 51, 15, 19, 18),

mean.c = c(0.21, 6.80, 0.45, 6.10, 2.50, 2.50, 5.83, 6.70, 7.20, 5.10, 5.90, 4.50),

sd.c = c(0.18, 2.37, 0.12, 2.20, 1.50, 1.50, 1.20, 3.30, 4.10, 2.32, 4.00, 2.97)

)

## ---------------------------------------------------------

## 2. Primary meta-analysis

## ---------------------------------------------------------

m_primary <- metacont(

n.e = n.e, mean.e = mean.e, sd.e = sd.e,

n.c = n.c, mean.c = mean.c, sd.c = sd.c,

studlab = Study,

data = dat,

sm = "SMD",

method.smd = "Hedges",

comb.fixed = FALSE,

comb.random = TRUE,

method.tau = "DL",

hakn = FALSE

)

## ---------------------------------------------------------

## 3. Sensitivity analysis 1:

## Exclude non-traditional resistance-based modalities

## Excluded: IHT, SRA, IMST

## Retained: RT, CRT, RT+AT

## ---------------------------------------------------------

dat_sens1 <- subset(dat, !(Modality %in% c("IHT", "SRA", "IMST")))

m_sens1 <- metacont(

n.e = n.e, mean.e = mean.e, sd.e = sd.e,

n.c = n.c, mean.c = mean.c, sd.c = sd.c,

studlab = Study,

data = dat_sens1,

sm = "SMD",

method.smd = "Hedges",

comb.fixed = FALSE,

comb.random = TRUE,

method.tau = "DL",

hakn = FALSE

)

## ---------------------------------------------------------

## 4. Sensitivity analysis 2:

## Strict analysis retaining conventional dynamic RT and RT+AT only

## Excluded additionally: CRT

## ---------------------------------------------------------

dat_sens2 <- subset(dat, Modality %in% c("RT", "RT+AT"))

m_sens2 <- metacont(

n.e = n.e, mean.e = mean.e, sd.e = sd.e,

n.c = n.c, mean.c = mean.c, sd.c = sd.c,

studlab = Study,

data = dat_sens2,

sm = "SMD",

method.smd = "Hedges",

comb.fixed = FALSE,

comb.random = TRUE,

method.tau = "DL",

hakn = FALSE

)

## ---------------------------------------------------------

## 5. Print summary results

## ---------------------------------------------------------

print(summary(m_primary))

print(summary(m_sens1))

print(summary(m_sens2))

summary_table <- data.frame(

Analysis = c(

"Primary analysis",

"Sensitivity 1: excluding IHT/SRA/IMST",

"Sensitivity 2: strict RT and RT+AT only"

),

k = c(m_primary$k, m_sens1$k, m_sens2$k),

Hedges_g = c(m_primary$TE.random, m_sens1$TE.random, m_sens2$TE.random),

lower_95_CI = c(m_primary$lower.random, m_sens1$lower.random, m_sens2$lower.random),

upper_95_CI = c(m_primary$upper.random, m_sens1$upper.random, m_sens2$upper.random),

p_value = c(m_primary$pval.random, m_sens1$pval.random, m_sens2$pval.random),

I2_percent = c(m_primary$I2, m_sens1$I2, m_sens2$I2) * 100

)

print(summary_table)

write.csv(summary_table, "sensitivity_analysis_FMD_summary.csv", row.names = FALSE)

## ---------------------------------------------------------

## 6. Forest plots

## ---------------------------------------------------------

png("sensitivity_forest_excluding_nontraditional_FMD.png",

width = 3200, height = 2200, res = 300)

forest(

m_sens1,

sortvar = TE,

prediction = TRUE,

print.tau2 = TRUE,

print.I2 = TRUE,

print.pval.Q = TRUE,

leftcols = c("studlab", "n.e", "n.c"),

leftlabs = c("Study", "Exercise", "Control"),

rightcols = c("effect", "ci", "w.random"),

rightlabs = c("Hedges' g", "95% CI", "Weight"),

xlab = "Hedges' g for FMD",

smlab = "Sensitivity analysis excluding non-traditional modalities"

)

dev.off()

png("sensitivity_forest_strict_RT_RTAT_FMD.png",

width = 3200, height = 1900, res = 300)

forest(

m_sens2,

sortvar = TE,

prediction = TRUE,

print.tau2 = TRUE,

print.I2 = TRUE,

print.pval.Q = TRUE,

leftcols = c("studlab", "n.e", "n.c"),

leftlabs = c("Study", "Exercise", "Control"),

rightcols = c("effect", "ci", "w.random"),

rightlabs = c("Hedges' g", "95% CI", "Weight"),

xlab = "Hedges' g for FMD",

smlab = "Strict sensitivity analysis retaining RT and RT+AT only"

)

dev.off()

## ---------------------------------------------------------

## 7. Optional: compact summary plot

## ---------------------------------------------------------

png("sensitivity_summary_FMD.png", width = 3000, height = 1500, res = 300)

op <- par(mar = c(5, 12, 4, 4))

ypos <- 3:1

plot(

summary_table$Hedges_g, ypos,

xlim = range(c(summary_table$lower_95_CI, summary_table$upper_95_CI, 0), na.rm = TRUE),

ylim = c(0.5, 3.5),

yaxt = "n",

xlab = "Hedges' g for FMD",

ylab = "",

pch = 19,

main = "Sensitivity analyses for FMD"

)

axis(2, at = ypos, labels = summary_table$Analysis, las = 1)

abline(v = 0, lty = 2)

segments(summary_table$lower_95_CI, ypos, summary_table$upper_95_CI, ypos)

for (i in seq_len(nrow(summary_table))) {

text(

x = summary_table$upper_95_CI[i],

y = ypos[i] + 0.12,

labels = sprintf("g = %.2f [%.2f, %.2f], p = %.3f",

summary_table$Hedges_g[i],

summary_table$lower_95_CI[i],

summary_table$upper_95_CI[i],

summary_table$p_value[i]),

pos = 4,

cex = 0.8

)

}

par(op)

dev.off()
