# Supplementary material for: Can resistance training alone or resistance training combined with aerobic training improve arterial stiffness, endothelial function, and other vascular function indicators in adults with hypertension or overweight/obesity-related vascular risk? A systematic review and meta-analysis of randomized controlled trials
Source: Front Cardiovasc Med. 2026 Jun 24;13:1835366. doi: 10.3389/fcvm.2026.1835366 (PMC13341816; doi:10.3389/fcvm.2026.1835366)
Supplement: Supplementary file 3 [file Supplementaryfile3.zip › Data/FMD/Subgroup analysis/Repetitions/Subgroup.docx]

| Subgroup | Hedge's g | 95% CI |
| --- | --- | --- |
| ≤ 5 | 0.94 | 0.43 to 1.46 |
| 6-15 | 0.52 | 0.04 to 0.99 |
| > 15 | 0.91 | 0.38 to 1.43 |

**## ================================**

**## 0. 环境准备**

**## ================================**

**library(meta)**

**## ================================**

**## 1. 构建数据（来自 亚组.docx：Hedge's g + 95% CI）**

**## ================================**

**data <- data.frame(**

**Study = c(**

**"≤ 5",**

**"6-15",**

**"> 15"**

**),**

**TE = c(0.94, 0.52, 0.91),**

**lower = c(0.43, 0.04, 0.38),**

**upper = c(1.46, 0.99, 1.43)**

**)**

**## 由 95% CI 反推标准误 seTE：se ≈ (upper - lower) / (2*1.96)**

**data$seTE <- (data$upper - data$lower) / (2 * 1.96)**

**## ================================**

**## 2. Meta 分析（随机效应）**

**## ================================**

**meta_res <- metagen(**

**TE = TE,**

**seTE = seTE,**

**studlab = Study,**

**data = data,**

**sm = "SMD",**

**method.tau = "REML",**

**method.tau.ci = "J",**

**comb.random = TRUE,**

**comb.fixed = FALSE,**

**prediction = TRUE**

**)**

**## ================================**

**## 3. 配色：渐变蓝**

**## ================================**

**pal_fn <- grDevices::colorRampPalette(c("#6BAED6", "#3182BD", "#08519C"))**

**pal <- pal_fn(200)**

**col_line <- "#0B3C5D"**

**map_to_col <- function(x, pal, rng = NULL) {**

**if (is.null(rng)) rng <- range(x, na.rm = TRUE)**

**if (!is.finite(diff(rng)) || diff(rng) == 0) return(rep(pal[length(pal)], length(x)))**

**idx <- floor((x - rng[1]) / diff(rng) * (length(pal) - 1)) + 1**

**pal[pmax(1, pmin(length(pal), idx))]**

**}**

**te_rng <- range(meta_res$TE, na.rm = TRUE)**

**col_sq_vec <- map_to_col(meta_res$TE, pal, rng = te_rng)**

**col_predict <- grDevices::adjustcolor(col_line, alpha.f = 0.35)**

**col_predict_lines <- grDevices::adjustcolor(col_line, alpha.f = 0.70)**

**## ================================**

**## 4. 绘制森林图：显示 Test for overall effect + 防挤压**

**## ================================**

**forest(**

**meta_res,**

**plotwidth = "13cm",**

**leftcols = c("studlab"),**

**rightcols = c("effect", "ci", "w.random"),**

**rightlabs = c("Hedge's g", "95% CI", "Weight"),**

**col.square = col_sq_vec,**

**col.square.lines = col_line,**

**col.study = col_sq_vec,**

**col.diamond = col_line,**

**col.diamond.lines = col_line,**

**col.predict = col_predict,**

**col.predict.lines = col_predict_lines,**

**fontsize = 9,**

**spacing = 1,**

**fs.hetstat = 9,**

**fs.axis = 9,**

**prediction = TRUE,**

**digits = 2,**

**print.tau2 = TRUE,**

**print.tau2.ci = TRUE,**

**print.tau = TRUE,**

**test.overall.random = TRUE,**

**addrows.below.overall = 2,**

**xlab = "Hedge's g"**

**)**
